# Trofinetide for the treatment of Rett syndrome: a randomized phase 3 study

**DOI:** 10.1038/s41591-023-02398-1

**Published:** 2023-06-08

**Authors:** Jeffrey L. Neul, Alan K. Percy, Timothy A. Benke, Elizabeth M. Berry-Kravis, Daniel G. Glaze, Eric D. Marsh, Tim Lin, Serge Stankovic, Kathie M. Bishop, James M. Youakim

**Affiliations:** 1 Vanderbilt Kennedy Center, Vanderbilt University Medical Center, Nashville, TN USA; 2 University of Alabama at Birmingham, Birmingham, AL USA; 3 Children’s Hospital of Colorado and University of Colorado School of Medicine, Aurora, CO USA; 4 Rush University Medical Center, Chicago, IL USA; 5 Texas Children’s Hospital and Baylor College of Medicine, Houston, TX USA; 6 Children’s Hospital of Philadelphia, Philadelphia, PA USA; 7 Acadia Pharmaceuticals Inc., San Diego, CA USA

**Keywords:** Neurodevelopmental disorders, Medical research

## Abstract

Rett syndrome is a rare, genetic neurodevelopmental disorder. Trofinetide is a synthetic analog of glycine–proline–glutamate, the N-terminal tripeptide of the insulin-like growth factor 1 protein, and has demonstrated clinical benefit in phase 2 studies in Rett syndrome. In this phase 3 study (https://clinicaltrials.gov identifier NCT04181723), females with Rett syndrome received twice-daily oral trofinetide (*n* = 93) or placebo (*n* = 94) for 12 weeks. For the coprimary efficacy endpoints, least squares mean (LSM) change from baseline to week 12 in the Rett Syndrome Behaviour Questionnaire for trofinetide versus placebo was −4.9 versus −1.7 (*P* = 0.0175; Cohen’s *d* effect size, 0.37), and LSM Clinical Global Impression–Improvement at week 12 was 3.5 versus 3.8 (*P* = 0.0030; effect size, 0.47). For the key secondary efficacy endpoint, LSM change from baseline to week 12 in the Communication and Symbolic Behavior Scales Developmental Profile Infant–Toddler Checklist Social Composite score was −0.1 versus −1.1 (*P* = 0.0064; effect size, 0.43). Common treatment-emergent adverse events included diarrhea (80.6% for trofinetide versus 19.1% for placebo), which was mostly mild to moderate in severity. Significant improvement for trofinetide compared with placebo was observed for the coprimary efficacy endpoints, suggesting that trofinetide provides benefit in treating the core symptoms of Rett syndrome.

## Abstract (web summary)

Results from the LAVENDER phase 3 study demonstrate that trofinetide, a synthetic analog of glycine–proline–glutamate, provides significant therapeutic benefits in the core symptoms of Rett syndrome

## Main

Rett syndrome (RTT) is a rare, genetic neurodevelopmental disorder characterized by loss of verbal communication with limited nonverbal skills, loss of fine and gross motor function (including purposeful hand use), behavioral issues, seizures, hand stereotypies and gastrointestinal problems^1,2^. Almost all cases of RTT are caused by de novo loss-of-function mutations in the X-linked gene *MECP2* encoding methyl-CpG-binding protein 2 (MeCP2), a DNA-binding protein with a role in epigenetic regulation of gene expression^3^ and deficiency of which results in abnormal neuronal maturation and plasticity^4–6^.

RTT primarily affects females (1 in 10,000–15,000 live female births)^7^, but some males are affected^8^. Individuals with the syndrome undergo apparently normal development for the first 6 months of life, with failure to reach developmental milestones between 6 and 18 months^9,10^. A period of regression follows at 12–30 months with gait dysfunction, loss of acquired hand skills and spoken language and the onset of repetitive hand stereotypies^1,10,11^. From approximately 5 years of age through adulthood, no continued skill regression has been observed, with the exception of some loss of ambulation in the teen years^1,10^. Other common symptoms include awake breathing disruptions, autonomic abnormalities, scoliosis and interest in social interaction (intense eye communication)^1,10,11^. Seizures have a lifetime prevalence in RTT of around 90%, with a highly variable course of occurrence and remission, with age of seizure onset ranging from <4 years to middle age^12^. Gastrointestinal dysfunction, including substantial constipation, gastroesophageal reflux disease and chewing and swallowing difficulties are observed in most individuals with RTT^2,13^.

Trofinetide ((2*S*)-2-{[(2*S*)-1-(2-aminoacetyl)-2-methylpyrrolidine-2-carbonyl]amino}pentanedioic acid) is a synthetic analog of glycine–proline–glutamate (GPE), a naturally occurring tripeptide in the brain that is enzymatically cleaved from insulin-like growth factor 1 (refs. ^14,15^). In the *Mecp2*-deficient mouse model of RTT, GPE partially reversed RTT-like symptoms, improved survival and enhanced synaptic morphology and function^16^. Trofinetide was designed to improve the poor pharmacokinetic profile of GPE^17^. In a phase 2 study in pediatric and adolescent females with RTT^18^, treatment with trofinetide (200 mg per kg twice daily (BID)) for 6 weeks was generally well tolerated and provided nominally statistically significant (*P* ≤ 0.05) improvements in caregiver- and clinician-assessed efficacy measures, including on the Rett Syndrome Behaviour Questionnaire (RSBQ)^19^ and the Clinical Global Impression–Improvement (CGI-I) scale^20^, compared with placebo. Clinical benefit was also observed in a previous phase 2 study in adolescent and adult females with RTT^21^.

The main objective of this phase 3 study was to investigate the efficacy, safety and tolerability of trofinetide in a larger, randomized, double-blind, placebo-controlled study in RTT.

## Results

### Demographic and baseline characteristics

**Fig. 1:**
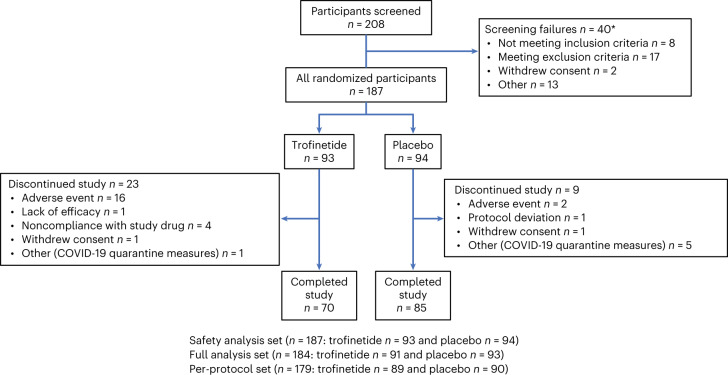
Participant disposition. Note that the three participants missing from the full analysis set (*n* = 184), who were included in the randomized analysis set (*n* = 187), had a baseline assessment but no post-baseline efficacy assessments. *208 unique participants were screened, but some were rescreened, for a total of 227 screenings. COVID-19, coronavirus disease 2019. Source data

Enrollment occurred between 29 October 2019 and 28 October 2021, with 208 participants screened and 187 participants randomized to trofinetide (*n* = 93) or placebo (*n* = 94); 155 participants (82.9%) completed the study (trofinetide, *n* = 70 (75.3%); placebo, *n* = 85 (90.4%)) (Fig. 1). Treatment groups were well balanced for demographic and baseline characteristics (Table 1). In the respective trofinetide and placebo groups, 40.9% and 41.5% of participants were administered study medication via gastrostomy tube.

**Table 1:** Baseline demographics and clinical characteristics, RTT history and history of symptoms related to RTT*

	Randomized analysis set		
	Placebo (*n* = 94)	Trofinetide (*n* = 93)	Total (*n* = 187)
Mean (s.d.) age, years	10.9 (4.57)	11.0 (4.69)	10.9 (4.62)
Age categories, *n* (%)			
5–10 years	52 (55.3)	49 (52.7)	101 (54.0)
11–15 years	24 (25.5)	25 (26.9)	49 (26.2)
16–20 years	18 (19.1)	19 (20.4)	37 (19.8)
5–11 years	55 (58.5)	53 (57.0)	108 (57.8)
12–16 years	24 (25.5)	23 (24.7)	47 (25.1)
17–20 years	15 (16.0)	17 (18.3)	32 (17.1)
Primary race, *n* (%)			
White	90 (95.7)	82 (88.2)	172 (92.0)
Black or African American	1 (1.1)	1 (1.1)	2 (1.1)
Asian	1 (1.1)	5 (5.4)	6 (3.2)
Native Hawaiian or other Pacific Islander	0	1 (1.1)	1 (0.5)
Other	2 (2.1)	4 (4.3)	6 (3.2)
Mean (s.d.) baseline RSBQ total score^a^	44.4 (12.13)	43.8 (11.42)	44.1 (11.76)
Baseline RSBQ severity, *n* (%)			
<35	25 (26.6)	23 (24.7)	48 (25.7)
≥35	69 (73.4)	70 (75.3)	139 (74.3)
Mean (s.d.) baseline CGI-S scale score^b^	4.9 (0.76)	4.9 (0.77)	4.9 (0.76)
Baseline CGI-S scale category			
1 = normal to 3 = mildly ill	0	0	0
4 = moderately ill	33 (35.1)	32 (34.4)	65 (34.8)
5 = markedly ill	42 (44.7)	38 (40.9)	80 (42.8)
6 = severely ill	18 (19.1)	23 (24.7)	41 (21.9)
7 = among the most extremely ill patients	1 (1.1)	0	1 (0.5)
Mean (s.d.) RTT-CSS score^c^ at screening	24.2 (6.68)	24.1 (6.40)	24.1 (6.53)
Mean (s.d.) baseline CSBS-DP-IT Social Composite score^d^	8.9 (3.23)	8.7 (3.32)	8.8 (3.27)
	**Safety analysis set**		
*MECP2* gene mutation severity category, *n* (%)			
Mild	37 (39.4)	30 (32.3)	67 (35.8)
Moderate	8 (8.5)	13 (14.0)	21 (11.2)
Severe	46 (48.9)	46 (49.5)	92 (49.2)
Unknown	3 (3.2)	4 (4.3)	7 (3.7)
RTT-related medical history, *n* (%)			
Constipation	74 (78.7)	70 (75.3)	144 (77.0)
Seizure	47 (50.0)	40 (43.0)	87 (46.5)
Epilepsy	16 (17.0)	20 (21.5)	36 (19.3)
Focal dyscognitive seizures	1 (1.1)	2 (2.2)	3 (1.6)
Partial seizures	1 (1.1)	2 (2.2)	3 (1.6)
Status epilepticus	2 (2.1)	1 (1.1)	3 (1.6)
Gastrostomy	34 (36.2)	37 (39.8)	71 (38.0)

*No significant differences (*P* ≤ 0.05) were detected between the study groups. *P* values for continuous variables are based on a *t*-test. *P* values for categorical variables with large cell counts are based on the *χ*^2^ test of association. *P* values for categorical variables with any small cell counts are based on Fisher’s exact test.

^a^RSBQ consists of 45 items, rated as 0 = ‘not true’, 1 = ‘somewhat or sometimes true’ or 2 = ‘very true’, that can be grouped into eight symptom domain subscales graded on a scale of 0–90 (maximum severity)^19^; the score for item 31 (‘uses eye gaze to convey feelings, needs and wishes’) was reversed in the calculations of total score and subscores for all analyses.

^b^The CGI-S scale score uses a Likert scale (1 = normal to 7 = among the most extremely ill patients)^20^.

^c^RTT-CSS is based on 13 items on a Likert scale of either 0–4 or 0–5 with a maximum total score of 58 (a higher score indicates more severe clinical status)^20^.

^d^CSBS-DP-IT Social Composite score consists of 13 caregiver-rated items, each scored 0 = ‘not yet’, 1 = ‘sometimes’ or 2 = ‘often’, and ranges from 0 to 26 (an increasing score indicates better social communication development). CGI-S, Clinical Global Impression–Severity; CSS, Clinical Severity Scale.

### Primary efficacy outcomes

**Fig. 2:**
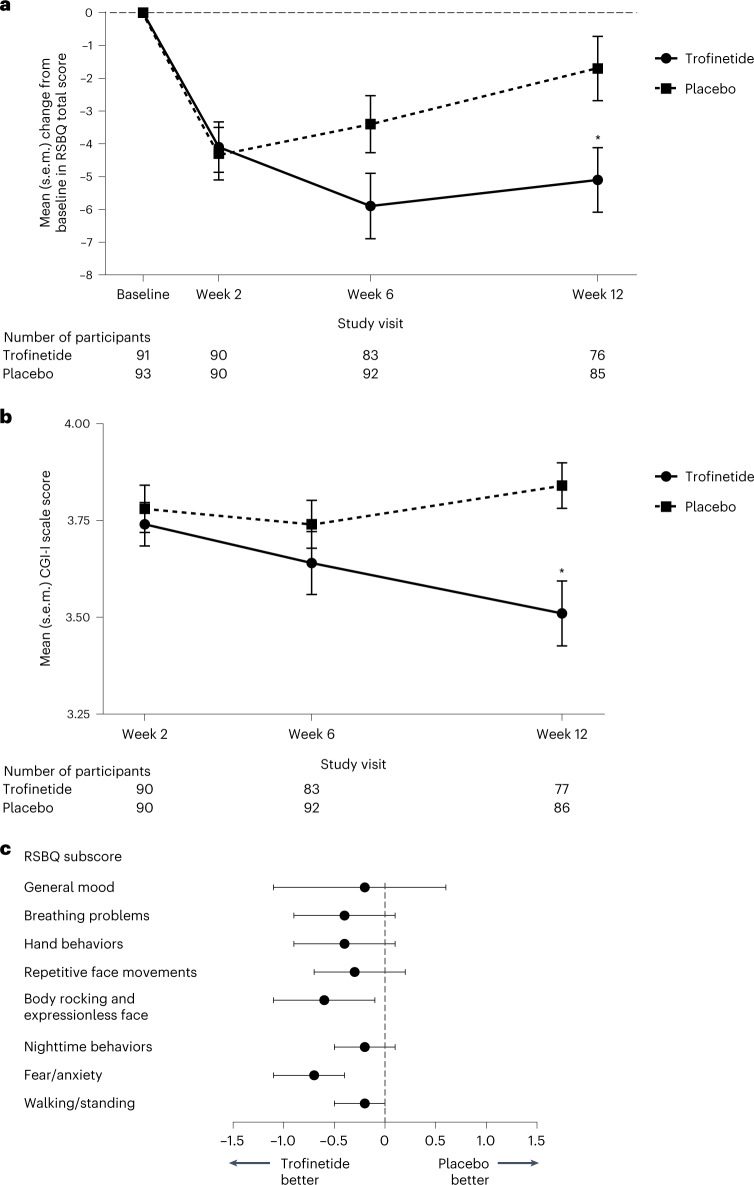
RSBQ total scores, CGI-I scale scores and RSBQ subscores. **a**, Mean (s.e.m.) change from baseline in RSBQ total score at each study visit in the full analysis set. **b**, Mean (s.e.m.) CGI-I scale score at each study visit in the full analysis set. **c**, LSM treatment differences with 95% CIs for the change in RSBQ subscores from baseline to week 12. In **a**,**b**, data are presented as mean values ± s.e.m.; asterisks at week 12 denote significance based on the LSM treatment difference from the MMRM analysis in which adjustments were made for multiple comparisons (two-sided *P* = 0.0175 and Cohen’s *d* effect size = 0.37 for the RSBQ change from baseline to week 12 and two-sided *P* = 0.0030 and Cohen’s *d* effect size = 0.47 for the CGI-I scale score at week 12). In **c**, data are presented as LSM treatment difference, and whiskers represent the lower and upper limits of the 95% CI; CI widths have not been adjusted for multiplicity. Sample size for each RSBQ subscore analysis: trofinetide (*n* = 76) and placebo (*n* = 85). Source data

**Fig. 3:**
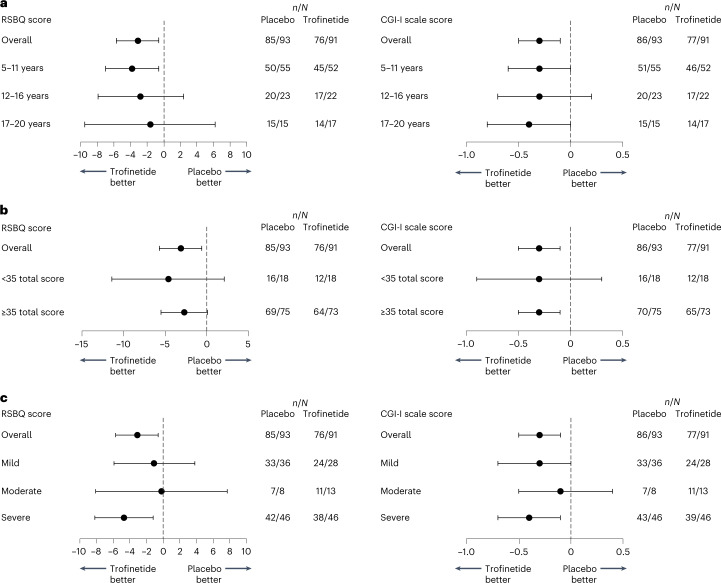
Subgroup analyses of the coprimary efficacy endpoints. **a**–**c**, LSM treatment differences with 95% CIs for the coprimary efficacy endpoints by age (**a**), baseline RSBQ severity (**b**) and category of mutation severity (**c**) based on the MMRM analysis in the full analysis set. In **a**–**c**, data are presented as LSM treatment difference, and whiskers represent the lower and upper limits of the 95% CI; CI widths have not been adjusted for multiplicity. Source data

The mean (s.e.m.) change from baseline to week 12 in the RSBQ total score was −5.1 (0.99) and −1.7 (0.98) in the trofinetide and placebo groups, respectively. Based on the mixed-effect model for repeated measure (MMRM) analysis, the LSM (s.e.m.) change from baseline to week 12 in the RSBQ total score was statistically significantly greater with trofinetide (−4.9 (0.94)) than with placebo (−1.7 (0.90)), with an LSM (s.e.m.) treatment difference of −3.1 (1.30) (95% confidence interval (CI), −5.7 to −0.6; *P* = 0.0175; Cohen’s *d* effect size, 0.37) (Fig. 2a). At week 12 in the trofinetide and placebo groups, respectively, the mean (s.e.m.) CGI-I scores were 3.5 (0.08) and 3.8 (0.06). MMRM analysis showed a statistically significant improvement with trofinetide compared with placebo at week 12, with an LSM (s.e.m.) treatment difference of −0.3 (0.10) (95% CI, −0.5 to −0.1; *P* = 0.0030; Cohen’s *d* effect size, 0.47) (Fig. 2b). Changes from baseline for all RSBQ domain subscores were directionally in favor of trofinetide (Fig. 2c). For the coprimary endpoints, the subgroup analyses showed a similar benefit with trofinetide over placebo irrespective of age, baseline RSBQ severity and category of *MECP2* mutation severity (Fig. 3a–c); the results for the sensitivity analyses and per-protocol analysis were consistent with those of the primary analyses (Extended Data Table 1).

### Key secondary efficacy outcome

The mean (s.e.m.) change from baseline to week 12 in the Communication and Symbolic Behavior Scales Developmental Profile Infant–Toddler Checklist (CSBS-DP-IT) Social Composite score was −0.1 (0.28) and −1.1 (0.28) in the trofinetide and placebo groups, respectively. MMRM analysis showed a statistically significant difference between trofinetide and placebo, with an LSM (s.e.m.) treatment difference of 1.0 (0.37) (95% CI, 0.3 to 1.7; *P* = 0.0064; Cohen’s *d* effect size, 0.43).

### Secondary efficacy outcomes

Results for the other secondary endpoints are shown in Extended Data Table 2.

### Safety analysis

In the respective trofinetide and placebo groups, at least one treatment-emergent adverse event (TEAE) was reported for 86 (92.5%) and 51 (54.3%) participants. No deaths were reported. Serious TEAEs were reported for three participants (3.2%) in each of the treatment groups (Table 2).

**Table 2:** Summary of TEAEs, the most common TEAEs (≥5% in any group) and by severity in the trofinetide and placebo groups (safety analysis set)

TEAEs and preferred term, *n* (%)	Placebo (*n* = 94)			Trofinetide (*n* = 93)			*P* value*
Any TEAE	51 (54.3)			86 (92.5)			<0.0001
Serious TEAE^a^	3 (3.2)			3 (3.2)			0.9894
TEAE leading to drug withdrawal	2 (2.1)			16 (17.2)			0.0005
Fatal TEAE	–			–			–
TEAEs reported in ≥5% of participants in any group							
Diarrhea	18 (19.1)			75 (80.6)			<0.0001
Vomiting	9 (9.6)			25 (26.9)			0.0022
Seizure	5 (5.3)			8 (8.6)			0.3775
Pyrexia	4 (4.3)			8 (8.6)			0.2252
Decreased appetite	2 (2.1)			5 (5.4)			0.2419
Irritability	–			6 (6.5)			–
TEAEs reported in ≥5% of participants in any group by severity							
	**Mild**	**Moderate**	**Severe**	**Mild**	**Moderate**	**Severe**	
Diarrhea	15 (16.0)	3 (3.2)	–	39 (41.9)	34 (36.6)	2 (2.2)	
Vomiting	8 (8.5)	1 (1.1)	–	18 (19.4)	6 (6.5)	1 (1.1)	
Seizure	3 (3.2)	2 (2.1)	–	3 (3.2)	5 (5.4)	–	
Pyrexia	2 (2.1)	2 (2.1)	–	7 (7.5)	1 (1.1)	–	
Decreased appetite	1 (1.1)	1 (1.1)	–	2 (2.2)	3 (3.2)	–	
Irritability	–	–	–	3 (3.2)	2 (2.2)	1 (1.1)	

*Two-sided *P* values were based on a post hoc analysis using the *χ*^2^ test of association. *P* values ≤ 0.05 denote nominal statistical significance.

A TEAE is an adverse event with onset date on or after the first study dose date and no later than the last study dose date +30 days. TEAEs were coded using the *Medical Dictionary for Regulatory Activities* version 24.0. A participant may have more than one TEAE per preferred term, but a participant is counted at most once per preferred term. Adverse event severity was graded as mild (easily tolerated, minimal discomfort), moderate (interferes with everyday activities) or severe (incapacitating and/or preventing normal everyday activities).

^a^Serious TEAEs were bacteremia, urinary tract infection and bronchiolitis (*n* = 1), COVID-19 pneumonia (*n* = 1) and seizure (*n* = 1) in the participants treated with trofinetide; and respiratory distress (*n* = 1), constipation (*n* = 1) and pneumatosis intestinalis (*n* = 1) in the participants treated with placebo.

The most common TEAEs in the trofinetide and placebo groups were diarrhea (80.6% and 19.1%, respectively) and vomiting (26.9% and 9.6%, respectively); of the TEAEs in the trofinetide group, 97.3% and 96.0% of diarrhea and vomiting TEAEs, respectively, were mild to moderate in severity (Table 2). Eighteen participants withdrew due to a TEAE (trofinetide, *n* = 16 (17.2%); placebo, *n* = 2 (2.1%)), with diarrhea being the primary TEAE leading to discontinuation (trofinetide, *n* = 12 (12.9%)) (Extended Data Table 3).

**Extended Data Fig. 1:**
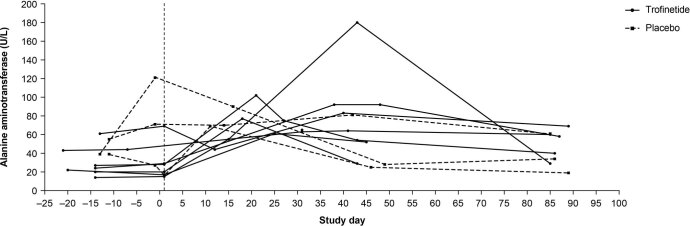
Alanine Aminotransferase Values. Figure footnote: The dashed vertical line at day 1 indicates when study treatment was initiated. Source data

Changes in laboratory tests, electrocardiograms and vital signs were generally small and similar in the treatment groups; none were considered clinically meaningful. Small, transient changes in alanine aminotransferase values were reported in seven of 92 (7.6%) and three of 93 (3.2%) participants in the trofinetide and placebo groups, respectively (Extended Data Fig. 1). These changes were not associated with notable changes in other liver function tests, and no instances met Hy’s law criteria^22^. The most frequently used concomitant medications in the trofinetide and placebo groups were antiseizure medication (64.5% and 72.3%, respectively) and drugs for constipation (60.2% and 70.2%); antipropulsives (that is, loperamide) were used more frequently in the trofinetide group (50.5% versus 3.2%), consistent with the treatment of diarrhea.

### Post hoc efficacy analyses

**Extended Data Fig. 2:**
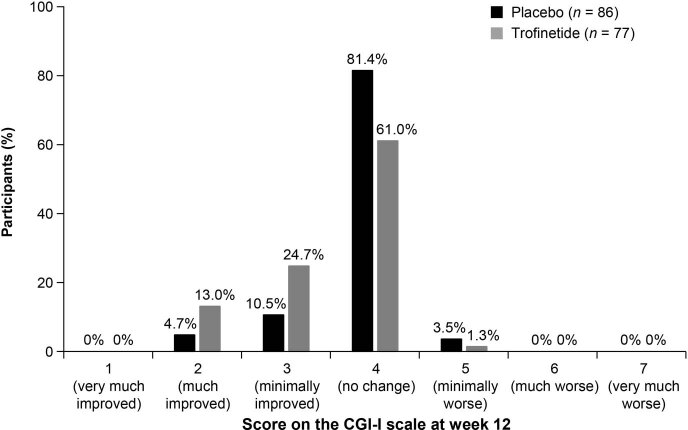
Score on the CGI-I Scale at Week 12. Figure footnote: CGI-I denotes Clinical Global Impression-Improvement. Source data

The results for the coprimary endpoints were comparable irrespective of diarrhea TEAE status (Extended Data Table 4). CGI-I responder rates (defined as CGI-I score ≤3 at week 12) were greater in the trofinetide group than in the placebo group (37.7% versus 15.2%; Extended Data Fig. 2).

## Discussion

In this phase 3 study in a large cohort of girls and women 5–20 years of age with RTT, trofinetide demonstrated a statistically significant improvement over placebo for both the coprimary and key secondary efficacy endpoints. Treatment with trofinetide improved key symptoms of the syndrome from the perspective of both the caregiver (RSBQ) and clinician (CGI-I). All RSBQ subscores were directionally in favor of trofinetide, suggesting broad improvement across key symptoms of the syndrome.

Cohen’s *d* effect sizes for the coprimary and key secondary endpoints fell in the 0.4–0.5 range (0.37 for the RSBQ, 0.47 for the CGI-I scale and 0.43 for the CSBS-DP-IT Social Composite score), suggesting that the findings of treatment benefit with trofinetide are consistent and, given that Cohen’s *d* effect sizes within this range are considered medium^23^, clinically meaningful.

The efficacy endpoints are complementary and reflect functionally important dimensions of RTT, including the ability to communicate. The RSBQ shows correlations with functioning, is validated across a range of ages (2–47 years) in RTT^24–26^ and is the most widely used instrument in RTT studies. As a clinician rating, the CGI-I scale provides clinical meaningfulness to the caregiver-rated coprimary endpoint and has been widely used in clinical trials of RTT and other neurodevelopmental disorders^18,21,27–31^. In this study, CGI-I scale ratings were assessed using RTT-specific anchors across major symptom areas that were developed to improve trial outcomes^20^, and an effort was made to standardize the CGI-I scale rating by independently rating case vignettes to fidelity as compared with a gold-standard rating^32^. Communication is one of the most important concerns for caregivers in RTT^33^, and the items comprising the CSBS-DP-IT Social Composite score are the most commonly used communication modalities by individuals with RTT.

Mild or moderate diarrhea was frequently associated with trofinetide and was responsible for the majority of discontinuations due to TEAEs; however, diarrhea was self-limited and resolved soon after withdrawal of trofinetide. The implementation of a diarrhea-management plan partway through the study, which involved the adjustment or discontinuation of laxative medications commonly taken for RTT-associated constipation, the initiation of fiber supplements and antidiarrheal medication and dose reduction or interruption of trofinetide, if necessary, appeared to mitigate this risk, as 75% of participants receiving trofinetide completed the study. Furthermore, analyses indicate that the risk of functional unblinding due to an imbalance of TEAEs of diarrhea did not bias the efficacy data in favor of trofinetide. Given that most participants were using concomitant antiseizure medication, many of which cause changes in liver enzymes^34^, the minimal effect on liver enzymes in this study does not preclude the use of trofinetide with these drugs.

The exclusion of individuals without a documented disease-causing *MECP2* mutation, males and individuals <5 and >20 years of age are limitations of the study and were based on considerations of study design to reduce variability in the population sample. Males with RTT were not enrolled due to the rarity of cases and variable phenotype in these individuals^8^. Although the study enrolled females exclusively, based on the underlying pathophysiology of RTT and the biological effects of trofinetide, the results should be applicable to the fewer males with RTT as well. Adults >20 years of age were not included due to the challenge of controlling for wide discrepancies in services available to individuals in the United States after they are no longer eligible for services through the educational system. However, similar efficacy is anticipated in older individuals, given the benefit observed in the phase 2 study that included individuals 15–44 years of age^21^ and the age subgroup analysis results in this study. The primary reason for maintaining an age cutoff of ≥5 years was in consideration of the variable early developmental regression in this age range. An ongoing study (https://clinicaltrials.gov identifier NCT04988867) is investigating the safety and pharmacokinetics of trofinetide in individuals with RTT as young as 2 years of age^35^. Of the 187 participants in the LAVENDER study, 154 elected to roll over to the open-label LILAC extension study (NCT04279314) and may be eligible to enter the follow-up LILAC-2 extension study (NCT04776746); both will inform on the long-term safety of trofinetide.

In conclusion, statistically significant differences were demonstrated between trofinetide and placebo for efficacy endpoints relevant to RTT, suggesting that trofinetide is potentially capable of modifying core symptoms consistent with the underlying pathophysiology of the syndrome. Furthermore, this study demonstrated an acceptable safety profile for trofinetide. When we evaluate the benefit versus risk associated with trofinetide, it is important to consider the medium effect size that was demonstrated for the efficacy endpoints, which can be interpreted as clinically meaningful, particularly as this is a rare disease with a high burden for patients and families. When we consider the risk element, it is important to note that diarrhea and vomiting were issues of tolerability, not safety. Almost all TEAEs of diarrhea and vomiting were mild or moderate in severity and can be managed with appropriate interventions. Given that numerous phase 2 and 3 studies in neurodevelopmental disorders including RTT have failed to meet efficacy endpoints^36,37^, these findings represent the first time treatment of a neurodevelopmental disorder has been shown to be beneficial in a large, controlled study and provides hope for a meaningful therapeutic development to treat RTT.

## Methods

### Study design

The study design and methods have been published previously^32^. In this randomized, parallel-group, placebo-controlled study conducted at 21 sites in the United States, participants were stratified by age (5–10, 11–15 and 16–20 years) and baseline RSBQ severity (<35 and ≥35 total score) and randomized 1:1 to trofinetide or placebo using an interactive response technology system via a pre-generated permuted-block randomization schedule. The sponsor, participants, caregivers and clinicians were blinded to treatment assignment via restriction to treatment codes and the identical appearance of the study drug and placebo.

A single dose level of trofinetide was tested using weight-based dosing to achieve the target exposure identified based on the results of the previous phase 2 study^18^. Trofinetide was given at 30 ml (6 g), 40 ml (8 g), 50 ml (10 g) or 60 ml (12 g) BID orally or by gastrostomy tube for participants weighing 12–20, >20–35, >35–50 and >50 kg, respectively (equivalent to a range of 200–500 mg per kg BID).

The study included a screening period of ≤3 weeks, a 12-week double-blind treatment period and a 30-day safety follow-up for participants who did not continue into the open-label extension study (https://clinicaltrials.gov, NCT04279314). The study was conducted in compliance with guidelines from the International Council for Harmonisation (Good Clinical Practice), the Declaration of Helsinki and the Health Insurance Portability and Accountability Act. The protocol was approved by central (WCG IRB) and local institutional review boards. Before screening, informed consent was obtained from the parent or guardian on behalf of the participant.

### Study population

Girls and women 5–20 years of age with RTT, a score of 10–36 on the RTT Clinical Severity Scale^20^ and a CGI-S score^20^ of ≥4 (moderate) were included. Eligible participants were at least 6 months after regression at screening (that is, no loss or degradation in ambulation, hand function, speech or nonverbal communicative or social skills within 6 months of screening) and had a stable pattern of seizures or no seizures, within 8 weeks of screening. Key exclusion criteria were current clinically significant cardiovascular, endocrine, renal, hepatic, respiratory or gastrointestinal disease or major surgery planned during the study; treatment with insulin, IGF-1 or growth hormone within 12 weeks of baseline; known history or symptoms of long QT syndrome; and QTcF interval >450 ms, history of risk factor for torsades de pointes or clinically meaningful QT prolongation deemed to increase risk. Full inclusion and exclusion criteria are listed in Supplementary Table 1.

### Intervention

Trofinetide (200 mg ml^−1^ solution) or matching placebo was administered orally or by gastrostomy tube BID (doses at least 8 h apart).

### Assessments

Coprimary and key secondary efficacy assessments using the RSBQ, the CGI-I scale and the CSBS-DP-IT Social Composite score were completed at baseline (except the CGI-I scale) and at each visit (weeks 2, 6 and 12 (or end of treatment)). The RSBQ is a caregiver-completed scale assessing key symptoms of RTT^19^ and includes 45 items (rated as 0 = ‘not true’, 1 = ‘somewhat or sometimes true’ or 2 = ‘very true’) that can be grouped into eight symptom domain subscales. The score for item 31 (‘uses eye gaze to convey feelings, needs and wishes’) was reversed in the calculations of total score and subscores for all analyses. The CGI-I scale is a clinician rating of global clinical change using a seven-point scale with RTT-specific anchors^20^. The CSBS-DP-IT Social Composite score is derived from the Communication and Symbolic Behavior Scales Developmental Profile, originally developed to assess communication and social interaction skills in young children^38^, and can be used for older children with developmental delay^39,40^. The CSBS-DP-IT Social Composite score consists of 13 caregiver-rated items, each scored 0 = ‘not yet’, 1 = ‘sometimes’ or 2 = ‘often’. Safety assessments included TEAEs, clinical laboratory assessments, vital signs and electrocardiograms. A full description of the schedule of study procedures is described in Supplementary Table 2.

### Efficacy endpoints

Coprimary endpoints were the change from baseline to week 12 in RSBQ total score and the CGI-I scale score at week 12. The key secondary endpoint was the change from baseline to week 12 in the CSBS-DP-IT Social Composite score. A prespecified subgroup analysis examined treatment effects by age, baseline RSBQ severity and *MECP2* mutation severity as categorized according to the RTT Natural History Study^41^.

### Post hoc efficacy analyses

Two additional efficacy analyses were conducted post hoc: CGI-I scale responders (scores ≤3) at week 12 and coprimary endpoints assessed in the presence or absence of the most commonly reported TEAE of diarrhea.

### Statistical analysis

A sample size of 184 participants (92 per group) was planned to provide 90% power for both coprimary endpoints combined with a two-sided significance level of 0.05. Efficacy was assessed in the full analysis set (received at least one dose and had a baseline value and at least one post-baseline value for the RSBQ or the CGI-I score); the safety analysis set consisted of participants who received at least one dose.

Coprimary and key secondary efficacy endpoints were analyzed using the MMRM method assuming data missing at random. The MMRM included randomization strata of age group and baseline RSBQ severity score, baseline RSBQ (for RSBQ analysis), baseline CGI-S (for CGI-I scale analysis) and baseline CSBS-DP-IT Social Composite score (for the key secondary endpoint), treatment, visit, treatment-by-visit interaction and baseline-by-visit interaction as fixed effects and participant as a random effect; an unstructured covariance matrix modeled within-participant errors. The Kenward–Roger method was used for calculating denominator degrees of freedom for tests of fixed effects. Each coprimary endpoint was considered positive if *P* ≤ 0.05, and both must be positive for the study to be positive. If both coprimary endpoints were positive, the key secondary endpoint was also considered positive if *P* ≤ 0.05 and was statistically controlled for type 1 error at 5% through a hierarchical sequential gatekeeper procedure. Effect size was determined with Cohen’s *d*^23^. Sensitivity analyses of the coprimary endpoints included multiple imputations based on pattern-mixture models assuming missing not at random using the analysis of covariance method, the use of actual derived baseline values for randomization strata (MMRM) and visits impacted by COVID-19 (analysis of covariance); a supportive analysis (MMRM) used the per-protocol analysis set. Possible intercurrent events included treatment discontinuation not due to COVID-19, treatment discontinuation due to COVID-19, COVID-19 events leading to intermediate missing data, non-COVID-19 events leading to intermediate missing data and remote assessments (regardless of COVID-19 or not). Observations on the coprimary efficacy endpoints were used regardless of the occurrence of intercurrent events. Alternative approaches to handling intercurrent events are addressed in sensitivity analyses. The following sensitivity analyses of the coprimary efficacy endpoints were planned and conducted to account for intercurrent events of treatment discontinuations and missing assessments. For the pattern-mixture models assuming missing not at random, the sensitivity analysis was implemented for the full analysis set using multiple imputations based on the distribution of placebo group responses over time. The underlying assumption is that missing data due to early withdrawal of participants evolves in the same way as the data for placebo-treated participants who remain in the study. For missing data due to COVID-19, this sensitivity analysis operates under the assumption that missing data after withdrawal due to COVID-19 are missing at random, while missing data after withdrawal not due to COVID-19 are not missing at random and are assumed to evolve in the same manner as for placebo-treated participants who remain in the study. Statistical analyses were performed using version 9.4 of SAS. The statistical analysis plan and protocol are available at Protocol Exchange.

### Reporting summary

Further information on research design is available in the Nature Portfolio Reporting Summary linked to this article.

## Online content

Any methods, additional references, Nature Portfolio reporting summaries, source data, extended data, supplementary information, acknowledgements, peer review information; details of author contributions and competing interests; and statements of data and code availability are available at 10.1038/s41591-023-02398-1.

## Supplementary information

Supplementary Information Supplementary Tables 1 and 2.

Reporting Summary

Supplementary Data 1 Final version of the statistical analysis plan.

Supplementary Data 2 Final version of the study protocol.

## Source data

Source Data Fig. 1 Clinical study report.

Source Data Fig. 2 Clinical study report.

Source Data Fig. 3 Clinical study report.

Source Data Extended Data Fig. 1 Clinical study report.

Source Data Extended Data Fig. 2 Clinical study report.

Source Data Extended Data Table 1 Clinical study report.

Source Data Extended Data Table 2 Clinical study report.

Source Data Extended Data Table 3 Clinical study report.

Source Data Extended Data Table 4 Clinical study report.

## Figures and Tables

**Extended Data Table 1:**
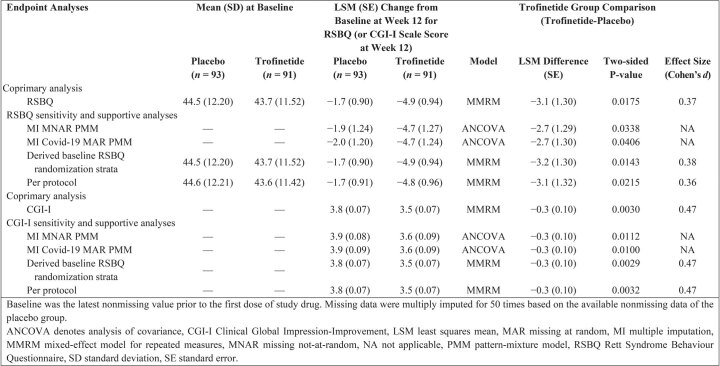
Primary Analysis, Sensitivity Analysis (Actual Derived Baseline Values for Randomization Strata, MI for MNAR PMM, MI for Covid-19 MAR PMM) and Supportive Analyses (Per Protocol) of the Coprimary Endpoints Source data

**Extended Data Table 2:**
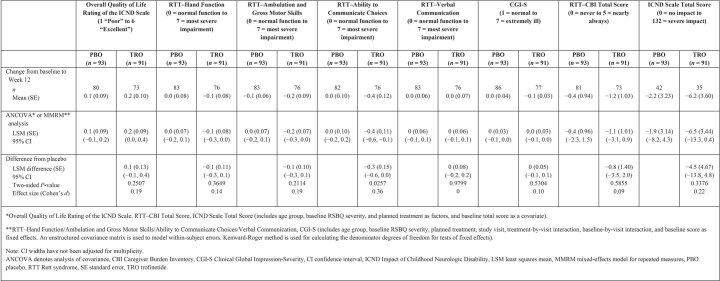
Change From Baseline to Week 12 in the Other Secondary Endpoints (Full Analysis Set) Source data

**Extended Data Table 3:**
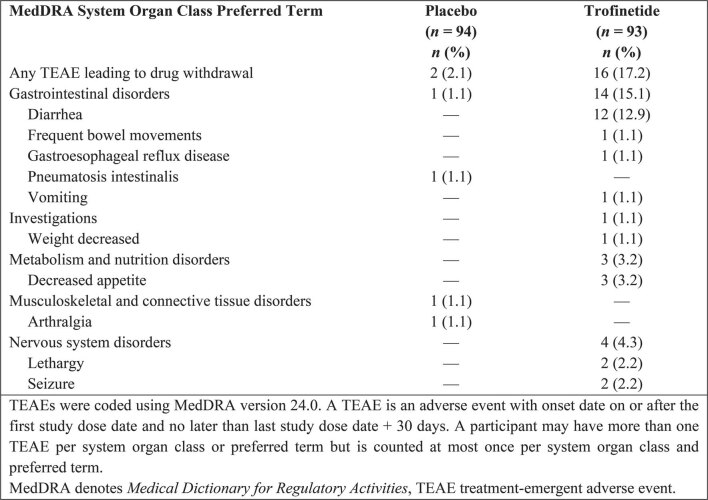
Treatment-Emergent Adverse Events Leading to Drug Withdrawal (Safety Analysis Set) Source data

**Extended Data Table 4:**
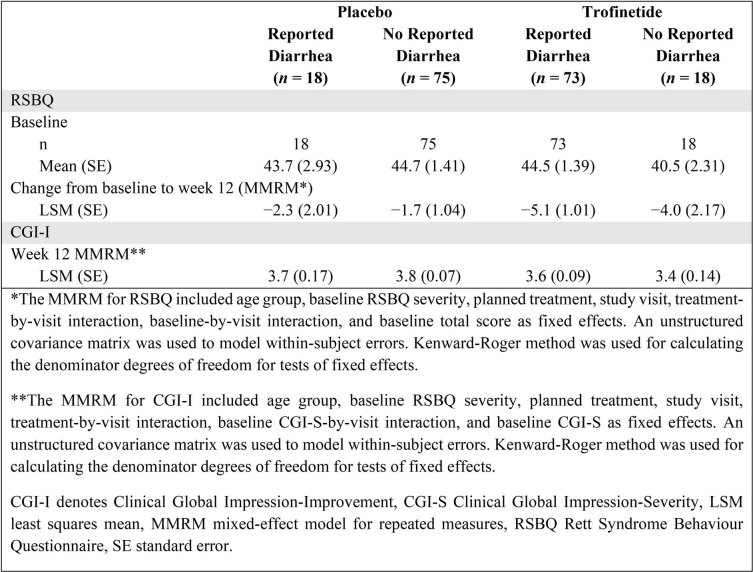
RSBQ Total Score Change From Baseline at Week 12 and CGI-I Scale Score at Week 12 in Participants With and Without Diarrhea (Full Analysis Set) Source data

## Data Availability

This clinical trial was sponsored by Acadia Pharmaceuticals. Acadia supports data sharing consistent with the Principles for Responsible Clinical Trial Data Sharing and International Committee of Medical Journal Editors’ recommendations. Acadia shares data from completed clinical trials through public registries (https://clinicaltrials.gov), presentation at scientific congresses and through open access in peer-reviewed journals. Clinical study results from this study were submitted to https://clinicaltrials.gov in April 2023. Additional, related information necessary to appraise the quality and robustness of the findings (study protocol, statistical analysis plan) is available in the Supplementary Information. The authors will provide access to individual-deidentified participant-level data that underlie the data presented in this paper, including data dictionaries, the study protocol and other relevant information, to any researcher who provides a methodologically sound proposal for academic purposes to interpret, verify and extend research in the article beginning 6 months and ending 5 years after article publication. Requests for the ‘minimum dataset’ should go through Acadia Medical Information and will be reviewed by the sponsor (Acadia) to verify whether the request is subject to any intellectual property or confidentiality obligations. For additional information, please contact Acadia Medical Information at medicalinformation@acadia-pharm.com. Source data are provided with this paper.
