# Supplementary material for: Trofinetide for the treatment of Rett syndrome: a randomized phase 3 study
Source: Nat Med. 2023 Jun 8;29(6):1468–75. doi: 10.1038/s41591-023-02398-1 (PMC10287558; doi:10.1038/s41591-023-02398-1)
Supplement: Supplementary file 1 — Supplementary Tables 1 and 2. [file 41591_2023_2398_MOESM1_ESM.pdf]

nature medicine

Article https://doi.org/10.1038/s41591-023-02398-1

Trofinetide for the treatment of Rett syndrome: a randomized phase 3 study

In the format provided by the authors and unedited

SUPPLEMENTARY INFORMATION

Supplementary Table S1: Inclusion and Exclusion Criteria.

INCLUSION CRITERIA

- Informed consent prior to the conduct of any study procedures is required as follows:
    - For subjects who are minors: written informed consent will be obtained from the LAR. The subject should provide written or oral assent if deemed able by the Investigator. The process of obtaining informed consent will be conducted in accordance with IRB or EC policy and applicable local law.
    - For subjects who are not minors: written informed consent will be obtained from the LAR or the subject if deemed able by the Investigator. If the subject is deemed not able to provide consent, the subject should provide written or oral assent if deemed able by the Investigator. The process of obtaining informed consent will be conducted in accordance with IRB or EC policy and applicable local law.
    - The subject's caregiver must also provide written informed consent regarding their participation in the study prior to participating in any study procedures.
- Female subjects 5 to 20 years of age, inclusive, at Screening.
- Body weight ≥12 kg at Screening.
- Can swallow the study medication provided as a liquid solution or can take it by gastrostomy tube (G-tube).
- The subject's caregiver is English-speaking and has sufficient language skills to complete the caregiver assessments.

Diagnosis

- Has classic/typical Rett syndrome.
- Has a documented disease-causing mutation in the *MECP2* gene.
- Is post-regression at Screening, defined as:
    - No loss or degradation of ambulation (including gait, coordination, independence of walking/standing) within 6 months of Screening
    - No loss or degradation of hand function within 6 months of Screening
    - No loss or degradation of speech (including babbling, words or previously developed communicative vocalizations) within 6 months of Screening
    - No loss or degradation of nonverbal communicative or social skills (including eye gaze, using body to indicate communicative intent, social attentiveness) within 6 months of Screening

- Has a severity rating of 10 to 36, inclusive, on the Rett Syndrome Clinical Severity Scale (RTT-CSS) at Screening.
- Has a Clinical Global Impression-Severity (CGI-S) score of ≥4 at Screening and Baseline.

Concomitant Treatment

- If the subject is taking or was taking an anticonvulsant or any other psychoactive medication (including cannabinoids):
  - The treatment regimen has been stable for at least 4 weeks before Baseline and there is no current plan to change the dose, or
  - If the medication was discontinued, the discontinuation has occurred no fewer than 2 weeks or 5 half-lives (whichever is greater) before Baseline.
- If the subject is taking or was taking any other medication daily for chronic illness (not including antibiotics, pain relievers, and laxatives):
  - The treatment regimen of the medication has been stable for at least 4 weeks before Baseline, and there is no current plan to change the dose, or
  - If the medication was discontinued, the discontinuation has occurred no fewer than 2 weeks or 5 half-lives (whichever is greater) before Baseline.
- If the subject is receiving or was receiving a nonpharmacologic somatic treatment (e.g., a ketogenic diet or vagal nerve stimulation):
  - The treatment regimen has been stable for at least 4 weeks before Baseline, and there is no current plan to change the treatment, or
  - If the treatment was discontinued, the discontinuation has occurred no fewer than 2 weeks before Baseline.
- If the subject is receiving or was receiving nonpharmacologic treatments such as an educational, behavioral, physical, occupational, or speech therapy:
  - The treatment regimen has been stable for at least 4 weeks before Baseline, and there is no current plan to change the treatment (note: changes to a treatment regimen that are due to school schedules or are otherwise seasonally related are not exclusionary), or
  - If the treatment was discontinued, the discontinuation has occurred no fewer than 2 weeks before Baseline.

Seizures

- Has a stable pattern of seizures, or has had no seizures, within 8 weeks of Screening.

Childbearing Potential

- Subjects of childbearing potential must abstain from sexual activity for the duration of the study and for at least 30 days thereafter. If a subject is sexually active or becomes sexually active during the study, she must use 2 clinically acceptable methods of contraception (e.g., oral, intrauterine device, diaphragm plus spermicide, injectable, transdermal, or implantable contraception) for the duration of the study and for at least 30 days thereafter. Subject must not be pregnant or breastfeeding.

Place of Residence

- Subject and caregiver(s) must reside at a location to which study drug can be delivered and have been at their present residence for at least 3 months prior to Screening.

EXCLUSION CRITERIA

Concomitant Treatment

- Has been treated with growth hormone within 12 weeks of Baseline.
- Has been treated with insulin-like growth factor 1 within 12 weeks of Baseline.
- Has been treated with insulin within 12 weeks of Baseline.

Medical Conditions Other Than Rett Syndrome

- Has current clinically significant cardiovascular, endocrine (such as hypo- or hyperthyroidism, Type 1 diabetes mellitus, or uncontrolled Type 2 diabetes mellitus), renal, hepatic, respiratory, or gastrointestinal disease (such as celiac disease or inflammatory bowel disease) or has major surgery planned during the study.
- Has a history of, or current, cerebrovascular disease or brain trauma.
- Has significant, uncorrected visual or uncorrected hearing impairment.
- Has a history of, or current, malignancy.

Laboratory Studies, Vital Signs, and Electrocardiogram

- Has a clinically significant abnormal laboratory value at Screening. Laboratory testing may be repeated during the Screening period with agreement of the Medical Monitor.
- Has serum potassium below the normal range for the subject (according to the central laboratory) at Screening. Serum potassium may be repeated during the Screening period with the agreement of the Medical Monitor.
- Has a hemoglobin A1C (HbA1c) >7% at Screening.

- Has a thyroid-stimulating hormone value outside the normal range for the subject (according to the central laboratory) at Screening.
- Has clinically significant abnormality in vital signs at Screening or Baseline.
- Has any of the following:
    - QTcF interval of >450 ms at Screening or Baseline (before dosing).
    - History of a risk factor for torsades de pointes (e.g., heart failure or family history of long QT syndrome).
    - History of clinically significant QT prolongation that is deemed to put the subject at increased risk of clinically significant QT prolongation.
- Has any other clinically significant finding on ECG at Screening or Baseline (before dosing).
- Has a positive pregnancy test at Screening.

Other Criteria

- Has a significant sensitivity or allergic reaction to trofinetide or its excipients.
- Has participated in another interventional clinical study within 30 days prior to Screening.
- Is judged by the Investigator or the Medical Monitor to be inappropriate for the study for any reason.

EC ethics committee, ECG electrocardiogram, HbA1C glycosylated hemoglobin, IRB institutional review board, LAR legally acceptable representative, *MECP2* methyl-CpG-binding protein 2, QTcF corrected QT interval using Fridericia's correction method**.**

Supplementary Table S2: Schedule of Events and Assessments

**Period**	**Screening**	**Baseline**	**Double blind treatment**			**Safety follow up**[b]
**Visit week**		**0**	**2**[a]	**6**	**12/EOT/ET**	**EOT/ET + 30 days**
**Visit number**	**1**	**2**	**3**	**4**	**5**	
**Visit window (days)**	**N/A**	**N/A**	**±3**	**±4**	**+3**	**+4**
**Type of visit**[k]	**Clinic or off-site**					**Telephone/Telemedicine**
Informed consent	**X**				**X**[c]	
Inclusion/exclusion criteria	**X**	**X**				
Medical history and demographics	**X**					
Confirm documented Rett diagnosis and *MECP2* mutation	**X**					
Rett syndrome history	**X**					
Rett Syndrome Clinical Severity Scale	**X**					
Physical examination[k]	**X**	**X**	**X**	**X**	**X**	
Vital signs[d]	**X**	**X**	**X**	**X**	**X**	
Height	**X**				**X**	
Weight	**X**	**X**	**X**[k]	**X**[k]	**X**[k]	
12-lead ECG[e]	**X**	**X**[e]	**X**	**X**	**X**	
Clinical laboratory tests (hematology, chemistry)	**X**	**X**	**X**	**X**	**X**	
Urinalysis	**X**	**X**	**X**		**X**	
TSH, Free T3, Free T4	**X**	**X**			**X**	
HbA_{1c}	**X**					
Serum pregnancy test[f]	**X**		**X**	**X**	**X**	
Blood samples for pharmacokinetics		**X**[g]	**X**[h]	**X**[h]	**X**[h]	
Blood sample for optional analysis for biomarkers[i]		**X**			**X**	
Rett Syndrome Behaviour Questionnaire (RSBQ)	**X**	**X**	**X**	**X**	**X**	
Clinical Global Impression–Improvement (CGI-I)			**X**	**X**	**X**	
Clinical Global Impression–Severity (CGI-S)	**X**	**X**	**X**	**X**	**X**	
Communication and Symbolic Behavior Scales-Developmental		**X**	**X**	**X**	**X**	

Profile™ Infant-Toddler (CSBS-DP-IT) Checklist						
Rett Syndrome Clinician Rating of Hand Function (RTT-HF)		**X**	**X**	**X**	**X**	
Rett Syndrome Clinician Rating of Ability to Communicate Choices (RTT-COMC)[k]		**X**	**X**	**X**	**X**	
Rett Syndrome Clinician Rating of Ambulation and Gross Motor Skills (RTT-AMB)		**X**	**X**	**X**	**X**	
Rett Syndrome Clinician Rating of Verbal Communication (RTT-VCOM)		**X**	**X**	**X**	**X**	
Rett Syndrome Caregiver Burden Inventory (RTT-CBI)		**X**			**X**	
Impact of Childhood Neurologic Disability Scale (ICND)		**X**			**X**	
Dispensing and review of semi-structured caregiver diary	**X**	**X**	**X**	**X**	**X**	
Concomitant medications	**X**	**X**	**X**	**X**	**X**	**X**
Assessment of adverse events	**X**	**X**	**X**	**X**	**X**	**X**
Randomization		**X**				
Study drug dispensed[j]		**X**				
Authorization of study drug dispensation[j]		**X…………………**				
Study drug return[j]			**X……………..**			
Study drug accountability[j]			**X**	**X**	**X**	

ECG=electrocardiogram; EOT=end of treatment; ET=early termination; HbA1c=glycosylated hemoglobin; *MECP2*=methyl-CpG-binding protein 2 gene; TSH=thyroid stimulating hormone

[a]Timing of postbaseline visits was calculated from the first day of dosing (Day 1) (i.e. the Week 2 visit occurred 2 weeks [±3 days] after the first day of dosing).

[b]Participants who rolled over into the open-label extension (OLE) study did not have the safety follow-up telephone call.

[c]For participants who decided to continue into the OLE study, informed consent for the OLE was obtained prior to performing the Week 12/EOT procedures.

[d]Vital signs included body temperature, resting respiration rate, sitting systolic and diastolic blood pressure, and pulse rate. The sitting blood pressure was measured after the participant had been sitting for ≥3 minutes.

[e]ECGs were completed in triplicate at Visit 1 (Screening), at Visit 2 (Baseline) both before dosing and 2 to 3 hours after dosing, and at Visit 5 (Week 12/EOT/ET). A single ECG was completed at Visit 3 (Week 2) and Visit 4 (Week 6).

[f]For participants who reached menarche and had not had surgical sterilization

[g]A predose pharmacokinetic (PK) blood sample was collected before administration of study drug. A postdose PK blood sample was collected at the end of ECG assessment 2 to 3 hours after study drug administration

[h]PK samples at Visits 3, 4, and 5 were collected at one of the following time intervals: 1) 2 to 3 hours after dosing or 2) 4 to 6 hours after dosing or 3) 7 to 11 hours after dosing. Every effort should have been made to collect the PK samples at discrete time intervals during Visits 3, 4, and 5. However, if the interval was the same across these visits, then the collection time should have varied within that interval

[i]Participation in the effort to identify biomarkers was an optional component of the study requiring a separate informed consent, which may have been obtained at any time during the study. If consent was obtained after Baseline, only the sample at Visit 5 (or upon early termination) was taken.

[j]Investigational product was shipped directly to the participant. Confirmation of any delivery to the participant was made by a visiting nurse. Study drug shipment, return, and accountability were performed in accordance with the drug distribution plan. In addition, study drug was dispensed at the site during the Baseline visit when the visit was conducted in the clinic

[k]Study visits may have been done off-site rather than in the clinic with the prior approval of the Sponsor or Medical Monitor. Screening, Baseline, and EOT visits should have been done in the clinic whenever possible. When a study visit took place off-site, the physical examination was not be required. Weight was measured whenever possible at off-site visits. The RTT-COMC was to be completed, if possible, but it was not required.
