# Supplementary material for: Trofinetide for the treatment of Rett syndrome: a randomized phase 3 study
Source: Nat Med. 2023 Jun 8;29(6):1468–75. doi: 10.1038/s41591-023-02398-1 (PMC10287558; doi:10.1038/s41591-023-02398-1)
Supplement: Supplementary file 2 — Reporting Summary [file 41591_2023_2398_MOESM2_ESM.pdf]

nature portfolio

Corresponding author(s): James M Youakim

Last updated by author(s): Apr 25, 2023

Reporting Summary

Nature Portfolio wishes to improve the reproducibility of the work that we publish. This form provides structure for consistency and transparency in reporting. For further information on Nature Portfolio policies, see our Editorial Policies and the Editorial Policy Checklist.

Statistics

For all statistical analyses, confirm that the following items are present in the figure legend, table legend, main text, or Methods section.

n/a	Confirmed	
☐	☒	The exact sample size (*n*) for each experimental group/condition, given as a discrete number and unit of measurement
☐	☒	A statement on whether measurements were taken from distinct samples or whether the same sample was measured repeatedly
☐	☒	The statistical test(s) used AND whether they are one- or two-sided<br>*Only common tests should be described solely by name; describe more complex techniques in the Methods section.*
☐	☒	A description of all covariates tested
☐	☒	A description of any assumptions or corrections, such as tests of normality and adjustment for multiple comparisons
☐	☒	A full description of the statistical parameters including central tendency (e.g. means) or other basic estimates (e.g. regression coefficient) AND variation (e.g. standard deviation) or associated estimates of uncertainty (e.g. confidence intervals)
☐	☒	For null hypothesis testing, the test statistic (e.g. *F*, *t*, *r*) with confidence intervals, effect sizes, degrees of freedom and *P* value noted<br>*Give P values as exact values whenever suitable.*
☒	☐	For Bayesian analysis, information on the choice of priors and Markov chain Monte Carlo settings
☐	☒	For hierarchical and complex designs, identification of the appropriate level for tests and full reporting of outcomes
☐	☒	Estimates of effect sizes (e.g. Cohen's *d*, Pearson's *r*), indicating how they were calculated

Our web collection on statistics for biologists contains articles on many of the points above.

Software and code

Policy information about availability of computer code

Data collection	No software was used
Data analysis	All statistical analyses were implemented using SAS® software, version 9.4

For manuscripts utilizing custom algorithms or software that are central to the research but not yet described in published literature, software must be made available to editors and reviewers. We strongly encourage code deposition in a community repository (e.g. GitHub). See the Nature Portfolio guidelines for submitting code & software for further information.

Data

Policy information about availability of data

All manuscripts must include a data availability statement. This statement should provide the following information, where applicable:

- Accession codes, unique identifiers, or web links for publicly available datasets
- A description of any restrictions on data availability
- For clinical datasets or third party data, please ensure that the statement adheres to our policy

This clinical trial was sponsored by Acadia Pharmaceuticals Inc. Acadia supports data sharing consistent with the Principles for Responsible Clinical Trial Data Sharing and International Committee of Medical Journal Editors' (ICMJE) Recommendations. Acadia shares data from completed clinical trials through public registries (clinicaltrials.gov), presentation at scientific congresses, and through open access in peer-reviewed journals. Clinical study results from this study will be submitted to clinicaltrials.gov in April 2023. Additional, related information necessary to appraise the quality and robustness of the findings (study protocol, statistical analysis

plan) will be available through supplemental material. The authors will provide access to individual deidentified participant-level data that underlie the data presented in this paper, including data dictionaries, the study protocol and other relevant information, to any researcher who provides a methodologically sound proposal for academic purposes to interpret, verify and extend research in the article beginning 6months and ending 5years after article publication. Requests for the 'minimum dataset' should go through Acadia Medical Information and will be reviewed by the sponsor (Acadia) to verify whether the request is subject to any intellectual property or confidentiality obligations. For additional information, please contact Acadia Medical Information at medicalinformation@acadia-pharm.com.

Human research participants

Policy information about studies involving human research participants and Sex and Gender in Research.

Reporting on sex and gender	The study included only female subjects. The vast majority of people with Rett syndrome are female.
Population characteristics	Females aged 5 to 20 years, with documented MECP2 mutation and diagnosis of classic/typical Rett syndrome with at least 6 months post-regression at screening
Recruitment	Subjects in this study were recruited from 21 sites scattered across the United States. Subjects from 39 states were enrolled. All sites were located at or affiliated with academic medical centers. Investigators recruited families from their clinical databases, through a centralized website referral campaign, through postings on clinicaltrials.gov, and through referrals from various advocacy websites. To promote equitable opportunity to participate in the trial, logistical, travel, accommodation, and financial support were offered to families to help eliminate socioeconomic barriers to participation. Children aged less than 5 years were excluded due to the variable early developmental regression in this age range; older adults (> 20 years) were excluded due to the challenge of controlling for wide discrepancies in services available.
Ethics oversight	The study was conducted in compliance with guidelines from the International Council for Harmonisation (Good Clinical Practice), the Declaration of Helsinki, and the Health Insurance Portability and Accountability Act. The protocol was approved by central (WCG IRB) and local institutional review boards. Prior to screening, informed consent was obtained from the parent or guardian on behalf of the participant.

Note that full information on the approval of the study protocol must also be provided in the manuscript.

Field-specific reporting

Please select the one below that is the best fit for your research. If you are not sure, read the appropriate sections before making your selection.

☒ Life sciences ☐ Behavioural & social sciences ☐ Ecological, evolutionary & environmental sciences

For a reference copy of the document with all sections, see nature.com/documents/nr-reporting-summary-flat.pdf

Life sciences study design

All studies must disclose on these points even when the disclosure is negative.

Sample size	The sample size calculation was performed for the co-primary endpoints as a family of two hypothesis tests at an overall two-sided significance level of 0.05. A total sample size of 174 subjects in a 1:1 ratio to trofinetide or placebo was estimated to provide at least 90% power for the hypothesis testing family assuming the following treatment differences (SD) estimated from Phase 2 study data: -4.4 (8) for the mean change from Baseline to Week 12 in the RSBQ total score and -0.5 (0.7) for the CGI-I mean score at Week 12.<br>The sample size of 174 subjects will provide at least 95% power at a two-sided significance level of 0.05 for each individual hypothesis test within the family. Trofinetide will be claimed to be superior to placebo if both hypothesis tests within the family are shown to be statistically significant at 0.05. Therefore, the overall power to detect a treatment difference on both of the co primary endpoints will be at least 90% (0.952).<br>Adjusting for an anticipated discontinuation rate of up to 5%, approximately 184 subjects will be randomized in a 1:1 ratio to trofinetide or placebo.
Data exclusions	Data analysis sets were predefined and included the Safety Analysis Set (N=187, trofinetide n=93, placebo n=94) and Full Analysis Set (N=184; trofinetide n=91, placebo n=93) as defined in the protocol and the statistical analysis plan.
Replication	Sensitivity analyses including those to account for missing data were performed in addition to the primary analysis. Results for the coprimary endpoints were similar between the primary analysis and the sensitivity analyses.
Randomization	Study participants were stratified by age (5–10, 11–15, and 16–20 years) and baseline RSBQ severity (<35 and ≥35 total score) and randomized 1:1 to trofinetide or placebo using an interactive response technology system via a pre-generated permuted-block randomization schedule.
Blinding	The sponsor, participants, caregivers, and clinicians were blinded to treatment assignment. Participants were assigned to trofinetide or matching placebo.

Reporting for specific materials, systems and methods

We require information from authors about some types of materials, experimental systems and methods used in many studies. Here, indicate whether each material, system or method listed is relevant to your study. If you are not sure if a list item applies to your research, read the appropriate section before selecting a response.

Materials & experimental systems

n/a	Involved in the study
☒	☐ Antibodies
☒	☐ Eukaryotic cell lines
☒	☐ Palaeontology and archaeology
☒	☐ Animals and other organisms
☐	☒ Clinical data
☒	☐ Dual use research of concern

Methods

n/a	Involved in the study
☒	☐ ChIP-seq
☒	☐ Flow cytometry
☒	☐ MRI-based neuroimaging

Clinical data

Policy information about clinical studies

All manuscripts should comply with the ICMJE guidelines for publication of clinical research and a completed CONSORT checklist must be included with all submissions.

Clinical trial registration

ClinicalTrials.gov Identifier: NCT04181723

Study protocol

The study protocol is available and will be uploaded to the Protocol Exchange.

Data collection

All study specific information obtained at each study visit were recorded in the subject's record (source documentation) and then entered into a validated electronic data capture (EDC) database by trained site personnel. The source documentation consisted of source notes captured by site personnel and the caregiver diaries, as well as laboratory reports, ECG reports, and electronic source data. Subject data required by this study were collected at the academic medical center/study site and recorded in an EDC system on electronic case report forms (eCRFs). The Investigator and his or her site personnel were responsible for completing the eCRFs. Subjects were recruited between October 29, 2019, and October 28, 2021.

Outcomes

The two coprimary endpoints were the change from baseline to week 12 in Rett Syndrome Behaviour Questionnaire (RSBQ) total score and the Clinical Global Impression - Improvement (CGI-I) scale score at week 12. The key secondary endpoint was the change from baseline to week 12 in the Communication and Symbolic Behavior Scales Developmental Profile™ Infant-Toddler (CSBS-DP-IT) Checklist – Social Composite Score. The coprimary and key secondary efficacy assessments were completed at baseline (except CGI-I scale) and each visit (weeks 2, 6, and 12 [or end of treatment]).

The caregiver-rated RSBQ consists of 45 items—rated as 0 = "not true," 1 = "somewhat or sometimes true," or 2 = "very true"—that can be grouped into eight symptom domain subscales graded on a scale of 0–90 (maximum severity). The clinician-rated CGI-I scale score uses a Likert scale (1 = very much improved to 7 = very much worse). The CSBS-DP-IT Social Composite score consists of 13 caregiver-rated items, each scored 0 = "not yet," 1 = "sometimes," or 2 = "often" and ranges from 0 to 26 (an increasing score indicates better ability to communicate).

No changes to trial outcomes were made after the trial commenced.
