# Supplementary material for: Trofinetide for the treatment of Rett syndrome: a randomized phase 3 study
Source: Nat Med. 2023 Jun 8;29(6):1468–75. doi: 10.1038/s41591-023-02398-1 (PMC10287558; doi:10.1038/s41591-023-02398-1)
Supplement: Supplementary file 3 — Final version of the statistical analysis plan. [file 41591_2023_2398_MOESM3_ESM.pdf]

STATISTICAL ANALYSIS PLAN

**Protocol No.:**	ACP-2566-003
**Protocol Title:**	A Randomized, Double-Blind, Placebo-Controlled, Parallel-Group Study of Trofinetide for the Treatment of Girls and Women with Rett Syndrome
**Drug:**	trofinetide oral solution
**Sponsor:**	Acadia Pharmaceuticals Inc.
**Version No. and Date**	Version 3.0, 20 October 2021

SIGNATURE/APPROVAL PAGE

AUTHORS	
[redacted]	[redacted]

APPROVERS	
[redacted]	[redacted]
[redacted]	[redacted]

TABLE OF CONTENTS

LIST OF TABLES 6

LIST OF FIGURES 6

ABBREVIATIONS 7

1. INTRODUCTION 9

2. OBJECTIVES 10

2.1 Primary Objective 10
2.2 Key Secondary Objective 10
2.3 Other Secondary Objectives 10
2.4 Safety Objective 10
2.5 Pharmacokinetic Objectives 11

3. STUDY DESIGN 12

3.1 General Study Design 12
3.2 Schedule of Assessments 13
3.3 Randomization 13
3.4 Blinding 13
3.5 Determination of Sample Size 13
3.6 Coronavirus Disease 2019 14

4. ANALYSIS SETS 15

5. DATA HANDLING CONVENTIONS 16

5.1 General Data Reporting Conventions 16
5.2 Derived Efficacy Variables 16
5.2.1 Rett Syndrome Behaviour Questionnaire (RSBQ) 17
5.2.2 Clinical Global Impression–Improvement (CGI-I) 19
5.2.3 Communication and Symbolic Behavior Scales Developmental Profile™ Infant-Toddler Checklist – Social Composite Score (CSBS-DP-IT Social) 19
5.2.4 Overall Quality of Life Rating of the Impact of Childhood Neurologic Disability (ICND) Scale 19
5.2.5 Rett Syndrome Clinician Rating of Hand Function (RTT-HF) 19
5.2.6 Rett Syndrome Clinician Rating of Ambulation and Gross Motor Skills (RTT-AMB) 20
5.2.7 Rett Syndrome Clinician Rating of Ability to Communicate Choices (RTT-COMC) 20

5.2.8 Rett Syndrome Clinician Rating of Verbal Communication (RTT-VCOM) 20
5.2.9 Clinical Global Impression–Severity (CGI-S) 20
5.2.10 Rett Syndrome Caregiver Burden Inventory (RTT-CBI) 20
5.2.11 Impact of Childhood Neurologic Disability (ICND) Scale Total Score 21
5.3 Study Day 21
5.4 Baseline Definition 21
5.5 Analysis Visit Windows 22
5.5.1 Unscheduled Assessments 22
5.5.2 Multiple Measurements within Visit Windows 22
5.6 Missing or Incomplete Date for Last Dose of Study Drug 22
5.7 Missing or Incomplete Dates for Prior or Concomitant Medications 23
5.8 Missing or Incomplete Date for Adverse Events 23
5.9 Missing Severity Assessment for Adverse Events 23
5.10 Missing Relationship to Study Drug for Adverse Events 23
5.11 Character Values of Clinical Laboratory Variables 23

6. SUBJECT DISPOSITION 24

7. PROTOCOL DEVIATIONS 25

8. DEMOGRAPHICS AND BASELINE CHARACTERISTICS 26

9. MEDICAL HISTORY 27

10. EXTENT OF EXPOSURE AND TREATMENT COMPLIANCE 28
10.1 Exposure to Study drug 28
10.2 Measurement of Treatment Compliance 28

11. PRIOR, CONCOMITANT, AND POST-TREATMENT MEDICATION 30

12. EFFICACY ANALYSES 31
12.1 Efficacy Variables 31
12.2 Adjustment for Covariates 31
12.3 Handling of Missing Data 32
12.4 Multiple Comparisons / Multiplicity 32
12.5 Examination of Subgroups 32
12.5.1 *MECP2* Mutation Groupings 33

13. METHODS OF EFFICACY ANALYSES 34
13.1 Primary Efficacy Endpoints 34
13.1.1 Primary Analysis 34

13.1.2 Sensitivity Analyses ... 36
13.1.3 Supportive Analysis ... 40
13.2 Key Secondary Efficacy Endpoints ... 40
13.3 Other Secondary Efficacy Endpoints ... 40
13.4 Exploratory Efficacy Analyses ... 41
13.4.1 CGI-I Responder Analysis ... 41
13.4.2 Hypothetical Strategy Estimand ... 41

14. SAFETY ANALYSES ... 42
14.1 Adverse Events ... 42
14.2 Clinical Laboratory Variables ... 43
14.3 Vital Signs ... 47
14.4 Electrocardiogram (ECG) ... 48
14.5 Physical Examination ... 50
14.6 Other Safety Endpoints ... 50

15. CLINICAL PHARMACOKINETIC AND PHARMACODYNAMIC ANALYSES ... 51

16. INTERIM ANALYSIS ... 52

17. DATA MONITORING/REVIEW COMMITTEE ... 53

18. COMPUTER METHODS ... 54

19. CHANGES TO ANALYSES SPECIFIED IN PROTOCOL ... 55

20. REFERENCES ... 56

21. APPENDICES ... 57
21.1 Schedule of Assessments ... 57
21.2 Summary of Version Changes ... 60

LIST OF TABLES

Table 1 Dosing Schedule Based on Weight at Baseline 12
Table 2 Analysis Visit Windows 22
Table 3 Dosing Schedule and Total Expected Volume Based on Weight at Baseline 28
Table 4 Conversion of Height of Remaining Liquid (cm) into Volume (mL) 29
Table 5 Imputation Predictors 38
Table 6 Criteria for Potentially Clinically Important Laboratory Values – Hematology and Chemistry 45
Table 7 Criteria for Potentially Clinically Important Laboratory Values - Urinalysis 46
Table 8 Criteria for Potentially Clinically Important Vital Signs 48
Table 9 Criteria for Potentially Clinically Important ECG Values 50

LIST OF FIGURES

Figure 1 Schematic of Study Design 14

ABBREVIATIONS

AE	adverse event
ANCOVA	analysis of covariance
ATC	Anatomical/Therapeutic/Chemical
BMI	body mass index
CIs	confidence intervals
CGI-I	Clinical Global Impression – Improvement
CGI-S	Clinical Global Impression – Severity
COVID-19	coronavirus disease 2019
DSMB	Data and Safety Monitoring Board
eCRF	electronic case report form
ECG	Electrocardiogram
EDC	electronic data capture
FAS	Full Analysis Set
FDA	Food and Drug Administration
GJ	Gastrojejunal
GSD	Guidance for Site Documentation and Data Management Querying of Data Impacted by COVID-19
ICND	Impact of Childhood Neurologic Disability Scale
IRT	interactive response technology
MedDRA	Medical Dictionary for Regulatory Activities
MMRM	mixed model for repeated measures
OC	observed cases
PCI	potentially clinically important
PD	pharmacodynamic(s)
PHE	public health emergency
PK	pharmacokinetic(s)
PP	Per Protocol
QTcB	QT Interval Corrected for Heart Rate using Bazett’s Formula
QTcF	QT Interval Corrected for Heart Rate using Fridericia’s Formula

RSBQ	Rett Syndrome Behaviour Questionnaire
RTT	Rett Syndrome
RTT-AMB	Rett Syndrome Clinician Rating of Ambulation and Gross Motor Skills
RTT-CBI	Rett Syndrome Caregiver Burden Inventory
RTT-COMC	Rett Syndrome Clinician Rating of Ability to Communicate Choices
RTT-CSS	Rett Syndrome Clinical Severity Scale
RTT-HF	Rett Syndrome Clinician Rating of Hand Function
RTT-VCOM	Rett Syndrome Clinician Rating of Verbal Communication
SAE	serious adverse event
SAP	statistical analysis plan
SD	standard deviation
SE	standard error
SOC	system organ class
TEAE	treatment-emergent adverse event

1. INTRODUCTION

This statistical analysis plan (SAP) provides a technical and detailed elaboration of the statistical analyses of efficacy and safety data as described in the protocol amendment 2, dated 07 August 2020.

Specifications for tables, figures, and listings are contained in a separate document. Statistical analyses for population pharmacokinetic (PK) and PK/pharmacodynamics (PD) modeling will be presented in a separate report and therefore will not be included in this SAP.

This plan should be read in conjunction with the study protocol and the electronic case report forms (eCRFs).

2. OBJECTIVES

2.1 Primary Objective

The primary objective of this study is to investigate the efficacy of treatment with oral trofinetide versus placebo in girls and women with Rett syndrome.

2.2 Key Secondary Objective

The key secondary objective is to investigate the efficacy of treatment with oral trofinetide versus placebo on ability to communicate in girls and women with Rett syndrome.

2.3 Other Secondary Objectives

Additional secondary objectives for this study are:

- To investigate the benefit of treatment with oral trofinetide versus placebo on overall quality of life for girls and women with Rett syndrome
- To investigate the efficacy of treatment with oral trofinetide versus placebo in girls and women with Rett syndrome on:
    - Hand function
    - Ambulation and other gross motor skills
    - Ability to communicate choices and preferences
    - Ability to communicate verbally
- To investigate the efficacy of treatment with oral trofinetide versus placebo on a global assessment of the severity of illness in girls and women with Rett syndrome
- To investigate the benefit of treatment with oral trofinetide versus placebo on the burden on caregivers of girls and women with Rett syndrome
- To investigate the benefit of treatment with oral trofinetide versus placebo on the impact of the disability on the child's and family's everyday life

2.4 Safety Objective

The safety objective of this study is to investigate the safety and tolerability of treatment with oral trofinetide versus placebo in girls and women with Rett syndrome.

2.5 Pharmacokinetic Objectives

The pharmacokinetic objectives of this study are:

- To characterize the pharmacokinetics (PK) of trofinetide in girls and women with Rett syndrome
- To assess the pharmacokinetic/pharmacodynamic (PK/PD) relationship using safety and efficacy endpoints in girls and women with Rett syndrome

3. STUDY DESIGN

3.1 General Study Design

This study is a Phase 3, multicenter, randomized, double-blind, placebo-controlled, parallel-group study in girls and women with Rett syndrome.

The study drug is oral trofinetide or the matching placebo. Dose will be based on the subject's weight at Baseline, as outlined in Table 1. Doses may be administered by gastrostomy tube (doses administered via gastrojejunal [GJ] tubes must be administered through the G-port).

Table 1 Dosing Schedule Based on Weight at Baseline

Weight	Dose	Total Daily Dose
12-20 kg	30 mL (6 g) BID	60 mL (12 g)
>20-35 kg	40 mL (8 g) BID	80 mL (16 g)
>35-50 kg	50 mL (10 g) BID	100 mL (20 g)
>50 kg	60 mL (12 g) BID	120 mL (24 g)

Abbreviations: BID=twice daily

Subjects will be stratified according to age stratum (5-10 years old, 11-15 years old, and 16-20 years old) and Baseline Rett Syndrome Behaviour Questionnaire (RSBQ) severity (<35 total score and ≥35 total score). A minimum of 12 subjects are required to be randomized for each age stratum. The Sponsor, subjects, caregivers, and Investigators will be blinded to treatment assignment.

The duration of participation for individual study subjects will be approximately 19 weeks, consisting of a screening period of up to 3 weeks, a 12-week double-blind treatment period, and a safety follow-up period of 30 days (for subjects who do not continue into the open-label extension study). The study completion date is defined as the date the final subject, across all sites, completes their final protocol-defined assessment.

Approximately 28 sites will participate in this study. Approximately 184 subjects will be randomized (with a minimum of 12 subjects randomized for three age ranges [5-10 years old, 11-15 years old, and 16-20 years old]) with a total of 92 subjects per treatment arm.

The study design is summarized in Figure 1.

Figure 1 Schematic of Study Design

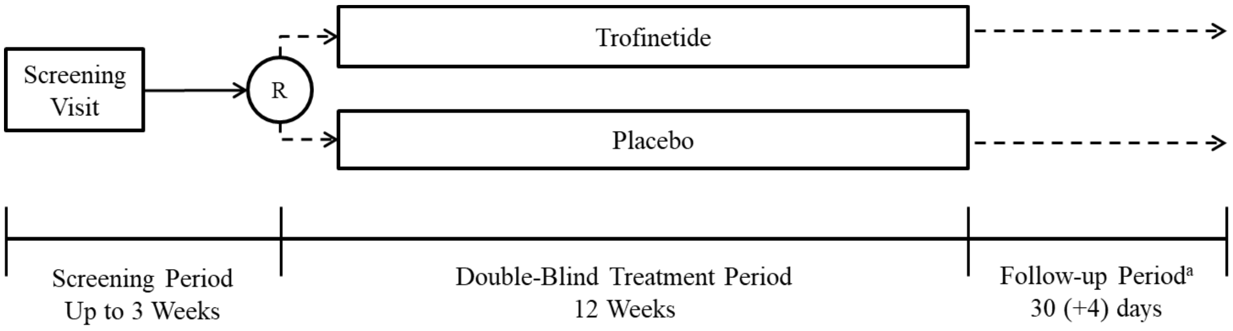

R=Randomization

[a] If the subject continues into the open label extension (OLE) from the current study, she will not complete the follow-up visit and will roll over into the OLE.

3.2 Schedule of Assessments

The schedule of assessments is in the Appendix (Section 21.1).

3.3 Randomization

Eligible subjects will be randomized into one of two treatment groups (trofinetide or placebo) in a 1:1 ratio using an interactive response technology (IRT) system. The randomization will be stratified by age group (5-10 years old, 11-15 years old, and 16-20 years old) and Baseline RSBQ severity (<35 total score and ≥35 total score). The assignments will be based on a pre-generated permuted-block randomization schedule.

3.4 Blinding

This is a double-blind study. Blinding will be assured by restricting access of Investigators and Sponsor personnel and/or designee to the treatment codes, and providing identical packaging for the trofinetide and placebo treatments.

Unblinding of individual treatment assignment during the study is discouraged. The Investigator may break the blind in the event of a medical emergency if it is considered necessary for the care of the subject. The Investigator should attempt whenever possible to contact the Medical Monitor before unblinding a subject's treatment to discuss the event.

For the final analysis, the treatment codes for all subjects will be released to Acadia after all subjects have completed the study and the clinical database is locked.

3.5 Determination of Sample Size

The sample size calculation was performed for the co-primary efficacy endpoints as a family of two hypothesis tests at an overall two-sided significance level of 0.05. A total sample size of 174 subjects in a 1:1 ratio to trofinetide or placebo was estimated to provide at least 90%

power for the hypothesis testing family assuming the following treatment differences (SD) estimated from Phase 2 study data: -4.4 (8) for the mean change from Baseline to Week 12 in the RSBQ total score and -0.5 (0.7) for the CGI-I mean score at Week 12.

The sample size of 174 subjects will provide at least 95% power at a two-sided significance level of 0.05 for each individual hypothesis test within the family. Trofinetide will be claimed to be superior to placebo if both hypothesis tests within the family are shown to be statistically significant at 0.05. The overall power to detect a treatment difference on both of the co-primary efficacy endpoints would be at least 90% (0.95^2).

Adjusting for an anticipated discontinuation rate of up to 5%, approximately 184 subjects will be randomized in a 1:1 ratio to trofinetide or placebo to ensure 174 subjects who complete the study.

3.6 Coronavirus Disease 2019

In March 2020, the emerging coronavirus disease 2019 (COVID-19) pandemic resulted in implementation of urgent safety measures designed to ensure subject safety. Mechanisms to record information on the potential impact of the COVID-19 pandemic on data itself, as well as data collection and integrity, were implemented (as detailed in the appendix of ACP-2566-003's Data Management Plan titled "Guidance for Site Documentation and Data Management Querying of Data Impacted by COVID-19" [GSD] included in eTMF).

Relationship to the public health emergency caused by the COVID-19 pandemic will be assessed for early terminations, protocol deviations, selected medications, and selected adverse events as detailed in the GSD.

Subjects who were discontinued from Study ACP-2566-003 before completion because of the COVID-19 public health emergency and subjects who completed Study ACP-2566-003, but were prevented from entering Study ACP-2566-004 because of the COVID-19 public health emergency, may be re-evaluated for eligibility to enroll into the open-label extension study (Study ACP-2566-004).

To assess the impact of COVID-19 PHE on the study population and data collection, summaries of demographics, baseline characteristics and Rett syndrome history by randomization date before or after the onset of COVID-19 (18 March 2020) and COVID-19 PHE related intercurrent events will be summarized.

4. ANALYSIS SETS

Randomized Analysis Set

The Randomized Analysis Set consists of all subjects who were randomized. Subjects will be classified according to the randomized treatment assignment.

Safety Analysis Set

The Safety Analysis Set consists of all randomized subjects who received at least one dose of study drug. The Safety Analysis Set will be analyzed according to the actual treatment received.

Full Analysis Set

The Full Analysis Set (FAS) consists of all subjects who were randomized, received at least one dose of study drug, and have both a Baseline value and at least one post-Baseline value for the RSBQ total score or have at least one CGI-I score after taking study medication. The FAS will be analyzed according to the treatment they were randomized regardless of the actual treatment received.

Per-protocol Analysis Set

The Per-protocol Analysis Set consists of the subjects in the Full Analysis Set who do not have a major protocol violation that would affect interpretation of the efficacy data. The Per-protocol Analysis Set will be defined prior to study unblinding. The Per-protocol Analysis Set will be analyzed according to randomized treatment assignment.

Pharmacokinetics (PK) Analysis Set

The PK Analysis Set consists of the subjects in Safety Analysis Set with at least one measurable trofinetide whole blood concentration. Subjects will be classified according to the actual treatment received.

5. DATA HANDLING CONVENTIONS

All data collected in the study will be listed.

5.1 General Data Reporting Conventions

Continuous variables will be summarized using the following descriptive statistics: number of subjects, mean, median, standard deviation (SD), standard error (SE), minimum, and maximum. Unless specified otherwise, means, medians, and confidence intervals (CIs) will be presented to one more decimal place than the raw data, and the standard deviations and standard errors will be presented to two more decimal places than the raw data. In general, the maximum number of decimal places is 4 and values will be truncated to 4 decimal places in situations where there are more than 4 decimal places. Wherever possible data will be decimal aligned.

Height, weight and BMI will be presented with a maximum of one decimal place.

Categorical variables will be summarized by the number of subjects and the percent of subjects in each category; the number of subjects and the percentage of subjects with missing data will be summarized for demographic and baseline characteristics (if applicable). Categories with zero counts will not have zero percentages displayed. For demographic summaries, percentages will be calculated by using the total number of subjects in the given treatment group as the denominator. Percentages will be presented with one decimal place.

Duration in months will be calculated as ([the number of days / 365.25] *12).

Unless specified otherwise, all statistical tests will be 2-sided hypothesis tests performed at the significance level of 5% for main effects and all CIs will be 2-sided 95% CIs. P-values will generally be presented to 4 decimal places; values less than 0.0001 will be presented as <0.0001.

Clinical laboratory assessment values that are collected with “<” or “>” signs will generally be analyzed as the numerical value without the sign in tables and figures. In listings, these data will be reported as collected with the sign.

5.2 Derived Efficacy Variables

In general, assessment total scores and subscores will be derived within the analysis datasets. In the event that total scores and/or subscores are also collected on the electronic case report from (eCRF), the derived values will be used for all analyses. Both the raw and derived scores will be presented in listings.

5.2.1 Rett Syndrome Behaviour Questionnaire (RSBQ)

The RSBQ is a 45-item caregiver-completed rating scale in which the caregiver rates items as "0" (Not True), "1" (somewhat or sometimes true) or "2" (very true). The RSBQ includes 8 subscales:1) general mood, 2) breathing problems, 3) hand behavior, 4) face movements, 5) body rocking/expressionless face, 6) night-time behaviors, 7) fear/anxiety, and 8) walking/standing. The total score ranging from 0 to 90 will be calculated as the sum of the scores for all 45 items. Score for item 31 (Uses eye gaze to convey feelings, needs and wishes.) will be reversed (2-observed item score) in the calculation of total score and subscores. Higher total scores indicate greater frequency of symptoms. If there are 9 or less missing item scores, the total score will be calculated by the arithmetic mean of non-missing items multiplied by 45, rounded to the nearest integer. The RSBQ total score will be considered missing if there are missing scores for 10 or more items.

For the calculation of RSBQ subscores, the items under each RSBQ subscore will be summed. If an item is missing it will not be imputed and the subscore will be considered missing.

<u>RSBQ Subscores</u>

1) General mood consists of:
    a. Abrupt changes in mood (Item 14)
    b. Spells of screaming for no apparent reason during the day (Item 2)
    c. There are times when she appears miserable for no apparent reason (Item 16)
    d. Spells of inconsolable crying for no apparent reason during the day (Item 30)
    e. There are times when she is irritable for no apparent reason (Item 29)
    f. Screams hysterically for long periods of time and cannot be consoled (Item 22)
    g. There are certain days/periods where she performs worse than usual (Item 15)
    h. Vocalises for no apparent reason (Item 36)
2) Breathing problems consists of:
    a. There are times when breath is held (Item 5)
    b. Swallows air (Item 19)
    c. Abdomen fills with air and sometimes feels hard (Item 25)
    d. There are times when breathing is deep and fast (hyperventilation) (Item 1)
    e. Air or saliva expelled from mouth with force (Item 6)
3) Hand behaviors consists of:
    a. Does not use hands for purposeful grasping (Item 18)
    b. Restricted repertoire of hand movement (Item 24)
    c. Hand movements uniform and monotonous (Item 20)

d. The amount of time spent looking at an object is longer than time spent holding or manipulating them (Item 43)
e. Has difficulty in breaking/stopping hand stereotypies (Item 35)
f. Has frequent naps during the day (Item 21)

4) Repetitive face movements consists of:
   a. Makes mouth grimaces (Item 28)
   b. Makes repetitive tongue movements (Item 32)
   c. Makes grimacing expressions with face (Item 34)
   d. Makes repetitive movements involving fingers around tongue (Item 4)
5) Body rocking and expressionless face consists of:
   a. Rocks body repeatedly (Item 41)
   b. Expressionless face (Item 12)
   c. Rocks self when hands are prevented from moving (Item 33)
   d. Seems to look through people in to the distance (Item 17)
   e. Uses eye gaze to convey feelings, needs and wishes (reversed) (Item 31)
   f. Tendency to bring hands together in front of chin or chest (Item 40)
6) Night-time behaviours consists of:
   a. Spells of screaming for no apparent reason during the night (Item 13)
   b. Spells of inconsolable crying for no apparent reason during the night (Item 42)
   c. Spells of laughter for no apparent reason during the night (Item 37)
7) Fear/Anxiety consists of:
   a. Spells of apparent panic (Item 38)
   b. Spells of apparent anxiety/fear in unfamiliar situations (Item 7)
   c. Seems frightened when sudden changes in own body position (Item 9)
   d. There are times when parts of the body are held rigid (Item 10)
8) Walking/standing consists of:
   a. Walks with stiff legs (Item 39)
   b. Although can stand independently tends to lean on objects or people (Item 23)

Items not included in Subscores

a. Makes repetitive hand movements with hands apart (Item 3)
b. Grinds teeth (Item 8)
c. Shifts gaze with a slow horizontal turn of head (Item 11)
d. Spells of laughter for no apparent reason during the day (Item 26)
e. Has wounds on hands as a result of repetitive hand movements (Item 27)
f. Appears isolated (Item 44)
g. Vacant ‘staring’ spells (Item 45)

5.2.2 Clinical Global Impression–Improvement (CGI-I)

The CGI-I scale is used by the clinician to rate how much the subject's illness has improved or worsened relative to a baseline state. A 7-point scale is used from 1=very much improved, 2=much improved, 3=minimally improved, 4=no change, 5=minimally worse, 6=much worse, 7=very much worse.

Higher CGI-I scores denote more severe illness and less improvement in the illness respectively.

5.2.3 Communication and Symbolic Behavior Scales Developmental Profile™ Infant-Toddler Checklist – Social Composite Score (CSBS-DP-IT Social)

The Checklist consists of 24 questions that range from 0 to 4 points within each of 7 Clusters. Credit of 0 points is given for items checked "Not Yet", 1 point for items checked "Sometimes", or 2 points for items checked "Often". For items that describe a series of numbers or ranges, credit of 0 points is given for items checked "None" and 1 to 4 points for items containing numbered choices.

Three composite scores assessing 7 skill areas can be calculated. The Social Composite score is comprised of 13 items in the skill areas "Emotion and Eye Gaze" (items 1 to 4), "Communication" (items 5 to 8), and "Gestures" (items 9 to 13). The Speech Composite score, including the skill areas "Sounds" and "Words" is based on items 14 to 18. The Symbolic Composite score, including the skill areas "Understanding" and "Object Use", is based on items 19 to 24.

The Social Composite raw score (comprised of items 1 to 13) ranging from 0 to 26 will be calculated as the sum of the scores for all 13 items. Higher Social Composite raw scores indicate better social communication development. If there are 2 or less missing item scores, the Social Composite raw score will be calculated by the arithmetic mean of the non-missing item scores multiplied by 13, rounded to the nearest integer. The Social Composite raw score will be considered as missing if there are missing scores for 3 or more items.

5.2.4 Overall Quality of Life Rating of the Impact of Childhood Neurologic Disability (ICND) Scale

The overall quality of life score rating of the ICND will be analyzed as a separate endpoint. The numeric scale of the child's overall quality of life ranges from 1 ("Poor") to 6 ("Excellent"); lower overall quality of life scores indicate lower quality of life.

5.2.5 Rett Syndrome Clinician Rating of Hand Function (RTT-HF)

The RTT-HF is a clinician completed clinical assessment of the subject's ability to use her hands for functional purposes. The assessment is made on an 8-point Likert scale (0-7) with 0 denoting normal functioning and 7 the most severe impairment.

5.2.6 Rett Syndrome Clinician Rating of Ambulation and Gross Motor Skills (RTT-AMB)

The RTT-AMB is a clinician completed clinical assessment of the subject's ability to sit, stand, and ambulate. The assessment is made on an 8-point Likert scale (0-7) with 0 denoting normal functioning and 7 the most severe impairment.

5.2.7 Rett Syndrome Clinician Rating of Ability to Communicate Choices (RTT-COMC)

The RTT-COMC is a clinician completed clinical assessment of the subject's ability to communicate her choices or preferences, which can include the use of nonverbal means such as eye contact or gestures. The assessment is made on an 8-point Likert scale (0-7) with 0 denoting normal functioning and 7 the most severe impairment.

5.2.8 Rett Syndrome Clinician Rating of Verbal Communication (RTT-VCOM)

The RTT-VCOM is a clinician completed clinical assessment of the subject's ability to communicate verbally. The assessment is made on an 8-point Likert scale (0-7) with 0 denoting normal functioning and 7 the most severe impairment.

5.2.9 Clinical Global Impression–Severity (CGI-S)

The CGI-S is a 7-point scale that requires the clinician to rate the severity of the subject's illness at the time of assessment, relative to the clinician's experience with subjects who have the same diagnosis. Considering total clinical experience, a subject is assessed on severity of illness at the time of rating: 1=normal, not at all ill; 2=borderline ill; 3=mildly ill; 4=moderately ill; 5=markedly ill; 6=severely ill; or 7=extremely ill.

Higher CGI-S scores denote more severe illness and less improvement in the illness respectively.

5.2.10 Rett Syndrome Caregiver Burden Inventory (RTT-CBI)

The RTT-CBI consists of 24 negatively worded items (Items 1 through 24). Frequency ratings are on a 5-point Likert scale including: 0-never; 1-rarely; 2-sometimes; 3-frequently and 4-nearly always. The RTT-CBI also includes 2 positively worded items (items 25 and 26) that comprise the Optimism Index; this index will not be used for analysis. The total score ranging from 0 to 96 will be calculated as the sum of the scores for Items 1-24. If there are 4 or fewer missing item scores, the RTT-CBI total score will be calculated by the arithmetic mean of the non-missing item scores multiplied by 24, rounded to the nearest integer. The RTT-CBI total score will be considered as missing if there are missing scores for 5 or more items.

5.2.11 Impact of Childhood Neurologic Disability (ICND) Scale Total Score

The ICND scale evaluates the effect of four health problems on 11 aspects of the child's or the family's life which are scored 0 ("Not at all"), 1 ("A little"), 2 ("Some"), 3 ("A lot"), or "Does not apply". The four health problems are 1) inattentiveness, impulsivity, or mood, 2) ability to think and remember, 3) neurologic or physical limitations, and 4) epilepsy. The ICND total score ranging from 0 to 132 will be calculated as follows:

1. For each health problem, the score will be calculated as the sum of the 11 item scores. The health problem score will be considered missing
    a. if more than 5 item scores are either missing or "Does not apply"
    b. or if more than 3 item scores are missing for reasons other than the response of "Does not apply".
2. The ICND total score will be calculated as the sum of the average of each problem score multiplied by 11.

$$ICND\ Total\ Score = \sum_{k=1}^{4} problem\_score\_average_k * 11$$

where k indexes the four health problems.

The ICND total score will be considered missing if at least 1 health problem score is missing. Imputed ICND total scores will be rounded to the nearest integer.

Higher ICND total scores indicate worse health problems. The ICND total score does not include the Overall Quality of Life Rating.

5.3 Study Day

If the date of assessment occurs on or after the first dose date, then study day will be calculated as (date of assessment – date of first dose) + 1. If the date of assessment occurs prior to the first dose date, then study day will be calculated as (date of assessment – date of first dose). There is no study day 0.

5.4 Baseline Definition

Baseline data are defined as data collected which are prior to the administration of the first dose. If there is more than one value on or prior to Study Day 1, the value closest to and prior to the receipt of the first dose, whether scheduled or unscheduled, will be used as the Baseline value.

5.5 Analysis Visit Windows

Baseline of the study is defined as the last non-missing result, including results from repeated and unscheduled measurements, before first dosing.

Efficacy, safety, and PK assessments will be summarized by analysis visit as presented in Table 2 below.

Table 2 Analysis Visit Windows

Analysis Visit	Target Study Day	Study Day Interval
Baseline (Day 1)	1	≤ 1
Day 1 Post First Dose*	1	1
Week 2	15	2 – 28
Week 6	43	29 – 63
Week 12	85	64 – 115

*For ECG and PK assessments only; other assessments post-first dose on Day 1 will go to the Week 2 analysis visit window.

5.5.1 Unscheduled Assessments

Both Scheduled and Unscheduled assessments, including the assessments at early termination visits, will be included for planned timepoint analyses based on the above analysis visit windowing rules. All assessments will be presented in data listings.

5.5.2 Multiple Measurements within Visit Windows

In the event that more than one assessment falls within a given window, the assessment closest to the target study day will be selected for the by-visit analysis. If two assessments are equidistant from the target study day, then the chronologically last assessment will be used.

For safety analyses where the extreme values should be selected (e.g., overall post-Baseline minimum, overall post-Baseline maximum, and potentially clinically important values), all non-missing post-Baseline values should be considered, regardless of whether the value is selected for the by-visit summaries. All assessments will be presented in data listings.

5.6 Missing or Incomplete Date for Last Dose of Study Drug

For subjects with completely missing last dose date, the last dose date will be imputed by the last expected dosing date, defined as the earliest of the following dates: last drug kit dispense date + scheduled dosing interval per protocol, EOT/ET date and the return date of the last dispensed drug kit.

For subjects with partial missing last dose date, the imputation will be compared against the last expected dosing date as defined above. Detailed algorithms will be documented in a separate programming specifications document. The missing or incomplete dates will be displayed in the data listings as reported on the eCRF rather than the imputed dates.

5.7 Missing or Incomplete Dates for Prior or Concomitant Medications

Missing or incomplete medication start or stop dates will be imputed for the purpose of determining whether the medication is taken concomitantly or not (see Section 11 for definition). When the chronological order of medication use relative to the study drug treatment period is unclear due to missing or incomplete date(s), the medication will be considered as concomitant. The imputation algorithms will be detailed in the analysis dataset specification document. The missing or incomplete dates as captured on the eCRF will be displayed in the data listings.

5.8 Missing or Incomplete Date for Adverse Events

Missing or incomplete adverse event (AE) start dates will be imputed for the purpose of determining whether the AEs are treatment-emergent or not (see Section 14.1 for definition). When the chronological order of an AE onset relative to the study drug treatment period is unclear due to missing or incomplete date(s), the AE will be considered as treatment-emergent. The imputation algorithms will be detailed in the analysis dataset specification document. The missing or incomplete dates captured on the eCRF will be displayed in the data listings.

5.9 Missing Severity Assessment for Adverse Events

If the severity is missing for an AE starting on or after the date of the first dose of study drug, then a severity of “Severe” will be assigned. The imputed values for severity assessment will be used for incidence summaries, while the actual values will be used in data listings.

5.10 Missing Relationship to Study Drug for Adverse Events

If the relationship to study drug is missing for an AE starting on or after the date of the first dose of study drug, a causality of “Related” will be assigned. The imputed values for relationship to study drug will be used for incidence summaries, while the actual values will be presented in data listings.

5.11 Character Values of Clinical Laboratory Variables

If the reported value of a clinical laboratory variable cannot be used in a statistical analysis due to, for example, a character string reported for a numeric variable, an appropriately determined coded value may be used in the statistical analysis. The coding algorithms will be detailed in the analysis dataset specification document. The actual values as reported in the database will be presented in data listings.

6. SUBJECT DISPOSITION

For subjects who participate in the screening phase but are not randomized (screen failures), their demographics information (including age, sex, and primary race), screen failure reasons (the specific inclusion/exclusion criterion (or criteria) not met or other reasons including the reason due to the COVID-19 public health emergency (PHE) and protocol version will be listed. If a subject is re-screened, then the re-screening subject ID and the final enrollment status (whether eventually enrolled) will also be displayed in this listing. In addition, the frequency that the screen failure reasons are reported will be summarized. Note that one subject may be deemed ineligible for multiple inclusion/exclusion criteria and may be allowed to rescreen with the permission of the Medical Monitor, provided the screen failure was due to a temporary condition that subsequently resolved.

The number of sites that screened at least 1 subject, number of sites that randomized at least 1 subject, number of subjects screened, and number of unique subjects screened will be tabulated. In addition, the number of subjects enrolled at each site will also be tabulated by Analysis Set and by treatment group and overall.

The number and percentage of subjects who completed the study, discontinued early (all discontinued and by discontinuation reasons including reason due to the COVID-19 PHE), and the reason for discontinuation will be summarized by treatment group using the Randomized and Full Analysis Sets. All subjects excluded from the Safety, Full or Per-protocol Analysis Sets, and the reason(s) for exclusion will be listed. The number and percentage of subjects who are excluded from the Per-protocol Analysis Set will be presented in a summary table by reason, and by treatment group and overall.

7. PROTOCOL DEVIATIONS

Protocol deviations will be reviewed periodically over the course of the study. The review process, definition of the deviation categories, and the classification of a deviation as major or minor are detailed in the Protocol Deviation Management Plan. Protocol deviations will also be assessed with respect to relationship to the COVID-19 PHE.

A summary of the number and percentage of subjects with major protocol deviations for each deviation category will be presented by treatment group for the Randomized Analysis Set in three ways: all protocol deviations, COVID-19-PHE related protocol deviations, and non-COVID-19-PHE related protocol deviations.

Three data listings of all protocol deviations, COVID-19-PHE related protocol deviations, and non-COVID-19-PHE related protocol deviations will be provided.

8. DEMOGRAPHICS AND BASELINE CHARACTERISTICS

Demographics and baseline characteristics will be summarized by treatment group and overall for Randomized Analysis Set and Full Analysis Set using descriptive statistics. Variables include, but are not limited to, age, age group 1 (5-10 years old, 11-15 years old, and 16-20 years old), age group 2 (5-12 years old, 13-17 years old, and ≥18 years old), race, ethnicity, height, weight, BMI, Baseline RSBQ total score, Baseline RSBQ severity (< 35 total score and ≥ 35 total score), Baseline CGI-S score, and Rett Syndrome Clinical Severity Scale (RTT-CSS) score at Screening.

Race will also be categorized by White vs. Non-White. The reported age reflects a subject's age at the informed consent date.

To assess the impact of COVID-19 PHE on study population, summaries of demographics and baseline characteristics will be presented for subjects randomized before and after the onset of COVID-19 PHE (18 March 2020).

9. MEDICAL HISTORY

Medical history reported terms will be coded with Medical Dictionary for Regulatory Activities (MedDRA), version 23.0 or newer. The subject incidence will be summarized for each system organ class (SOC) and preferred term by treatment group and overall for the Safety Analysis Set. A subject will be counted only once per SOC or per preferred term for the summary.

A listing of the SOC, preferred term, body system, verbatim for the medical history condition/event, start and stop dates (when available), and an indicator for whether or not the condition is ongoing will be provided.

Rett Syndrome History

The age at Rett syndrome diagnosis, the age at first symptoms noticed, the number and percentage of subjects by *MECP2* gene mutation, and the number and percentage of subjects with more than one *MECP2* mutation (Yes, No) collected on the Rett History eCRF page will be summarized.

To assess the impact of COVID-19 PHE on study population, summaries of Rett syndrome history will be presented for subjects randomized before and after the onset of COVID-19 PHE (18 March 2020).

Rett syndrome history will be listed. A flag variable will indicate if a subject was randomized after the onset of COVID-19 PHE.

10. EXTENT OF EXPOSURE AND TREATMENT COMPLIANCE

Extent of exposure and treatment compliance will be summarized as both continuous variables and categorical variables by treatment group for Safety Analysis set.

10.1 Exposure to Study drug

Duration of exposure to study drug will be calculated for each subject as (last dose date – first dose date + 1). The number and percentage of subjects within each of the following exposure levels in terms of duration of exposure will also be tabulated: <2 week (1 to 13 days), 2 to <4 weeks (14 to 27 days), 4 to <6 weeks (28 to 41 days), 6 to <8 weeks (42 to 55 days), 8 to <10 weeks (56 to 69 days), 10 to <12 weeks (70 to 83 days), and >=12 weeks (84 days or longer).

10.2 Measurement of Treatment Compliance

The study drug is provided in liquid form supplied in a 500 mL bottle. Study drug compliance will be calculated based on the drug accountability and dose modification data as collected on the eCRF. The study drug compliance will be calculated as (the total volume of drug actually taken (in mL) divided by the total volume of drug expected to be taken)*100.

The total volume of drug expected to be taken will be based on the duration of exposure and dosing schedule as in Table 3. However, if there is any dose modification prescribed by the investigator due to intolerance, the total drug expected to be taken will be adjusted accordingly to account for the modified prescribed dose schedule as recorded in the EDC.

The total volume of drug actually taken will be calculated as (total drug dispensed – total drug returned (height in cm of drug remaining converted into volume in mL based on the following conversion Table 4).

Treatment compliance will be summarized as a categorical variable. The number and percentage of subjects within each of the following compliance levels will be tabulated: <80%, 80 to 120% and >120%.

Details of treatment compliance calculation are provided in a separate programming specifications document.

Table 3 Dosing Schedule and Total Expected Volume Based on Weight at Baseline

Weight	Dose	Total Daily Dose	Total Expected Volume
12-20 kg	30 mL (6 g) BID	60 mL (12 g)	60 mL x Number of Days Expected to be Dosed
>20-35 kg	40 mL (8 g) BID	80 mL (16 g)	80 mL x Number of Days Expected to be Dosed
>35-50 kg	50 mL (10 g) BID	100 mL (20 g)	100 mL x Number of Days Expected to be Dosed
>50 kg	60 mL (12 g) BID	120 mL (24 g)	120 mL x Number of Days Expected to be Dosed

BID = twice daily

Table 4 Conversion of Height of Remaining Liquid (cm) into Volume (mL)

Height of Remaining Liquid (cm)	Estimated Volume (ml)	Height of Remaining Liquid (cm)	Estimated Volume (ml)
0.5	18	7.0	248
1.0	35	7.5	266
1.5	53	8.0	283
2.0	71	8.5	301
2.5	89	9.0	319
3.0	106	9.5	336
3.5	124	10.0	354
4.0	142	10.5	372
4.5	159	11.0	389
5.0	177	11.5	407
5.5	195	12.0	425
6.0	212	12.5	443
6.5	230	13.0	460

11. PRIOR, CONCOMITANT, AND POST-TREATMENT MEDICATION

Prior medication is defined as any medication with stop dates prior to the date of the first dose of study drug. Concomitant medication is defined as any medications that are ongoing at the first dose of study drug or with a start date between the dates of the first and last doses of study drug, inclusive. Post-treatment medication is defined as any medication with a start date after the date of the last dose of study drug. Medications will be coded using WHO Drug Global Dictionary March 2020 or newer version.

The number and percentage of subjects taking prior, concomitant and post-treatment medications will be tabulated separately by each drug class (ATC Level 3) and medication preferred term, treatment group and overall for Safety Analysis Set. Multiple medication usage by a subject in the same category will be counted only once. Listings of the prior, concomitant and post-treatment medications will also be provided.

COVID-19 Infection Related Medications

Concomitant medication analyses described above will also be tabulated and listed by relationship to COVID-19 Infection (Not related to COVID-19 Infection vs. Related to COVID-19 Infection).

12. EFFICACY ANALYSES

Unless otherwise specified, all efficacy analyses will be performed using the planned treatment assignments based on the randomization schedule for the Full Analysis Set.

12.1 Efficacy Variables

Co-Primary Efficacy Endpoints

The co-primary efficacy endpoints are:

- RSBQ total score – change from Baseline to Week 12
- CGI-I score at Week 12

Key Secondary Efficacy Endpoints

The key secondary endpoint is change from Baseline to Week 12 in CSBS-DP-IT Social Composite Score.

Other Secondary Efficacy Endpoints

The other secondary endpoints for this study are change from Baseline to Week 12 in:

- Overall Quality of Life Rating of the ICND
- RTT-HF score
- RTT-AMB score
- RTT-COMC score
- RTT-VCOM score
- CGI-S score
- RTT-CBI total score (items 1-24)
- ICND total score

12.2 Adjustment for Covariates

The Baseline RSBQ total score will be included as a covariate for the analysis of change from baseline in the RSBQ total score using the mixed model for repeated measures (MMRM). The Baseline CGI-S score will be included as a covariate for the analysis of CGI-I score using the MMRM method. The Baseline CSBS-DP-IT Social Composite Score will be included as a covariate for the analysis of change from Baseline in the CSBS-DP-IT Social Composite Score using the MMRM method. Unless otherwise specified, the randomization stratification variables of age group (5-10 years old, 11-15 years old, and 16-20 years old)

and Baseline RSBQ severity (<35 total score and ≥35 total score) will also be included as fixed effects in the MMRM method.

12.3 Handling of Missing Data

The primary analyses of

- RSBQ total score – change from Baseline to Week 12 (co-primary efficacy endpoint)
- CGI-I score at Week 12 (co-primary efficacy endpoint)
- CSBS-DP-IT Social Composite Score – change from Baseline to Week 12 (key secondary endpoint)

will be performed assuming missing at random (MAR) using the direct likelihood-based MMRMs and missing scores (after any imputation of individual missing items as described in Section 5.2) will not be imputed. The MMRM method is unbiased under the MAR assumptions in the estimation of treatment effect that could have been observed if all subjects had continued on treatment for the full study duration (EMA, 2009).

12.4 Multiple Comparisons / Multiplicity

There are a total of three hypotheses, two primary hypotheses and a key secondary hypothesis, grouped in two families. The two primary hypotheses form one family and the key secondary, the other family. The family of the two primary hypotheses constitute the gatekeeper. Each hypothesis in the gatekeeper will be tested at the two-sided α=5% without multiplicity adjustment because both hypotheses in the family must be rejected to move forward to the next family.

The family of the key secondary hypothesis will be tested if and only if trofinetide is superior to the placebo with respect to both co-primary efficacy endpoints. The key secondary hypothesis will be tested at the two-sided α=5%. This hierarchical testing of the family of primary hypotheses and that of the key secondary hypothesis will provide a strong control of the family-wise error rate at the nominal α=5%.

12.5 Examination of Subgroups

Treatment effect will be examined with respect to the co-primary and key secondary efficacy endpoints within the subgroups of age group 1 (5-10 years old, 11-15 years old, and 16-20 years old) , age group 2 (5-12 years old, 13-17 years old, and ≥18 years old), and Baseline RSBQ severity (<35 total score and ≥35 total score). Subgroup analysis by *MECP2* mutation groupings will also be performed; if a subgroup size is too small, categories may be pooled prior to analysis, and footnoted appropriately.

12.5.1 *MECP2* Mutation Groupings

The number and percentage of subjects will be examined in the following *MECP2* mutation groupings.

Mutations	Code in EDC	Category	Severity
R106W	1	R106W	Severe
R133C	2	R133C	Mild
T158M	3	T158M	Moderate
R168X	4	R168X	Severe
R255X	5	R255X	Severe
R270X	6	R270X	Severe
R294X	7	R294X	Mild
R306C	8	R306C	Mild
C298G (L100V)	9	Other mutations	Mild
G317A (R106Q	10	Other mutations	Mild
C421G (Y141X)	11	Other mutations	Unknown
C455G (P152R)	12	Other mutations	Severe
C302G (P101R)	13	Other mutations	Severe
C401G (S134C)	14	Other mutations	Severe
C423G (Y141X)	15	Other mutations	Unknown
C468G (D156E)	16	Other mutations	Mild
C674G (P225R)	17	Other mutations	Mild
C965T (P322L)	18	Other mutations	Mild
710del1	19	Other mutations	Severe
806del1	20	Other mutations	Severe
807del1	21	Other mutations	Severe
1157del41	22	C-terminal Truncations	Mild
1157del44	23	C-terminal Truncations	Mild
1163del26	24	C-terminal Truncations	Mild
1163del35	25	C-terminal Truncations	Mild
1164del44	26	C-terminal Truncations	Mild
1168del6	27	C-terminal Truncations	Mild
Exon 1+2	28	Large Deletions	Severe
Exon 3	29	Large Deletions	Severe
Exon 3+4	30	Large Deletions	Severe
Exon 4	31	Large Deletions	Severe
Exons 1-4	32	Large Deletions	Severe
All Others	33	Other mutations	Derived from CRF

Missing and unknown severity will not be imputed.

13. METHODS OF EFFICACY ANALYSES

13.1 Primary Efficacy Endpoints

13.1.1 Primary Analysis

Estimand

Target Population: Female subjects 5 to 20 years of age inclusive with Rett syndrome, as defined by the inclusion/exclusion criteria of the study.

Variables: The co-primary endpoints in this study are the change from baseline to Week 12 in the RSBQ total score and the CGI-I score at Week 12.

Intercurrent events: Possible intercurrent events include:

- Treatment Discontinuation not due to COVID-19 PHE
- Treatment Discontinuation due to COVID-19 PHE
- COVID-19 PHE events leading to intermediate missing data
- Non-COVID-19 PHE events leading to intermediate missing data
- Remote Assessments (regardless of due to COVID-19 PHE or not)

Summary measure: Treatment difference in the mean change from Baseline to Week 12 in the RSBQ total score and treatment difference in the mean CGI-I score at Week 12.

In general, the treatment policy strategy will be used to handle all intercurrent events in the primary analysis. That is, observations on the co-primary efficacy endpoints will be used regardless of the occurrence of intercurrent events.

Alternative approaches to handling intercurrent events will be addressed in sensitivity analyses.

Hypotheses

Let Δ_1 ($\boldsymbol{\mu}_{TRO1} - \boldsymbol{\mu}_{PBO1}$) be the difference in the mean change from Baseline to Week 12 in the RSBQ total score and Δ_2 ($\boldsymbol{\mu}_{TRO2} - \boldsymbol{\mu}_{PBO2}$) be the difference in the mean CGI-I score at Week 12 between the trofinetide ($\boldsymbol{\mu}_{TRO}$) and placebo ($\boldsymbol{\mu}_{PBO}$) groups.

The null hypothesis is: $\Delta_i = 0; i = 1, 2.$

The alternative hypothesis is: $\Delta_i \neq 0; i = 1, 2.$

Primary Estimator

The hypothesis testing will be performed for Full Analysis Set using the direct likelihood MMRM method assuming missing data are missing at random (MAR). The MMRM for the

RSBQ analysis will include effects for treatment group, age group (5-10 years old, 11-15 years old, and 16-20 years old), Baseline RSBQ severity (<35 total score and ≥35 total score), visit, treatment-by-visit interaction as fixed effects, Baseline RSBQ total score, and the Baseline RSBQ total score-by-visit interaction as covariates and subjects as random effect. The MMRM for the CGI-I analysis will include effects for treatment group, age group (5-10 years old, 11-15 years old, and 16-20 years old), Baseline RSBQ severity (<35 total score and ≥35 total score), visit, treatment-by-visit interaction as fixed effects, subjects as random effect, and Baseline CGI-S score, and the Baseline CGI-S score-by-visit interaction as covariates. An unstructured covariance matrix will be used and the Kenward-Roger approximation will be used to adjust the denominator degrees of freedom.

The following is sample SAS code for the MMRM:

For RSBQ:

```
proc mixed data=xxx;
   class trt subjid visit age-group RSBQ-severity;
   model cfb-RSBQ-total-score = trt|visit age-group RSBQ-severity base-RSBQ
   base-RSBQ*visit/ ddfm=kr2;
   repeated visit / subject = subjid type=UN;
   LSmeans trt*visit / pdiff cl OM;
run;
```

For CGI-I:

```
proc mixed data=xxx;
   class trt subjid visit age-group RSBQ-severity;
   model CGI-I = trt|visit age-group RSBQ-severity base-CGI-S
   base-CGI-S*visit/ ddfm=kr2;
   repeated visit / subject = subjid type=UN;
   LSmeans trt*visit / pdiff cl OM;
run;
```

In the event that the model fails to converge using the unstructured covariance matrix, the following covariance structures will be modeled in the order given (i.e., from least parsimonious to most parsimonious): heterogeneous Toeplitz, heterogeneous compound symmetry, heterogeneous autoregressive(1), Toeplitz, compound symmetry, autoregressive(1), variance components. The first covariance structure that allows for convergence will be selected for the final model.

Summary statistics for the RSBQ total score (observed and change from Baseline) and the CGI-I score (observed), LS means, the between-group difference in LS mean with the

corresponding 95% confidence interval, p-value, and the effect size (Cohen's d) will be presented at each post-Baseline visit for Full Analysis Set.

The treatment effect size (Cohen's d) is calculated using the following formula:

Effect Size = LS mean difference / SD

SD is the model-based estimate, i.e., the estimated standard deviation from the modeled covariance matrix. The sign (+ or -) of the effect size will be chosen so that a positive value favors trofinetide.

LS mean ± SE from the MMRM models over time for the change from Baseline values by treatment group will be displayed in line plots for Full Analysis set.

The analyses for co-primary endpoints will also be performed for the subgroups of age group 1 (5-10 years old, 11-15 years old, and 16-20 years old), age group 2 (5-12 years old, 13-17 years old, and ≥18 years old), and Baseline RSBQ severity (<35 total score and ≥35 total score). In addition, subgroup analysis by *MECP2* mutation groupings will also be performed.

13.1.2 Sensitivity Analyses

The following sensitivity analyses of the co-primary efficacy endpoints are planned to account for intercurrent events.

13.1.2.1 **Pattern-Mixture Models Assuming Missing Not At Random (PMM-MNAR)**

The sensitivity analysis is implemented for Full Analysis Set using multiple imputations that are based on the distribution of placebo group responses over time to account for the intercurrent event of treatment discontinuations and missing assessments. The underlying assumption is that subjects with missing data due to early withdrawal evolve in the same way as placebo subjects that remain in the study.

The following steps are involved and the imputed values will be constrained to be within the limits of 0 - 90 for RSBQ total score and 1 - 7 for CGI-I score:

- Non-monotone (intermediate) missing data at Baseline (not applicable for CGI-I score), Week 2, Week 6, and Week 12 will be multiply imputed using the Markov chain Monte Carlo (MCMC) method to create 50 monotone datasets. The imputation models for post baseline RSBQ total score and CGI-I score will include effects as follows:

  Post baseline RSBQ total score: age group (5-10 years old, 11-15 years old, and 16-20 years old), Baseline RSBQ severity (<35 total score and ≥35 total score), Baseline RSBQ total score, and treatment group

CGI-I score: age group (5-10 years old, 11-15 years old, and 16-20 years old), Baseline RSBQ severity (<35 total score and ≥35 total score), Baseline CGI-S score, and treatment group.

- The monotone missing data will be imputed using a parametric, sequential linear regression method in which the missing data are imputed only on data from the placebo arm. A single imputation will be performed sequentially at each visit for each of the 50 imputed datasets. The predictors and their order in the PROC MI VAR statement for each visit are summarized in Table 5. The following SAS codes are to impute missing observations using the control-based pattern imputation method.

For RSBQ:

```
proc mi data=xxxx seed=2566003 nimpute=1 out=outname;
  by _Imputation_;
  class Trt Baseline_RSBQ_severity Age_group;
  monotone reg (/details);
  mnar model( W2_RSBQ W6_RSBQ W12_RSBQ / modelobs= (Trt='Placebo'));
  var Age_group Baseline_RSBQ_severity Baseline_RSBQ W2_RSBQ W6_RSBQ
W12_RSBQ;
run;
```

For CGI-I:

```
  by _Imputation_;
  proc mi data=xxxx seed=2566003 nimpute=1 out=outname;
  class Trt Baseline_RSBQ_severity Age_group;
  monotone reg (/details);
  mnar model( W2_CGI-I W6_CGI-I W12_CGI-I / modelobs= (Trt='Placebo'));
  var Age_group Baseline_RSBQ_severity Baseline_CGI-S W2_CGI-I W6_CGI-I
W12_CGI-I;
run;
```

Table 5 Imputation Predictors

Visit	RSBQ Total Score - Predictors	CGI-I Score - Predictors
Week 2	Age group, Baseline RSBQ severity, Baseline RSBQ total score	Age group, Baseline RSBQ severity, Baseline CGI-S score
Week 6	Age group, Baseline RSBQ severity, Baseline RSBQ total score, Week 2 RSBQ total score	Age group, Baseline RSBQ severity, Baseline CGI-S score, Week 2 CGI-I score
Week 12	Age group, Baseline RSBQ severity, Baseline RSBQ total score, Week 2 RSBQ total score, Week 6 RSBQ total score	Age group, Baseline RSBQ severity, Baseline CGI-S score, Week 2 CGI-I score, Week 6 CGI-I score

Note: Age group (5-10 years old, 11-15 years old, and 16-20 years old); Baseline RSBQ severity (<35 total score and ≥35 total score).

- The change from Baseline to each post baseline visit values will then be calculated and analyzed for each of the 50 fully imputed datasets using the analysis of co-variance (ANCOVA) model. The ANCOVA model for RSBQ total score will have treatment group, Baseline RSBQ severity, and age group as fixed factors and Baseline RSBQ total score as a covariate; the ANCOVA model for CGI-I score will have treatment group, age group, and Baseline RSBQ severity as fixed factors and Baseline CGI-S score as a covariate.
- The results will be summarized by post baseline visit using the SAS MIANALYZE procedure to yield a combined estimate for treatment effect with its associated 95% confidence interval (CI) and p-value.

13.1.2.2 Sensitivity Analysis for Missing Data Due to COVID-19 PHE (PMM-COVID-19-PHE-MAR Variant)

The sensitivity analysis differs from Section 13.1.2.1 by using a different assumption for missing data due to COVID-19 PHE related withdrawal (after intermediate data has been imputed). This variant will operate under the assumption that missing data after withdrawal due to COVID-19 PHE related withdrawal is MAR, while missing data after withdrawal not due to COVID-19 PHE is MNAR and assumed to evolve in the same way as placebo subjects that remain in the study.

This sensitivity analysis involves extra steps from the previously described sensitivity analysis. After the monotone missing data sets are imputed the data sets will be duplicated and made into two partitions 1) the original 50 monotone missing data sets 2) the 50 monotone data sets consisting of only subjects that did not have COVID-19 PHE related withdrawal.

Using the original 50 monotone missing data sets (partition 1); the monotone missing data due to COVID-19 PHE withdrawal will be imputed using a parametric, sequential linear regression method based on data from the respective treatment arm. Then the subjects who had COVID-19 PHE related withdrawal will be merged into the partition of the 50 monotone data sets consisting of only subjects that did not have COVID-19 PHE related withdrawal (partition 2). The monotone missing data in this combined partition will be imputed using a parametric, sequential linear regression method based on data from the placebo arm.

Then analysis will be performed as previously specified in Section 13.1.2.1.

13.1.2.3 Sensitivity Analysis Accounting for Impact of Remote Assessments on the Co-Primary Endpoints

To assess the impact of the intercurrent events of remote assessments on the primary analysis, an indicator variable will be assigned to flag subjects that had assessments done remotely for the co-primary efficacy endpoints at any visit including baseline (Yes/No); the flag will be defined for each of the co-primary efficacy variables, separately. The remote visit flag indicator variable and the interaction between treatment group and remote visit flag indicator variable will be included as factors in the MMRM model.

13.1.2.4 Additional Summaries Evaluating the Impact of COVID-19 on the Primary Endpoint

To assess the impact of COVID-19 PHE on data collection, the following will be summarized:

- The number and percentage of subjects impacted by at least one of the following COVID-19 PHE-related ICEs; and the number and percentage of subjects impacted by each of the following COVID-19 PHE-related ICEs:
    - Treatment Discontinuation due to COVID-19 PHE
    - Intermediate missing data due to COVID-19 PHE
    - Remote assessments due to COVID-19 PHE)
- The number and percentage of subjects with remote assessments not due to COVID-19 PHE
- The number and percentage of subjects with missed assessments for the co-primary endpoints by visit
    - Due/not due to COVID-19 PHE
- The number and percentage of subjects with remote assessments for the co-primary endpoints by visit
    - Due/not due to COVID-19 PHE

13.1.2.5 Derived Baseline RSBQ Randomization Strata

Baseline RSBQ severity score (<35 total score and ≥35 total score) was used as randomization strata without reversing the score for item 31 "Uses eye gaze to convey feelings, needs and wishes.". A sensitivity analysis will be performed using the same model described for the co primary endpoints and the key secondary endpoint, except the model will replace the Baseline RSBQ severity strata from IRT with a derived Baseline RSBQ severity strata score where item 31 is reversed.

13.1.3 Supportive Analysis

Supportive analysis of the co-primary endpoints will be analyzed, similarly to the primary analysis, using the Per-Protocol set.

13.2 Key Secondary Efficacy Endpoints

The key secondary endpoint, change from baseline to Week 12 in the CSBS-DP-IT Social Composite Score for the Full Analysis Set, will be analyzed using the direct likelihood MMRM method assuming missing data are MAR. The MMRM model will include effects for treatment group, age group (5-10 years old, 11-15 years old, and 16-20 years old), Baseline RSBQ severity (<35 total score and ≥35 total score), visit, treatment-by-visit interaction as fixed effects, Baseline CSBS-DP-IT Social Composite Score, and the Baseline CSBS-DP-IT Social Composite Score -by-visit interaction as covariates and subjects as random effect. Supportive analyses of the key secondary endpoint will use the Per-Protocol set.

13.3 Other Secondary Efficacy Endpoints

Analyses for the other secondary efficacy endpoints in this section will be performed for Full Analysis Set using the observed values.

RTT-HF, RTT-AMB, RTT-COMC, RTT-VCOM, and CGI-S

The change from Baseline will be analyzed using the MMRM method similar to the primary analysis for the co-primary endpoints. The MMRM model will include effects for treatment group, age group (5-10 years old, 11-15 years old, and 16-20 years old), Baseline RSBQ severity (<35 total score and ≥ 35 total score), visit, the treatment-by-visit interaction, Baseline score, and the Baseline score-by-visit interaction.

Summary statistics (observed and change from Baseline), LS means, the between-treatment difference in LS mean with the corresponding 95% confidence interval, p-value, and the effect size (Cohen's d) will be presented at each post-Baseline visit.

Quality of Life Rating of the ICND, ICND Total Score, and RTT-CBI

The change from Baseline to Week 12 will be summarized by treatment group and analyzed using the ANCOVA model with treatment group, age group (5-10 years old, 11-15 years old, and 16-20 years old), and Baseline RSBQ severity (<35 total score and ≥ 35 total score) as fixed factors and the corresponding Baseline value as a covariate.

13.4 Exploratory Efficacy Analyses

13.4.1 CGI-I Responder Analysis

Responder analysis based on CGI-I will be performed as exploratory analysis. Responder is defined as CGI-I scores of 1 or 2 (much or very much improved). Responder analysis will be performed in two ways:1) Missing CGI-I will be imputed as non-responder and 2) using observed cases only (subjects with missing CGI-I will be excluded). The proportion of responders will be presented at each visit. Treatment comparison will be performed using logistic regression adjusted for baseline CGI-S, age group, and baseline RSBQ severity. Analysis for RSBQ subscales will also be performed similarly as that for the change from baseline in RSBQ total score.

Histograms of CGI-I by visit will be presented.

13.4.2 Hypothetical Strategy Estimand

An exploratory analysis will be performed for the co-primary endpoints that will differ from the primary analysis which uses the treatment policy estimand. For this exploratory analysis, the intercurrent event is permanently discontinuing the study treatment more than 2 and half days before final RSBQ/CGI-I assessment. The hypothetical strategy will be used to estimate the hypothetical effect if all subjects in the full analysis set adhered to the prescribed treatment until their final co-primary efficacy assessments. In this analysis, the final RSBQ/CGI-I assessment data observed after more than 5 doses from the last dose (i.e., missed the last 5 scheduled doses which is defined as the morning and evening doses of the prior two days and the morning dose of the visit of the efficacy assessment) will be considered missing and excluded from the analysis. The same MMRM model as the primary analysis will be used after excluding the data observed beyond 5 doses from last dose.

14. SAFETY ANALYSES

All safety analyses will be performed using the actual treatment for the Safety Analysis Set.

14.1 Adverse Events

Adverse events will be coded using MedDRA dictionary, Version 23.0 or newer.

An AE (classified by preferred term) will be considered a treatment-emergent AE (TEAE) if started after first dose administration and no later than last dose date + 30 days. AEs reported on Day 1 based on Baseline (pre-dose) findings (e.g., clinically significantly abnormal vital signs, laboratory test results, or electrocardiogram parameters) will not be considered as TEAEs.

The event counts, the number, and percentage of subjects reporting TEAEs in each treatment group will be tabulated by system organ class (SOC) and preferred term; by SOC, preferred term, and maximum severity; and by SOC, preferred term, and relationship to study drug. If more than one TEAE occurs with the same preferred term for the same subject, then the subject will be counted only once for that preferred term using the most severe and most related occurrence for the summarization by severity and by relationship to study drug. The display in these tables will be sorted alphabetically by SOC and then by descending subject frequency for the preferred terms (counts from trofinetide group) within each SOC.

The event counts, the number and percentage of subjects with any TEAEs will also be tabulated by preferred term without SOCs. This table will be sorted by descending subject frequency within trofinetide group.

The incidence of most frequently reported (preferred terms reported by ≥ 5% of subjects in any treatment group) TEAEs, SAEs reported after treatment start, TEAEs leading to drug withdrawn, and TEAEs related to study drug will be summarized by SOC, preferred term, and treatment group. The tables will be sorted alphabetically by SOC and then by descending frequency within each SOC in trofinetide group. In addition, the incidence of fatal treatment-emergent AEs (i.e., events that cause death) will be summarized separately by SOC and preferred term.

These summary tables except for the most frequently reported TEAEs tables will also be presented by relationship to COVID-19 Infection (Not related to COVID-19 Infection vs. Related to COVID-19 Infection).

An AE listing by subject will display all events, including those which occur during screening, and will include the verbatim term in addition to the MedDRA SOC and preferred term. This listing will also include all relevant eCRF data associated with the event: date of onset, date resolved, date of last dose, severity, frequency, outcome, relationship to study

drug, and action taken with study drug. Separate listings will be presented for subjects with treatment-emergent SAEs, related TEAEs, TEAEs leading to drug withdrawn, fatal TEAEs (if any), and TEAEs Related to COVID-19 Infection.

14.2 Clinical Laboratory Variables

Due to COVID-19 PHE related disruptions, it is possible that some test results may be collected from a local laboratory. Local laboratory results and the associated normal ranges will be converted to SI units; local laboratory results, in SI units, will then be normalized to central lab ranges to be included in summary data analysis together with the central laboratory results. The normalization will be performed using the following scale transformation equation:

$$s = L_s + (x - L_x)\frac{U_s - L_s}{U_x - L_x}$$

where s is the normalized individual laboratory value to be used for summary; x is the original value from the local lab; L_x and U_x are the lower and upper limits from the local lab; L_s and U_s are the lower and upper limits from the central lab.
For labs with only a single upper (or lower) limit, the following scale transformation equation will be used:

$$s = x\frac{U_s}{U_x}$$

where s is the normalized individual laboratory value to be used for summary; x is the original value from the local lab; U_s is the upper (or lower) limit from the central lab; U_x is the upper (or lower) limit from the local laboratory. Local laboratory results and normalized results will be included in data listings. Only central lab and normalized local lab results will be used for summary of change from baseline, shift, and potentially clinically important (PCI) analyses.
Clinical laboratory assessments are performed at Screening Visit 1, Baseline (Week -3 to 0), Week 2, Week 6 (no urinalysis), and Week 12/EOT.

- Clinical chemistry serum tests include the following:
  - Sodium (Na), potassium (K), chloride (Cl), phosphorus (P), calcium (Ca), magnesium (Mg), carbon dioxide (CO_2), blood urea nitrogen (BUN), creatinine (CR), uric acid
    - Mg will only be performed at Visit 1 (Screening)
  - Alanine aminotransferase (ALT), aspartate aminotransferase (AST), gamma-glutamyl transpeptidase (GGT), alkaline phosphatase (ALP), total bilirubin (TBIL), lactate dehydrogenase (LDH)

    - HbA1c
        - HbA1c will only be performed at Visit 1 (Screening)
    - Glucose
    - Albumin (ALB), total protein
    - Thyroid stimulating hormone (TSH), free T3, and free T4
        - Thyroid function tests will be performed at Visit 1 (Screening), Visit 2 (Baseline), and Visit 5
- Pregnancy test
    - A serum pregnancy test will be performed at all designated visits for subjects of childbearing potential
- Hematology tests include the following:
    - Complete blood count (CBC) including:
        - White blood cell (WBC) count
        - Complete differential (relative and absolute)
        - Hematocrit (Hct), hemoglobin, red blood cells (RBC), platelets
        - Reticulocyte count
- Urinalysis tests include the following:
    - Blood, RBCs, WBCs, protein, glucose, ketones, specific gravity, pH

Clinical laboratory values (in Système International [SI] units) and the change from Baseline values will be summarized by treatment group at each post-Baseline visit using descriptive statistics. The overall minimum, maximum as well as the last post-Baseline observed and change from Baseline values will also be summarized. For urinalysis with categorical results, the number and percentage of subjects will be tabulated by category at Baseline and each post-Baseline visit, and the denominator is the number of subjects with non-missing values for the given parameter, visit and treatment group.

Laboratory values will also be summarized in shift tables by treatment group, to determine the number and percentage of subjects with values classified as below, within, and above normal ranges at each post-Baseline visit relative to the same classification at the Baseline visit. For the by-visit shift summary, the denominator is the number of subjects with non-missing values at Baseline and the given visit for the given parameter and treatment group. For the shift to the overall post-Baseline minimum or maximum, all post-Baseline values will

be considered, including unscheduled and out of window values and the denominator is the number of subjects with non-missing Baseline value and at least 1 post-Baseline value for the given parameter and treatment group.

Clinical laboratory values are potentially clinically important (PCI) if they meet either the low or high PCI criteria listed in Tables 6 and 7. The number and percentage of subjects with post-Baseline PCI values for each of the categories in Tables 6 and 7 will be summarized by treatment group for selected parameters. For the overall post-Baseline summaries of PCI values, all post-Baseline values will be considered, including unscheduled and out of window values. Subjects with multiple PCI values for a given parameter will be counted only once for that parameter. For the overall post-Baseline summary, the numerator of the percentage is the number of subjects with at least 1 post-Baseline PCI laboratory value for the given parameter and treatment group, and the denominator is the number of subjects with at least 1 post-Baseline laboratory value for the given parameter and treatment group.

Table 6 Criteria for Potentially Clinically Important Laboratory Values – Hematology and Chemistry

Analyte	Conventional Unit	Low PCI Criteria	High PCI Criteria	SI Unit	Low PCI Criteria	High PCI Criteria
**Hematology (whole blood)**						
Hemoglobin (female)	g/dL	<10	>17	g/L	<100	>170
Hematocrit (female)	%	<30	>50	L/L	<0.3	>0.5
Leukocyte (White Blood Cell Count)	x 10^3/uL	≤2.8	≥15	x 10^9/L	≤2.8	≥15
Neutrophils	x 10^3/uL	≤1.5	No upper limit	x 10^9/L	≤1.5	No upper limit
Platelet Count	x 10^3/uL	≤75	≥700	x 10^9/L	≤75	≥700
**Chemistry (serum or plasma)**						
ALT	U/L	No lower limit	≥3 X ULN	U/L	No lower limit	≥3 X ULN
AST	U/L	No lower limit	≥3 X ULN	U/L	No lower limit	≥3 X ULN
Total Bilirubin	mg/dL	No lower limit	≥1.5 ULN	umol/L	No lower limit	≥1.5 ULN
BUN	mg/dL	No lower limit	≥30.0	mmol/L	No lower limit	≥10.71

Table 6 Criteria for Potentially Clinically Important Laboratory Values – Hematology and Chemistry (Continued)

Analyte	Conventional Unit	Low PCI Criteria	High PCI Criteria	SI Unit	Low PCI Criteria	High PCI Criteria
Sodium	mEq/L	≤125	≥155	mmol/L	≤125	≥155
Potassium	mEq/L	≤3.0	≥5.5	mmol/L	≤3.0	≥5.5
Calcium, total	mg/dL	<8.0	>11.0	mmol/L	<2.0	>2.75
Lactate Dehydrogenase (LDH)	U/L	No lower limit	≥3 X ULN	U/L	No lower limit	≥3 X ULN
Alkaline Phosphatase	U/L	No lower limit	≥3 X ULN	U/L	No lower limit	≥3 X ULN
Uric acid (female)	mg/dL	No lower limit	≥8.5	umol/L	No lower limit	≥505.75
Albumin	g/dL	≤2.6	≥6.0	g/L	≤26	≥60
Total Protein	g/dL	≤5.0	≥10.0	g/L	≤50	≥100
Chloride	mEq/L	≤85	≥120	mmol/L	≤85	≥120
Glucose (random)	mg/dL	≤45.1	≥200.0	mmol/L	≤2.48	≥11
Serum Creatinine	mg/dL	Not Applicable	>1.5 ULN	umol/L	Not Applicable	>1.5 ULN
Gamma-Glutamyl Transferase (GGT)	U/L	Not Applicable	≥3 ULN	U/L	Not Applicable	≥3 ULN

Table 7 Criteria for Potentially Clinically Important Laboratory Values - Urinalysis

Urinalysis (qualitative dipstick)	Low PCI Criteria	High PCI Criteria
Blood (occult blood)	Not Applicable	≥ Moderate
Protein	Not Applicable	≥ 100 mg/dL
Glucose	Not Applicable	≥ 500 mg/dL

Clinical laboratory data will be displayed in data listings with date and study day of collection. All units will be displayed according to SI conventions for units. Out of range values will be flagged in the data listings (i.e., 'L' or 'H'). A separate listing will be provided for a subset of the chemistry, hematology, and urinalysis analytes with values classified as PCI.

At the initiation of the study, the main reference ranges (the reference ranges provided by the central laboratory validated for use for the general population) were used for the purpose of flagging abnormal laboratory values for the investigator to assess clinical significance. During the study, at the request of a leading investigator an alternative set of reference ranges, more specific to patients with Rett syndrome rather than to the general population,

was used for the purpose of flagging abnormal lab values for the investigator to assess clinical significance.

All analysis will be done using the main reference ranges, not the alternative ranges. During the course of the study the main reference ranges were updated by the central laboratory. The latest version of the reference ranges provided by the central laboratory will be used for all analysis; PCI status and categorical labs (Low, Normal, High) and shifts will be derived from the latest version of the main reference range. Both of the reference ranges (main and alternative) and the corresponding categorical assessment will be listed.

The pregnancy results (positive or negative) will be presented in a listing.

14.3 Vital Signs

Vital signs will be collected throughout the study; height will be measured at Screening and Week 12/EOT; weight will be measured at Screening, Baseline, and Week 12/EOT. Observed vital signs including weight and BMI and the changes from Baseline at each post-Baseline visit will be summarized by treatment group using descriptive statistics.

Vital sign values will be considered PCI if they meet the criteria listed in Table 8. The number and percentage of subjects with post-Baseline vital signs that are PCI will be summarized by treatment group at each post-Baseline visit and for overall post-Baseline. For the overall post-Baseline summaries, all post-Baseline values will be considered, including unscheduled and out of window values. Subjects with multiple PCI values for a given parameter will be counted only once for that parameter. For the by-visit summary, the numerator for the percentage is the number of subjects with a post-Baseline PCI vital sign for the given parameter, visit and treatment group, and the denominator is the number of subjects with non-missing values for the given parameter, visit and treatment group. For the overall post-Baseline summary, the numerator for the percentage is the number of subjects with at least 1 post-Baseline PCI vital sign for the given parameter and treatment group, and the denominator is the number of subjects with at least 1 post-Baseline vital sign for the given parameter and treatment group. A listing of overall and of subjects with any PCI vital sign values will be provided.

Table 8 Criteria for Potentially Clinically Important Vital Signs

Vital Sign Parameter	Unit	Criteria			
		Observed Value	And/Or	Change Relative to Baseline	Change from Supine to Standing
Systolic blood pressure (supine or sitting)	mmHg	≥180	And	Increase of ≥20	-
		≤90	And	Decrease of ≥20	-
Diastolic blood pressure (supine or sitting)	mmHg	≥105	And	Increase of ≥15	-
		≤50	And	Decrease of ≥15	-
Pulse (supine or sitting)	bpm	≥120	And	Increase of ≥15	-
		≤50	And	Decrease of ≥15	-
Weight	kg	Not Applicable		Increase of ≥7%	-
				Decrease of ≥7%	-

14.4 Electrocardiogram (ECG)

12-lead ECGs are collected throughout the study at every visit.

When ECG is collected multiple times (e.g. at Screening visit, Baseline visit both before dosing and 2-3 hours after dosing, and Week 12/EOT/ET visit), the average of all available values on the same day will be considered as one assessment for the summaries, except for Day 1 where averages will be by pre-dose and post-dose averages. The averages will be rounded to integer values.

Observed (average) of ECG variables (e.g., heart rate, PR interval, QRS interval, QT interval, QTcB and QTcF interval) and the changes from Baseline at each assessment time point will be summarized by treatment group.

Observed (average) of QTcB and QTcF will also be categorized into the following categories (msec) and the number and percentage of subjects in each category will be summarized by treatment group at each visit and for the overall post-Baseline maximum:

- Observed: ≤450, 451 - ≤480, 481 - ≤500, and >500; >450; >480.
- Change from Baseline: ≤10, 11 – 30, 31 – 60, and >60; >30.

For cardiologist's interpretations, the number and percentage of subjects with ECG results that are determined as normal or abnormal will be summarized at scheduled visits. The overall post-baseline worst interpretation will also be summarized (i.e. if a subject has one or more post-baseline ECG results that is/are considered as abnormal, this subject will be counted in the abnormal category). Cardiologist's interpretations will also be summarized in a shift table. The shifts from Baseline to overall post-Baseline worst interpretation will also be presented. For the by-visit shift summary, the denominator is the number of subjects with

non-missing cardiologist's interpretation at Baseline and the given visit for the given treatment group. For the summaries of shift from Baseline to the overall post-Baseline worst interpretation, the denominator is the number of subjects with non-missing Baseline and at least 1 post-Baseline cardiologist's interpretation for the given treatment group.

Electrocardiogram variable average values will be considered PCI if they meet the criteria listed in Table 9. The number and percentage of subjects with post-baseline PCI values will be summarized by treatment group at each post-Baseline visit and for overall post-Baseline. For the by-visit summary, the numerator for the percentage is the number of subjects with a post-Baseline PCI ECG for the given parameter, visit and treatment group, and the denominator is the number of subjects with non-missing values for the given parameter, visit and treatment group. For the overall post-Baseline summary, the numerator for the percentage is the number of subjects with at least 1 post-Baseline PCI ECG for the given parameter and treatment group, and the denominator is the number of subjects with at least 1 post-Baseline ECG value for the given parameter and treatment group. A listing of overall and of all subjects with any PCI ECG values will be provided.

Table 9 Criteria for Potentially Clinically Important ECG Values

ECG Parameter	Unit	High PCI Criteria
QRS Interval	Msec	≥120
PR Interval	Msec	≥220
QTcB	Msec	>500
QTcF	Msec	>500
QTcB: change from baseline	>60 msec	
QTcF: change from baseline	>60 msec	

14.5 Physical Examination

Physical examinations are performed throughout the study at every visit in the clinic. Physical examination results (normal, abnormal, and not done) will be summarized in a frequency table by treatment group, body system and visit. A listing of overall physical examination data will be listed.

14.6 Other Safety Endpoints

There are no other safety endpoints in this study.

15. CLINICAL PHARMACOKINETIC AND PHARMACODYNAMIC ANALYSES

For trofinetide-treated subjects, whole blood concentration for trofinetide will be listed. Whole blood concentration data for trofinetide will be summarized for the PK Analysis Set at each visit using descriptive statistics. Concentrations that are below the limit of quantification (BLQ) will be displayed as “BLQ” in the data listings and imputed as 0 for computing summary statistics.

If data allow, population PK and PK/PD analyses will be performed to further characterize the PK profile and exposure response relationship of trofinetide using measures of safety and efficacy parameters. The results of population PK and PK/PD modeling will be presented in a separate report. Trofinetide whole blood concentration data will remain blinded until the unblinding of the clinical database at the end of the study.

16. INTERIM ANALYSIS

No interim analysis is planned in this study.

17. DATA MONITORING/REVIEW COMMITTEE

An independent Data and Safety Monitoring Board (DSMB) will review safety information on a regular basis throughout the study. The DSMB will be independent of the Sponsor and will be empowered to recommend stopping the study due to safety concerns. The DSMB may review blinded, unblinded, or partially unblinded data, but the Sponsor and the Investigators will remain blinded to the data provided to the DSMB until the official unblinding of the database at the completion of the study. The membership, activities, responsibilities, and frequency of meetings will be described separately in the DSMB charter.

18. COMPUTER METHODS

Statistical analyses will be performed using Version 9.4 (or newer) of SAS® on a qualified and validated environment.

Validation and quality control of the tables, listings and figures containing the results of the statistical analyses will follow appropriate standard operating procedures.

19. CHANGES TO ANALYSES SPECIFIED IN PROTOCOL

No changes are made to the analyses specified in the protocol.

20. REFERENCES

EMA (2009). Guideline on Missing Data in Confirmatory Clinical Trials, European Medicines Agency, London, UK.

ICH E9(R1): Addendum on Estimands and Sensitivity Analysis in Clinical Trials to the Guideline on Statistical Principles for Clinical Trials, Adopted on November 2019

FDA Guidance on Conduct of Clinical Trials of Medical Products during COVID-19 Pandemic Guidance for Industry, Investigators, and Institutional Review Boards (March 2020)

Points to consider on implications of Coronavirus disease 4 (COVID-19) on methodological aspects of ongoing clinical 5 trials, March 25 2020.

21. APPENDICES

21.1 Schedule of Assessments

Period	Screening	Baseline	Double-blind Treatment Period			Safety Follow-up[b]
Visit Week		0	2[a]	6	12/EOT/ET	EOT/ET+ 30 days
Visit Number	1	2	3	4	5	
Visit window (days)	N/A	N/A	±3	±4	+3	+4
Type of Visit[k]	Clinic or Off-site					Telephone or Telemedicine
Informed consent	X				X[c]	
Inclusion/exclusion criteria	X	X				
Medical history and demographics	X					
Confirm documented Rett diagnosis and *MECP2* mutation	X					
Rett syndrome history	X					
Rett Syndrome Clinical Severity Scale	X					
Physical examination[k]	X	X	X	X	X	
Vital signs[d]	X	X	X	X	X	
Height	X				X	
Weight	X	X	X[k]	X[k]	X[k]	
12-lead electrocardiogram (ECG)[e]	X	X[e]	X	X	X	
Clinical laboratory tests (hematology, chemistry)	X	X	X	X	X	
Urinalysis	X	X	X		X	
TSH, Free T3, Free T4	X	X			X	
HbA1c	X					
Serum pregnancy test[f]	X		X	X	X	
Blood samples for pharmacokinetics		X[g]	X[h]	X[h]	X[h]	
Blood sample for optional analysis for biomarkers[i]		X			X	
Rett Syndrome Behaviour Questionnaire (RSBQ)	X	X	X	X	X	
Clinical Global Impression–Improvement (CGI-I)			X	X	X	

21.1 Schedule of Assessments (Continued)

Period	Screening	Baseline	Double-blind Treatment Period			Safety Follow-up[b]
Visit Week		0	2[a]	6	12/EOT/ET	EOT/ET+ 30 days
Visit Number	1	2	3	4	5	
Visit window (days)	N/A	N/A	±3	±4	+3	+4
Type of Visit[k]	Clinic or Off-site					Telephone or Telemedicine
Clinical Global Impression–Severity (CGI-S)	X	X	X	X	X	
Communication and Symbolic Behavior Scales-Developmental Profile™ Infant-Toddler (CSBS-DP-IT) Checklist		X	X	X	X	
Rett Syndrome Clinician Rating of Hand Function (RTT-HF)		X	X	X	X	
Rett Syndrome Clinician Rating of Ability to Communicate Choices (RTT-COMC)[k]		X	X	X	X	
Rett Syndrome Clinician Rating of Ambulation and Gross Motor Skills (RTT-AMB)		X	X	X	X	
Rett Syndrome Clinician Rating of Verbal Communication (RTT-VCOM)		X	X	X	X	
Rett Syndrome Caregiver Burden Inventory (RTT-CBI)		X			X	
Impact of Childhood Neurologic Disability Scale (ICND)		X			X	
Dispensing and review of semi-structured caregiver diary	X	X	X	X	X	
Concomitant medications	X	X	X	X	X	X
Assessment of adverse events	X	X	X	X	X	X
Randomization		X				
Study drug dispensed[j]		X				
Authorization of study drug dispensation[j]		X-----------------------X				

21.1 Schedule of Assessments (Continued)

Period	Screening	Baseline	Double-blind Treatment Period			Safety Follow-up[b]
Visit Week		0	2[a]	6	12/EOT/ET	EOT/ET+ 30 days
Visit Number	1	2	3	4	5	
Visit window (days)	N/A	N/A	±3	±4	+3	+4
Type of Visit[k]	Clinic or Off-site					Telephone or Telemedicine
Study drug return[j]			X------------------------------X			
Study drug accountability[j]			X	X	X	

Abbreviations: EOT=end of treatment; ET=early termination; HbA1c=glycosylated hemoglobin; *MECP2*=methyl-CpG-binding protein 2 gene; TSH=thyroid stimulating hormone

a Timing of post-Baseline visits will be calculated from the first day of dosing (Day 1) (i.e. the Week 2 visit will occur 2 weeks [±3 days] after the first day of dosing).

b Subjects who roll over into the OLE study will not have the safety follow-up telephone call.

c For subjects who decide to continue into the open-label extension (OLE) study, informed consent for the OLE **must be** obtained prior to performing the Week 12/EOT procedures.

d Vital signs will include body temperature, resting respiration rate, sitting systolic and diastolic blood pressure, and pulse rate. The sitting blood pressure will be measured after the subject has been sitting for ≥3 minutes.

e ECGs will be completed in triplicate at Visit 1 (Screening), at Visit 2 (Baseline) both before dosing and 2-3 hours after dosing, and at Visit 5 (Week 12/EOT/ET). A single ECG will be completed at Visit 3 (Week 2) and Visit 4 (Week 6).

f For subjects who have reached menarche and have not had surgical sterilization.

g A predose PK blood sample must be collected before administration of study drug. A postdose PK blood sample will be collected at the end of ECG assessment 2-3 hours after study drug administration.

h PK samples at Visits 3, 4, and 5 will be collected at one of the following time intervals: 1) 2-3 hours after dosing OR 2) 4-6 hours after dosing OR 3) 7-11 hours after dosing. Every effort should be made to collect the PK samples at discrete time intervals during Visits 3, 4, and 5. However, if the interval is the same across these visits, then the collection time should vary within that interval.

i Participation in the effort to identify biomarkers is an optional component of the study requiring a separate informed consent, which may be obtained at any time during the study. If consent is obtained after Baseline, only the sample at Visit 5 (or upon early termination) will be taken.

j Investigational product will be shipped directly to the subject. Confirmation of any delivery to the subject will be made by a visiting nurse. Study drug shipment, return, and accountability will be performed in accordance with the drug distribution plan. In addition, study drug will be dispensed at the site during the Baseline visit when the visit is conducted in the clinic.

k Study visits may be done off-site rather than in the clinic with the prior approval of the Sponsor or Medical Monitor. Screening, Baseline, and EOT visits should be done in the clinic whenever possible. When a study visit takes place off-site, the physical examination will not be required. Weight should be measured whenever possible at off-site visits. The RTT-COMC should be completed if possible, but it is not required.

21.2 Summary of Version Changes

Version No:	Document History<br>Description of Update	Author(s)	Version Date
1.0	Original version	[redacted]	04 January 2021
2.0	Added *MECP2* Groupings; added definitions of RSBQ subscores; clarified analysis visit windowing; added additional sensitivity analyses; added additional descriptive analysis to assess COVID-19 PHE impact to data collection	[redacted]	20 May 2021
3.0	Updated *MECP2* Groupings; clarified reference ranges used over the course of the study, clarified text throughout.	[redacted]	20 October 2021
