# Supplementary material for: Trofinetide for the treatment of Rett syndrome: a randomized phase 3 study
Source: Nat Med. 2023 Jun 8;29(6):1468–75. doi: 10.1038/s41591-023-02398-1 (PMC10287558; doi:10.1038/s41591-023-02398-1)
Supplement: Supplementary file 4 — Final version of the study protocol. [file 41591_2023_2398_MOESM4_ESM.pdf]

ACADIA®
Pharmaceuticals

CLINICAL STUDY PROTOCOL

A Randomized, Double-Blind, Placebo-Controlled, Parallel-Group Study of Trofinetide for the Treatment of Girls and Women with Rett Syndrome

Protocol Number: ACP-2566-003

Amendment 2

Original Protocol Date: 02 April 2019

Protocol Amendment 1 Date: 27 April 2020

Protocol Amendment 2 Date: 07 August 2020

Protocol Template Version: 1.0

Confidentiality Statement

This protocol is the confidential information of ACADIA Pharmaceuticals Inc. and is intended solely for the guidance of the clinical investigation. This protocol may not be disclosed to parties not associated with the clinical investigation or used for any purpose without the prior written consent of ACADIA Pharmaceuticals Inc.

SPONSOR SIGNATURE PAGE

Title: A Randomized, Double-Blind, Placebo-Controlled, Parallel-Group Study of Trofinetide for the Treatment of Girls and Women with Rett Syndrome

ACADIA Head of Clinical Development:

[redacted]

[redacted]

Signature Date

ACADIA Project Lead:

[redacted]

[redacted]

Signature Date

PLACEHOLDER FOR E-SIGNATURE PAGE

DECLARATION OF INVESTIGATOR

I confirm that I have read the above protocol. I understand it, and I will work according to the moral, ethical, and scientific principles governing clinical research as set out in the principles of Good Clinical Practice, as required by International Council for Harmonisation of Technical Requirements for Pharmaceuticals for Human Use (ICH) Guideline E6 and as described in the United States (US) Code of Federal Regulations (CFR) 21 CFR parts 50, 54, 56, 312, and according to applicable local requirements.

Confidentiality Statement

The confidential information in this document is provided to you as a Principal Investigator or Consultant for review by you, your staff, and the applicable institutional review board/ethics committee. Your acceptance of this document constitutes agreement that you will not disclose the information contained herein to others without written authorization from the Sponsor.

Principal Investigator

[redacted]

PROTOCOL SYNOPSIS[1]

**Protocol Number**	ACP-2566-003
**EudraCT Number**	Not applicable
**Protocol Title**	A Randomized, Double-Blind, Placebo-Controlled, Parallel-Group Study of Trofinetide for the Treatment of Girls and Women with Rett Syndrome
**Name of Investigational Product**	Trofinetide oral solution
**Indication**	Rett syndrome
**Phase of Development**	3
**Sponsor**	ACADIA Pharmaceuticals Inc.<br>[redacted]

**Primary Objective**	**Co-Primary Endpoints**
• To investigate the efficacy of treatment with oral trofinetide versus placebo in girls and women with Rett syndrome	• Rett Syndrome Behaviour Questionnaire (RSBQ) total score – Change from Baseline to Week 12<br>• Clinical Global Impression–Improvement (CGI-I) Score at Week 12
**Key Secondary Objective**	**Key Secondary Endpoint**
• To investigate the efficacy of treatment with oral trofinetide versus placebo on ability to communicate in girls and women with Rett syndrome	Change from Baseline to Week 12 in:<br>• Communication and Symbolic Behavior Scales Developmental Profile™ Infant-Toddler Checklist – Social Composite Score (CSBS-DP-IT Social)

[1] NOTE: In this protocol, as in FDA guidance documents, the use of the word "should" means that something is suggested or recommended, but not required. If something "may be" done, the meaning is that something is allowed, but not required. The terms "must" and "will" mean that something is required.

Other Secondary Objectives	Other Secondary Endpoints
• To investigate the benefit of treatment with oral trofinetide versus placebo on overall quality of life for girls and women with Rett syndrome<br>• To investigate the efficacy of treatment with oral trofinetide versus placebo in girls and women with Rett syndrome on:<br>o Hand function<br>o Ambulation and other gross motor skills<br>o Ability to communicate choices and preferences<br>o Ability to communicate verbally<br>• To investigate the efficacy of treatment with oral trofinetide versus placebo on a global assessment of the severity of illness in girls and women with Rett syndrome<br>• To investigate the benefit of treatment with oral trofinetide versus placebo on the burden on caregivers of girls and women with Rett syndrome<br>• To investigate the benefit of treatment with oral trofinetide versus placebo on the impact of the disability on the child's and family's everyday life	Change from Baseline to Week 12 in:<br>• Overall Quality of Life Rating of the Impact of Childhood Neurologic Disability (ICND) Scale<br>• Rett Syndrome Clinician Rating of Hand Function (RTT-HF)<br>• Rett Syndrome Clinician Rating of Ambulation and Gross Motor Skills (RTT-AMB)<br>• Rett Syndrome Clinician Rating of Ability to Communicate Choices (RTT-COMC)<br>• Rett Syndrome Clinician Rating of Verbal Communication (RTT-VCOM)<br>• Clinical Global Impression–Severity (CGI-S)<br>• Rett Syndrome Caregiver Burden Inventory (RTT-CBI) Total Score (items 1-24)<br>• Impact of Childhood Neurologic Disability (ICND) Scale Total Score
**Safety Objective**	**Safety Endpoints**
• To investigate the safety and tolerability of treatment with oral trofinetide versus placebo in girls and women with Rett syndrome	• Treatment-emergent adverse events (TEAEs)<br>• Serious adverse events (SAEs)<br>• Withdrawals due to adverse events<br>• Potentially clinically important changes in other safety assessments
**Pharmacokinetic Objectives**	**Pharmacokinetic Endpoints**
	• Whole blood concentration of trofinetide and possible metabolites

• To characterize the pharmacokinetics (PK) of trofinetide in girls and women with Rett syndrome<br>• To assess the pharmacokinetic/pharmacodynamic (PK/PD) relationship using safety and efficacy endpoints in girls and women with Rett syndrome		• Trofinetide PK parameters using the population PK approach<br>• PK/PD using appropriate PK/PD analysis methods
**Number of Study Sites**	Approximately 28 sites will participate in this study.	
**Number of Subjects Planned**	184 subjects are expected to be randomized (with a minimum of 12 subjects randomized for three age ranges [5-10 years old, 11-15 years old, and 16-20 years old]) with a total of 92 subjects per treatment arm.	
**Test Product, Dose, and Administration**	Subjects will receive an oral dose of trofinetide or placebo, for up to 12 weeks. Dose will be based on the subject's weight at Baseline, as outlined below in Table S–1. Doses may be administered by gastrostomy (G) tube (doses administered via gastrojejunal [GJ] tubes must be administered through the G-port).	

Table S–1 Dosing Schedule Based on Weight at Baseline

Weight	Dose	Total Daily Dose
12-20 kg	30 mL (6 g) BID	60 mL (12 g)
>20-35 kg	40 mL (8 g) BID	80 mL (16 g)
>35-50 kg	50 mL (10 g) BID	100 mL (20 g)
>50 kg	60 mL (12 g) BID	120 mL (24 g)

Abbreviations: BID=twice daily

If the subject cannot tolerate administration of the full assigned dose (for example, if the subject experiences diarrhea) any time before the Week 6 visit, the Investigator may instruct the caregiver to reduce study drug to a dose as low as half the assigned dose. In addition, up to four doses (in total, consecutive or non-consecutive) may be withheld within the first 6 weeks. The Investigator must attempt to increase the dose as soon as it is possible based on the clinical situation. The aim is to return to the originally assigned dose.

If the originally assigned dose cannot be reached, or the subject is again unable to tolerate that dose, the Investigator will continue treatment on the highest dose the subject can tolerate which should be no lower than half the assigned dose. The final dose must be given

	BID, once in the morning and once in the evening, unless the subject is eligible and is continuing in the open-label extension study. Subjects who continue into the extension study will take their final dose for the current study the morning of Visit 5.<br>The dose cannot be changed after the Week 6 visit.
**Coronavirus Disease 2019 (COVID-19) Update**	As a result of the COVID-19 public health emergency, this protocol has incorporated revisions to study methodology in accordance with the FDA Guidance on Conduct of Clinical Trials of Medical Products during the COVID-19 Public Health Emergency. Specifically, alternative methods of performing safety and efficacy assessments include provisions for off-site assessments. The implementation of alternative processes were to be consistent with the protocol to the extent possible, and clinical investigators were to document the reason for any contingency measures implemented.
**Study Design**	This is a 12-week, multicenter, randomized, double-blind, placebo-controlled, parallel group study. The study will compare one active treatment group receiving weight-banded doses of trofinetide with a placebo group. Subjects will be stratified according to age stratum (5-10 years old, 11-15 years old, and 16-20 years old) and Baseline RSBQ severity (<35 total score and ≥35 total score).<br>The Sponsor, subjects, caregivers, and Investigators will be blinded to treatment assignment.<br>The study will have 3 periods:<br>• Screening period: up to 3 weeks<br>• Double-blind Treatment period: 12 weeks<br>• Safety follow-up period: 30 days<br><br>**<u>Screening Period (Up to 3 Weeks)</u>**<br>During the Screening period, subjects will be assessed for study eligibility. Only those subjects who meet all inclusion and no exclusion criteria will be eligible for the study.<br>Investigators must not withdraw a subject’s prohibited medication for the purpose of enrolling them into the study. Medications will be discontinued only if it is deemed clinically appropriate to do so and in consultation with the treating physician.<br>Subjects will be evaluated for the diagnosis of Rett syndrome. In addition, there must be verified documentation of a *MECP2* mutation. Genotyping may be done as part of the study if documentation is not adequate.

	Caregivers will begin to keep a semi-structured caregiver diary during the screening period.<br>**Double-blind Treatment Period (12 Weeks)**<br>The Baseline visit (Visit 2) may occur after screening procedures are completed and have not ruled the subject out of eligibility for the study. At Visit 2, and upon confirmation of eligibility, subjects will be randomized in a 1:1 ratio to trofinetide oral solution or matching placebo. All efficacy assessments (except the CGI-I) will be completed at the Baseline visit prior to administration of the first dose of study drug (Table S–2).<br>Dose will be based on weight as outlined in Table S–1. The dose may be decreased during the study as discussed in the Test Product, Dose, and Administration section above. The first dose of study drug will be administered after all Baseline assessments are completed, or, if the Investigator judges that it is too late in the day, on the following day. The day the first dose is taken will be considered Day 1 of dosing. A triplicate ECG must be performed 2-3 hours after the first dose and a PK sample will be taken upon completion of the ECG.<br>Study drug must be discontinued at any time during the study in the event that a post-randomization QTcF duration of ≥500 ms or an increase of ≥60 ms compared to the average QTcF interval at Baseline (before dosing) is observed. For visits at which more than one ECG is completed, the average QTcF interval of all legible ECGs will be used to determine the QTcF interval for that visit.<br>Dosing is twice a day, once in the morning and once in the evening.<br>Investigational product will be shipped directly to the subject. Confirmation of any delivery to the subject will be made by a visiting nurse. Study drug shipment, return, and accountability will be performed in accordance with the drug distribution plan. In addition, study drug will be dispensed at the site during the Baseline visit when the visit is conducted in the clinic.<br>Study visits may be done off-site rather than in the clinic with the prior approval of the Sponsor or Medical Monitor. Screening, Baseline, and EOT visits should be done in the clinic whenever possible.<br>**Safety Follow-up Period (30 Days)**<br>Subjects who discontinue prematurely, or who complete but do not continue into the open-label extension study will receive a follow-up telephone call to assess safety 30 days after the last dose of study drug.<br>The study schematic is provided in Figure S–1.

	The schedule of assessments is provided in Table S–2.
**Study Duration**	The duration of participation for individual study subjects will be approximately 19 weeks, consisting of a screening period of up to 3 weeks, a treatment period of 12 weeks, and a safety follow-up period of 30 days.<br><br>The study completion date is defined as the date the final subject, across all sites, completes their final protocol-defined assessment.
**Main Criteria for Inclusion and Exclusion**	To be eligible for this study, subjects must meet all of the inclusion criteria and none of the exclusion criteria.<br><br>**Inclusion Criteria:**<br><br>1. Informed consent prior to the conduct of any study procedures is required as follows:<br>a. For subjects who are minors: written informed consent will be obtained from the legally acceptable representative (LAR). The subject should provide written or oral assent if deemed able by the Investigator. The process of obtaining informed consent will be conducted in accordance with institutional review board (IRB) or ethics committee (EC) policy and applicable local law.<br>b. For subjects who are not minors: written informed consent will be obtained from the LAR or the subject if deemed able by the Investigator. If the subject is deemed not able to provide consent, the subject should provide written or oral assent if deemed able by the Investigator. The process of obtaining informed consent will be conducted in accordance with IRB or EC policy and applicable local law.<br>c. The subject's caregiver must also provide written informed consent regarding their participation in the study prior to participating in any study procedures.<br>2. Female subjects 5 to 20 years of age, inclusive, at Screening<br>3. Body weight ≥12 kg at Screening<br>4. Can swallow the study medication provided as a liquid solution or can take it by gastrostomy tube<br>5. The subject's caregiver is English-speaking and has sufficient language skills to complete the caregiver assessments<br><br>***Diagnosis***<br><br>6. Has classic/typical Rett syndrome (RTT) (Appendix A)<br>7. Has a documented disease-causing mutation in the *MECP2* gene

	8. Is post-regression at Screening, defined as:<br>a. No loss or degradation of ambulation (including gait, coordination, independence of walking/standing) within 6 months of Screening<br>b. No loss or degradation of hand function within 6 months of Screening<br>c. No loss or degradation of speech (including babbling, words or previously developed communicative vocalizations) within 6 months of Screening<br>d. No loss or degradation of non-verbal communicative or social skills (including eye gaze, using body to indicate communicative intent, social attentiveness) within 6 months of Screening<br>9. Has a severity rating of 10-36, inclusive, on the Rett Syndrome Clinical Severity Scale at Screening<br>10. Has a CGI-S score of ≥4 at Screening and Baseline<br>***Concomitant Treatment***<br>11. If the subject is taking or was taking an anticonvulsant or any other psychoactive medication (including cannabinoids):<br>a. the treatment regimen has been stable for at least 4 weeks before Baseline and there is no current plan to change the dose, OR<br>b. if the medication was discontinued, the discontinuation has occurred no fewer than 2 weeks or 5 half-lives (whichever is greater) before Baseline<br>12. If the subject is taking or was taking any other medication daily for a chronic illness (not including antibiotics, pain relievers, and laxatives):<br>a. the treatment regimen of the medication has been stable for at least 4 weeks before Baseline and there is no current plan to change the dose, OR<br>b. if the medication was discontinued, the discontinuation has occurred no fewer than 2 weeks or 5 half-lives (whichever is greater) before Baseline<br>13. If the subject is receiving or was receiving a non-pharmacologic somatic treatment (e.g., a ketogenic diet or vagal nerve stimulation):<br>a. the treatment regimen has been stable for at least 4 weeks before Baseline and there is no current plan to change the treatment, OR<br>b. if the treatment was discontinued, the discontinuation has occurred no fewer than 2 weeks before Baseline

	14. If the subject is receiving or was receiving non-pharmacologic treatments such as an educational, behavioral, physical, occupational, or speech therapy:<br>a. the treatment regimen has been stable for at least 4 weeks before Baseline and there is no current plan to change the treatment (Note: changes to a treatment regimen that are due to school schedules or are otherwise seasonally related are not exclusionary), OR<br>b. if the treatment was discontinued, the discontinuation has occurred no fewer than 2 weeks before Baseline<br>***Seizures***<br>15. Has a stable pattern of seizures, or has had no seizures, within 8 weeks of Screening<br>***Childbearing Potential***<br>16. Subjects of childbearing potential must abstain from sexual activity for the duration of the study and for at least 30 days thereafter. If a subject is sexually active or becomes sexually active during the study, she must use 2 clinically acceptable methods of contraception (e.g., oral, intrauterine device [IUD], diaphragm plus spermicide, injectable, transdermal or implantable contraception) for the duration of the study and for at least 30 days thereafter. Subject must not be pregnant or breastfeeding.<br>***Place of Residence***<br>17. Subject and caregiver(s) must reside at a location to which study drug can be delivered and have been at their present residence for at least 3 months prior to Screening<br><br>**Exclusion Criteria:**<br>***Concomitant Treatment***<br>1. Has been treated with growth hormone within 12 weeks of Baseline<br>2. Has been treated with IGF-1 within 12 weeks of Baseline<br>3. Has been treated with insulin within 12 weeks of Baseline

	Medical Conditions Other Than Rett Syndrome<br>4. Has current clinically significant cardiovascular, endocrine (such as hypo- or hyperthyroidism, Type 1 diabetes mellitus, or uncontrolled Type 2 diabetes mellitus), renal, hepatic, respiratory or gastrointestinal disease (such as celiac disease or inflammatory bowel disease) or has major surgery planned during the study<br>5. Has a history of, or current, cerebrovascular disease or brain trauma<br>6. Has significant, uncorrected visual or uncorrected hearing impairment<br>7. Has a history of, or current, malignancy<br>***Laboratory Studies, Vital Signs, and Electrocardiogram***<br>8. Has a clinically significant abnormal laboratory value at Screening. Laboratory testing may be repeated during the Screening period with agreement of the Medical Monitor.<br>9. Has serum potassium below the normal range for the subject (according to the central laboratory) at Screening. Serum potassium may be repeated during the Screening period with the agreement of the Medical Monitor.<br>10. Has a hemoglobin A1C (HbA1c) >7.0% at Screening<br>11. Has a thyroid stimulating hormone (TSH) value outside the normal range for the subject (according to the central laboratory) at Screening<br>12. Has clinically significant abnormality in vital signs at Screening or Baseline<br>13. Has any of the following:<br>a. QTcF interval of >450 ms at Screening or Baseline (before dosing)<br>b. History of a risk factor for torsades de pointes (e.g., heart failure or family history of long QT syndrome)<br>c. History of clinically significant QT prolongation that is deemed to put the subject at increased risk of clinically significant QT prolongation<br>14. Has any other clinically significant finding on ECG at Screening or Baseline (before dosing)<br>15. Has a positive pregnancy test at Screening<br>***Other Criteria***<br>16. Has a significant sensitivity or allergic reaction to trofinetide or its excipients

	17. Has participated in another interventional clinical study within 30 days prior to Screening<br>18. Is judged by the Investigator or the Medical Monitor to be inappropriate for the study for any reason
**Pharmacokinetic Assessments**	PK blood samples will be collected at 5 timepoints for trofinetide concentration measurements at the Baseline visit (both before dosing and approximately 2-3 hours after dosing) and at Visit 3, Visit 4, and Visit 5, or upon early termination (ET).<br>The second PK sample taken at the Baseline visit (Visit 2), approximately 2-3 hours after dosing, will take place as soon as possible after the postdose ECG is performed.<br>PK samples at Visits 3, 4, and 5 should be collected at <u>one</u> of the following time intervals:<br>• 2-3 hours after dosing<br>• 4-6 hours after dosing<br>• 7-11 hours after dosing<br>Every effort should be made to collect PK samples at discrete time intervals during Visits 3, 4, and 5. However, if the interval is the same across these visits, then the collection time should vary within that interval. The following scenario is <u>only for illustrative purposes</u>, a number of other scenarios are possible.<br>• For example, if the time interval of 4-6 hours after dosing is used for Visits 3, 4, and 5, then every effort should be made to collect PK samples at 4, 5, and 6 hours after dosing, respectively.<br>Pharmacokinetic samples will also be collected, if possible, at any ET visit or the visit immediately following any SAE or following any AE leading to discontinuation.<br>For all scheduled PK samples (and for unscheduled samples if possible), the dates and times of administration of the study drug, the dates, times and content of the meals, and the dates and times of the administration of concomitant medications over the 2 days prior to and on the morning of the PK sample draw, as well as the date and time of the sample draw, will be recorded. For samples collected from subjects who experience any SAE or experience an AE leading to discontinuation, the date and time of the last dose of study drug prior to the SAE or AE leading to discontinuation will also be recorded.
**Optional Biomarker Analysis**	Participation in the effort to identify biomarkers is an optional component of the study. Subjects for whom separate informed consent for the identification of biomarkers of response to trofinetide is provided will have blood samples taken at Baseline (before dosing)

	and at Visit 5, or upon early termination. Blood samples will be used to investigate differences between responders and non-responders in both trofinetide-treated and placebo-treated subjects in RNA transcripts (transcriptomics), proteins (proteomics), and metabolites (metabolomics).
**Sample Size Calculations**	The sample size calculation was performed for the co-primary endpoints as a family of two hypothesis tests at an overall two-sided significance level of 0.05. A total sample size of 174 subjects in a 1:1 ratio to trofinetide or placebo was estimated to provide at least 90% power for the hypothesis testing family assuming the following treatment differences (SD) estimated from Phase 2 study data: -4.4 (8) for the mean change from Baseline to Week 12 in the RSBQ total score and -0.5 (0.7) for the CGI-I mean score at Week 12.<br><br>The sample size of 174 subjects will provide at least 95% power at a two-sided significance level of 0.05 for each individual hypothesis test within the family. Trofinetide will be claimed to be superior to placebo if both hypothesis tests within the family are shown to be statistically significant at 0.05. Therefore, the overall power to detect a treatment difference on both of the co-primary endpoints will be at least 90% (0.95^2).<br><br>Adjusting for an anticipated discontinuation rate of up to 5%, approximately 184 subjects will be randomized in a 1:1 ratio to trofinetide or placebo.
**Statistical Methods**	<u>**Analysis Sets**</u><br><br>The following populations will be defined and used in the analysis:<br><br><u>Safety Analysis Set</u><br><br>The Safety Analysis Set will consist of all randomized subjects who received at least one dose of study medication. The Safety Analysis Set will be analyzed according to the actual treatment received.<br><br><u>Full Analysis Set (FAS)</u><br><br>The FAS will consist of all randomized subjects who received at least one dose of study medication and who have both a Baseline value and at least one post-Baseline value for the RSBQ total score or who have at least one post-Baseline value for the CGI-I score. The FAS will be analyzed according to the treatment they were assigned regardless of the actual treatment received.<br><br><u>Per-protocol (PP) Analysis Set</u><br><br>The PP Analysis Set will consist of the subjects in FAS who did not have a major protocol violation that would affect interpretation of the

	efficacy data. The PP Analysis Set will be defined prior to study unblinding.<br><br><u>Pharmacokinetic (PK) Analysis Set</u><br><br>The PK Analysis Set will consist of subjects in the Safety Analysis Set with at least one measurable trofinetide whole blood concentration.<br><br>**<u>General Statistical Approach</u>**<br><br>Unless stated otherwise, all statistical tests will be 2-sided using a 5% significance level, leading to 95% (2-sided) confidence intervals. Trofinetide will be claimed to be superior to placebo if both co-primary endpoints are shown to be statistically significant in favor of trofinetide.<br><br>Continuous measurement results will be reported using the number of subjects with data values, mean, standard error of the mean, median, standard deviation, minimum, and maximum. For each categorical outcome, the number and percentage of subjects in each category will be reported.<br><br>A hierarchical approach will be used to control for the multiple endpoints (co-primary and secondary). Details will be provided in the SAP.<br><br>**<u>Primary Analysis</u>**<br><br>The co-primary efficacy endpoints will be analyzed using a mixed model for repeated measures (MMRM). An unstructured covariance matrix will be used and the Kenward-Roger approximation will be used to adjust the denominator degrees of freedom. The treatment comparisons will be based on the difference in least squares means at Week 12.<br><br>For the change from Baseline in the RSBQ total score, the MMRM model will include effects for treatment group, age group (5-10 years old, 11-15 years old, and 16-20 years old), Baseline RSBQ severity (<35 total score and ≥35 total score), visit, Baseline RSBQ total score, and interactions for treatment group by visit and Baseline RSBQ total score by visit.<br><br>For the CGI-I score, the MMRM model will include effects for treatment group, age group (5-10 years old, 11-15 years old, and 16-20 years old), visit, Baseline RSBQ severity (<35 total score and ≥35 total score), Baseline CGI-S score, and interactions for treatment group by visit and Baseline CGI-S score by visit.<br><br>Sensitivity analyses will be performed to assess the impact of missing data, including analyses based on a missing not at random assumption.

	Complete details of the efficacy analyses will be specified in the statistical analysis plan (SAP).<br><br>**<u>Secondary Analyses</u>**<br><br>The key secondary endpoint, change from Baseline to Week 12 in the CSBS-DP-IT Social Composite Score, will be analyzed using a MMRM method with effects for treatment group, age group (5-10 years old, 11-15 years old, and 16-20 years old), visit, Baseline RSBQ severity (<35 total score and ≥35 total score), Baseline CSBS-DP-IT Social score, and interactions for treatment group by visit and Baseline CSBS-DP-IT Social score by visit. An unstructured covariance matrix will be used and the Kenward-Roger approximation will be used to adjust the denominator degrees of freedom. The treatment comparisons will be based on the difference in least squares means at Week 12.<br><br>For the other secondary endpoints that are assessed at multiple post-Baseline visits, the change from Baseline will be analyzed using a MMRM analysis similar to those described above for the co-primary endpoints. The MMRM model will include effects for treatment group, age group (5-10 years old, 11-15 years old, and 16-20 years old), visit, Baseline RSBQ severity (<35 total score and ≥ 35 total score), Baseline score, and interactions for treatment group by visit and Baseline score by visit.<br><br>For the other secondary endpoints that are assessed at a single post-Baseline visit (i.e. Week 12 only), the change from Baseline will be analyzed using an analysis of covariance (ANCOVA) model with effects for treatment group, age group (5-10 years old, 11-15 years old, and 16-20 years old), Baseline RSBQ severity (<35 total score and ≥35 total score), and Baseline score.<br><br>**<u>Safety Analyses</u>**<br><br>Safety results will be summarized by treatment group using descriptive statistics. No formal statistical testing will be performed for any of the safety endpoints. Adverse events will be classified into standard terminology using the Medical Dictionary for Regulatory Activities (MedDRA). Treatment-emergent adverse events (TEAEs), TEAEs leading to discontinuation, TEAEs related to study drug, TEAEs by maximum severity, fatal TEAEs, serious adverse events (SAEs), and SAEs related to study drug will all be summarized.<br><br>Descriptive statistics for ECG, vital signs and weight, and clinical laboratory parameters, including changes from Baseline, will be tabulated by timepoint. Additionally, categorical analyses will be conducted on the incidence of subjects with prolonged QTc intervals and changes in QTc intervals in accordance with International Council on Harmonisation (ICH) guidelines.

	Additional safety analysis details will be specified in the SAP.<br><br>**Pharmacokinetic Analyses**<br><br>Pharmacokinetic (PK) and efficacy (i.e., PD) measures will be collected from all subjects at the Baseline (Week 0) visit before dosing, at the Baseline (Week 0) visit after dosing, and after dosing at Weeks 2, 6, and 12/EOT.<br><br>Whole blood concentration and possible metabolites data for trofinetide will be listed and summarized using descriptive statistics. If data allow, population PK and PK/PD analyses will be performed to further characterize the PK profile and exposure response relationship of trofinetide using measures of safety and efficacy parameters. Trofinetide whole blood concentration data will remain blinded until the unblinding of the clinical database at the end of the study.<br><br>The details of the PK and PK/PD analysis will be presented in a separate population PK report and PK/PD report in accordance with a separate data analysis plan (DAP).
**Date**	07 August 2020

Figure S–1 Schematic of Study Design for ACP-2566-003

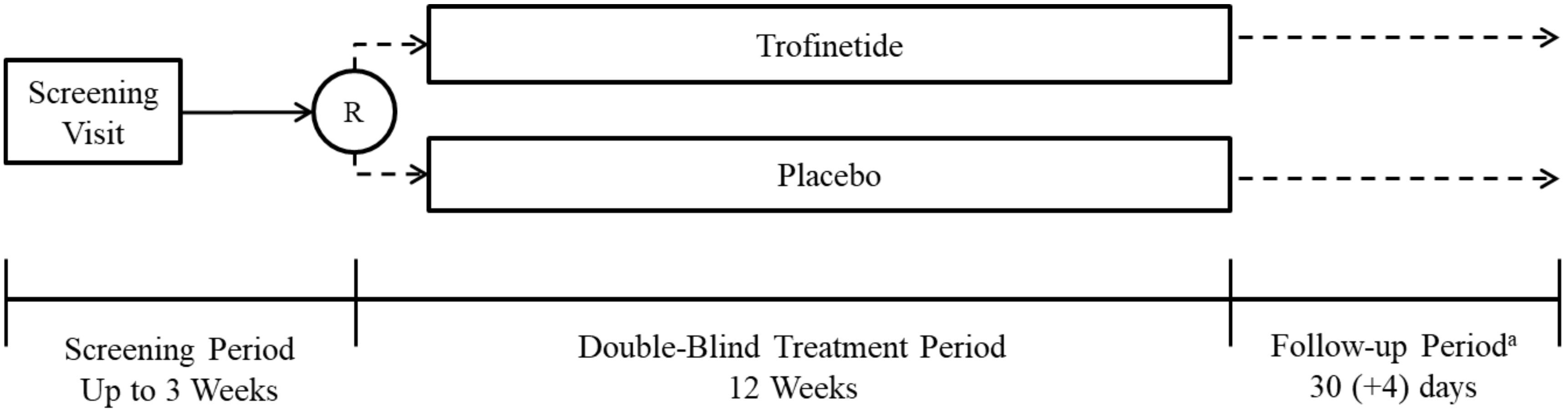

R=Randomization

[a] If the subject continues into the open-label extension (OLE) from the current study, she will not complete the follow-up visit and will roll over into the OLE

Table S–2 Schedule of Events and Assessments for Study ACP-2566-003

Period	Screening	Baseline	Double-blind Treatment Period			Safety Follow-up[b]
Visit Week		0	2[a]	6	12/EOT/ET	EOT/ET+ 30 days
Visit Number	1	2	3	4	5	
Visit window (days)	N/A	N/A	±3	±4	+3	+4
Type of Visit[k]	Clinic or Off-site					Telephone or Telemedicine
Informed consent	X				X[c]	
Inclusion/exclusion criteria	X	X				
Medical history and demographics	X					
Confirm documented Rett diagnosis and *MECP2* mutation	X					
Rett syndrome history	X					
Rett Syndrome Clinical Severity Scale	X					
Physical examination[k]	X	X	X	X	X	
Vital signs[d]	X	X	X	X	X	
Height	X				X	
Weight	X	X	X[k]	X[k]	X[k]	
12-lead electrocardiogram (ECG)[e]	X	X[e]	X	X	X	
Clinical laboratory tests (hematology, chemistry)	X	X	X	X	X	
Urinalysis	X	X	X		X	
TSH, Free T3, Free T4	X	X			X	
HbA1c	X					
Serum pregnancy test[f]	X		X	X	X	
Blood samples for pharmacokinetics		X[g]	X[h]	X[h]	X[h]	
Blood sample for optional analysis for biomarkers[i]		X			X	
Rett Syndrome Behaviour Questionnaire (RSBQ)	X	X	X	X	X	
Clinical Global Impression–Improvement (CGI-I)			X	X	X	
Clinical Global Impression–Severity (CGI-S)	X	X	X	X	X	
Communication and Symbolic Behavior Scales-Developmental Profile™ Infant-Toddler (CSBS-DP-IT) Checklist		X	X	X	X	
Rett Syndrome Clinician Rating of Hand Function (RTT-HF)		X	X	X	X	

Table continued on next page

Table S–2 Schedule of Events and Assessments for Study ACP-2566-003 (Continued)

Period	Screening	Baseline	Double-blind Treatment Period			Safety Follow-up[b]
Visit Week		0	2[a]	6	12/EOT/ET	EOT/ET+ 30 days
Visit Number	1	2	3	4	5	
Visit window (days)	N/A	N/A	±3	±4	+3	+4
Type of Visit[k]	Clinic or Off-site					Telephone or Telemedicine
Rett Syndrome Clinician Rating of Ability to Communicate Choices (RTT-COMC)[k]		X	X	X	X	
Rett Syndrome Clinician Rating of Ambulation and Gross Motor Skills (RTT-AMB)		X	X	X	X	
Rett Syndrome Clinician Rating of Verbal Communication (RTT-VCOM)		X	X	X	X	
Rett Syndrome Caregiver Burden Inventory (RTT-CBI)		X			X	
Impact of Childhood Neurologic Disability Scale (ICND)		X			X	
Dispensing and review of semi-structured caregiver diary	X	X	X	X	X	
Concomitant medications	X	X	X	X	X	X
Assessment of adverse events	X	X	X	X	X	X
Randomization		X				
Study drug dispensed[j]		X				
Authorization of study drug dispensation[j]		X-----------------------X				
Study drug return[j]			X-----------------------------X			
Study drug accountability[j]			X	X	X	

Abbreviations: EOT=end of treatment; ET=early termination; HbA1c=glycosylated hemoglobin; *MECP2*=methyl-CpG-binding protein 2 gene; TSH=thyroid stimulating hormone

[a] Timing of post-Baseline visits will be calculated from the first day of dosing (Day 1) (i.e. the Week 2 visit will occur 2 weeks [±3 days] after the first day of dosing).

[b] Subjects who roll over into the OLE study will not have the safety follow-up telephone call.

[c] For subjects who decide to continue into the open-label extension (OLE) study, informed consent for the OLE **must be** obtained prior to performing the Week 12/EOT procedures.

[d] Vital signs will include body temperature, resting respiration rate, sitting systolic and diastolic blood pressure, and pulse rate. The sitting blood pressure will be measured after the subject has been sitting for ≥3 minutes.

[e] ECGs will be completed in triplicate at Visit 1 (Screening), at Visit 2 (Baseline) both before dosing and 2-3 hours after dosing, and at Visit 5 (Week 12/EOT/ET). A single ECG will be completed at Visit 3 (Week 2) and Visit 4 (Week 6).

[f] For subjects who have reached menarche and have not had surgical sterilization.

[g] A predose PK blood sample must be collected before administration of study drug. A postdose PK blood sample will be collected at the end of ECG assessment 2-3 hours after study drug administration.

[h] PK samples at Visits 3, 4, and 5 will be collected at one of the following time intervals: 1) 2-3 hours after dosing OR 2) 4-6 hours after dosing OR 3) 7-11 hours after dosing. Every effort should be made to collect the PK samples at discrete time intervals during Visits 3, 4, and 5. However, if the interval is the same across these visits, then the collection time should vary within that interval.

[i] Participation in the effort to identify biomarkers is an optional component of the study requiring a separate informed consent, which may be obtained at any time during the study. If consent is obtained after Baseline, only the sample at Visit 5 (or upon early termination) will be taken.

[j] Investigational product will be shipped directly to the subject. Confirmation of any delivery to the subject will be made by a visiting nurse. Study drug shipment, return, and accountability will be performed in accordance with the drug distribution plan. In addition, study drug will be dispensed at the site during the Baseline visit when the visit is conducted in the clinic.

[k] Study visits may be done off-site rather than in the clinic with the prior approval of the Sponsor or Medical Monitor. Screening, Baseline, and EOT visits should be done in the clinic whenever possible. When a study visit takes place off-site, the physical examination will not be required. Weight should be measured whenever possible at off-site visits. The RTT-COMC should be completed if possible, but it is not required.

TABLE OF CONTENTS

SPONSOR SIGNATURE PAGE 2
DECLARATION OF INVESTIGATOR 4
PROTOCOL SYNOPSIS 5
TABLE OF CONTENTS 23
LIST OF TABLES 28
LIST OF FIGURES 28
LIST OF ABBREVIATIONS AND DEFINITION OF TERMS 29
1 INTRODUCTION 31
1.1 Background Information 31
1.2 Investigational Product 32
1.3 Previous Clinical Experience 33
1.4 Study Rationale 36
2 STUDY OBJECTIVES AND ENDPOINTS 37
2.1 Primary Objective 37
2.1.1 Co-Primary Endpoints 37
2.2 Key Secondary Objective 38
2.2.1 Key Secondary Endpoint 38
2.3 Other Secondary Objectives 38
2.3.1 Other Secondary Endpoints 38
2.4 Safety Objective 39
2.4.1 Safety Endpoints 39
2.5 Pharmacokinetic Objectives 39
2.5.1 Pharmacokinetic Endpoints 39
3 STUDY DESCRIPTION 39
3.1 Overview of Study Design 39
3.1.1 Screening Period (Up to 3 Weeks) 40
3.1.2 Double-Blind Treatment Period (12 Weeks) 41
3.1.3 Safety Follow-Up Period (30 Days) 42
4 SUBJECT ELIGIBILITY AND WITHDRAWAL CRITERIA 42
4.1 Inclusion Criteria 42
4.2 Exclusion Criteria 44

4.3 Subject Withdrawal of Consent 45
4.4 Subject or Study Discontinuation 46
4.4.1 Post-Baseline QTcF Interval Stopping Criteria 46
4.4.2 Handling of Subject Discontinuation During the Treatment Period 47
4.5 Subject Lost to Follow-Up 47
4.6 Prior and Concomitant Therapy 47
4.6.1 Permitted and Prohibited Medications 48
5 INVESTIGATIONAL PRODUCT 49
5.1 Investigational Product Description 49
5.1.1 Formulation, Appearance, and Packaging 49
5.1.2 Product Storage and Stability 49
5.1.3 Dosing and Administration 49
5.1.3.1 Dosing 49
5.1.3.2 Administration of Study Drug 51
5.1.4 Method of Assigning Subjects to Treatment Groups 51
5.1.5 Blinding 51
5.1.6 Study Drug Compliance 52
5.1.7 Overdose 52
5.2 Investigational Product Accountability Procedures 52
6 STUDY PROCEDURES 52
6.1 Screening Assessments 53
6.1.1 Confirm Diagnosis of Rett Syndrome and *MECP2* Mutation 53
6.1.2 Medical History, Including Rett Syndrome History, and Demographics 53
6.1.3 The Rett Syndrome Clinical Severity Scale (RTT-CSS) 53
6.2 Efficacy Assessments 54
6.2.1 Rett Syndrome Behaviour Questionnaire (RSBQ) 54
6.2.2 Clinical Global Impression–Improvement (CGI-I) and Clinical Global Impression–Severity (CGI-S) 55
6.2.3 Communication and Symbolic Behavior Scales Developmental Profile Infant-Toddler (CSBS-DP-IT) Checklist 55
6.2.4 Impact of Childhood Neurologic Disability (ICND) Scale 56
6.2.5 Rett Syndrome Clinician Rating of Hand Function (RTT-HF) 57
6.2.6 Rett Syndrome Clinician Rating of Ambulation and Gross Motor Skills (RTT-AMB) 57
6.2.7 Rett Syndrome Clinician Rating of Ability to Communicate Choices (RTT-COMC) 57
6.2.8 Rett Syndrome Clinician Rating of Verbal Communication (RTT-VCOM) 57

6.2.9 Rett Syndrome Caregiver Burden Inventory (RTT-CBI) 58
6.3 Safety Assessments....58
6.3.1 Physical Examination 58
6.3.2 Vital Signs 58
6.3.3 Height, Weight, and Body Mass Index....59
6.3.4 Electrocardiograms....59
6.3.4.1 Post-Baseline QTcF Interval Stopping Criteria....60
6.3.5 Laboratory Evaluations 60
6.4 Caregiver Diary 61
6.5 Pharmacokinetic Assessments....61
6.5.1 Specimen Preparation, Handling, Storage, and Shipment....63
6.6 Identification of Biomarkers of Response to Trofinetide in Rett Syndrome....63
7 ADVERSE EVENTS 64
7.1 Specification of Safety Parameters....64
7.1.1 Definition of Adverse Event....64
7.1.2 Definition of Serious Adverse Event....65
7.2 Classification of an Adverse Event 66
7.2.1 Severity of Event....66
7.2.2 Relationship to Study Drug 66
7.2.2.1 Duration....67
7.2.2.2 Frequency 67
7.2.2.3 Action Taken with Study Drug....67
7.2.2.4 Therapy....67
7.2.2.5 Outcome 68
7.2.2.6 Seriousness 68
7.2.3 Definition of Unexpectedness 68
7.3 Time Period and Frequency for Event Assessment and Follow-Up....68
7.3.1 Adverse Event Reporting 68
7.3.2 Serious Adverse Event Reporting....69
7.3.3 Reporting of Pregnancy....70
7.3.3.1 Reporting Paternal Drug Exposure....70
7.3.4 Reporting of Overdose 70
8 CLINICAL MONITORING....70
9 STATISTICAL METHODS AND DATA ANALYSIS 71
9.1 Statistical and Analytical Plans 71
9.2 Statistical Hypotheses....71

9.3 Sample Size Determination 71
9.4 Subject Populations for Analysis 71
9.5 Statistical Analyses 72
9.5.1 General Approach 72
9.5.2 Primary Analyses 72
9.5.3 Secondary Analyses 73
9.5.4 Safety Analyses 73
9.5.5 Pharmacokinetic Analyses 74
9.5.6 Subgroup Analyses 74
9.6 Interim Analyses 74
9.7 Data and Safety Monitoring Board 74
9.8 Measures to Minimize Bias 74
9.9 Breaking the Study Blind/Subject Code 75
10 STUDY MANAGEMENT AND DATA COLLECTION 75
10.1 Data Collection and Management Responsibilities 75
10.2 Source Documents 76
10.3 Case Report Forms 76
10.4 Confidentiality 76
10.5 Study Records Retention 76
10.6 Protocol Exceptions and Deviations 77
10.7 Protocol Amendments 77
11 STUDY MONITORING, AUDITING, AND INSPECTING 77
11.1 Quality Control and Quality Assurance 77
11.2 Risk Management 78
12 ETHICAL CONSIDERATIONS 79
12.1 Ethical Standard 79
12.2 Institutional Review Board/Ethics Committee 79
12.3 Informed Consent Process 79
12.3.1 Consent and Other Informational Documents Provided to Subjects 80
12.3.2 Consent Procedures and Documentation 80
13 PUBLICATION PLAN 81
14 CONFLICT OF INTEREST POLICY 81
14.1 Finance, Insurance, and Indemnity 81

15 LITERATURE REFERENCES....................82

16 APPENDICES....................87

Appendix A Rett Syndrome Diagnostic Criteria....................87

LIST OF TABLES

Table S–1 Dosing Schedule Based on Weight at Baseline 7
Table S–2 Schedule of Events and Assessments for Study ACP-2566-003 20
Table 1–1 Change from Treatment Baseline (Day 14) to End of Treatment (Day 54) in Core Efficacy Outcomes (mITT) (Neu-2566-RETT-002) 34
Table 5-1 Dosing Schedule Based on Weight at Baseline 50
Table 6–1 PK Sampling Times 63

LIST OF FIGURES

Figure S–1 Schematic of Study Design for ACP-2566-003 19
Figure 1–1 Comparison of Change from Treatment Baseline on the RSBQ at Follow-Up Assessments Between 200 mg/kg BID and Placebo BID Treatment Groups 35

LIST OF ABBREVIATIONS AND DEFINITION OF TERMS

Term	Definition
AE	adverse event(s)
BID	bis in die; twice daily
CGI-I	Clinical Global Impression–Improvement
CGI-S	Clinical Global Impression–Severity
CSBS-DP-IT	Communication and Symbolic Behavior Scales-Developmental Profile™ Infant-Toddler
DSMB	Data and Safety Monitoring Board
EC	ethics committee
ECG	electrocardiogram
eCRF	electronic case report form
EOT	end of treatment
ET	early termination
GCP	Good Clinical Practice
GJ	gastrojejunal
GPE	glycine-proline-glutamate
HbA1c	glycosylated hemoglobin
ICF	informed consent form
ICH	International Council for Harmonisation
ICND	Impact of Childhood Neurologic Disability Scale
IGF-1	insulin-like growth factor 1
IRB	institutional review board
IRT	interactive response technology
LAR	legally acceptable representative
MeCP2	methyl-CpG binding protein 2
MeCP2	gene encoding methyl-CpG binding protein 2 (in animals)
MECP2	gene encoding methyl-CpG binding protein 2 (in humans)
MMRM	mixed model for repeated measures
PD	pharmacodynamic
PK	pharmacokinetic(s)
PR interval	PR interval of ECG
QRS interval	QRS interval of ECG
QT interval	QT interval for heart rate of ECG
QTc	corrected QT interval of ECG for heart rate
QTcB	corrected QT interval using Bazett's correction method
QTcF	corrected QT interval using Fridericia's correction method

Term	Definition
RSBQ	Rett Syndrome Behaviour Questionnaire
RTT	Rett syndrome
RTT-AMB	Rett Syndrome Clinician Rating of Ambulation and Gross Motor Skills
RTT-CBI	Rett Syndrome Caregiver Burden Inventory
RTT-COMC	Rett Syndrome Clinician Rating of Ability to Communicate Choices
RTT-CSS	Rett Syndrome Clinical Severity Scale
RTT-DSC	RTT Domain Specific Visual Analog Scale
RTT-HF	Rett Syndrome Clinician Rating of Hand Function
RTT-VCOM	Rett Syndrome Clinician Rating of Verbal Communication
SAE	serious adverse event(s)
TEAE	treatment emergent adverse event(s)
US	United States

1 INTRODUCTION

This document is a research protocol and the described study will be conducted in compliance with the protocol and the International Council for Harmonisation (ICH) Good Clinical Practice (GCP) Guideline.

1.1 Background Information

Rett syndrome (RTT) is a seriously debilitating neurodevelopmental disorder for which there is currently no approved treatment. Its prevalence is reported as 1 in 10,000-15,000 female live births (Bienvenu et al. 2006; Neul et al. 2010). While the great majority of patients with RTT are females, males who meet the criteria for RTT have also been identified (Neul et al. 2019). In 96-98% of patients diagnosed with classic RTT, the disease is caused by mutations in the X-linked *MECP2* gene (Percy et al. 2018). *MECP2* encodes methyl-CpG binding protein 2 (MeCP2) which modulates gene expression by binding to methylated CpG dinucleotides, primarily by activating but also by repressing transcription (Ip et al. 2018; Neul et al. 2008; Kriaucionis et al. 2003; Hite et al. 2009). The activity of the MeCP2 protein is diminished in both neurons and astrocytes (Yasui et al 2013).

In patients with typical RTT, there is seemingly normal psychomotor development for the first six months of life, but soon thereafter the failure to reach normal developmental milestones is observed, followed by a period of developmental regression in which there is a loss of normal use of the hands and of spoken language (Samaco and Neul 2011). The period of developmental regression is accompanied by transient autistic features in many but not all individuals with RTT (Lee et al. 2013; Neul et al. 2014). Loss of social skills appears to stabilize or reverse after the regression period, and some individuals with RTT demonstrate social intentions through eye contact (Young et al. 2008; Djukic and McDermott 2012; Urbanowicz et al. 2016). Nonetheless, social interaction and communication remain limited (Mount et al. 2003; Urbanowicz et al. 2015; Rose et al. 2013; Kaufmann et al. 2012; Woodyatt and Ozanne 1993). The intellectual disability in RTT appears to be profound; however, precise measurement of the extent of cognitive impairment is difficult because of the severe communication and motor deficits affecting most individuals (Byiers and Symons 2012; Clarkson et al. 2017).

Seizures are common, although not diagnostic. Commonly observed symptoms include awake breathing disruptions, scoliosis, and interest in social interaction (intense eye communication) (Neul et al. 2010; Percy et al. 2010). Gastrointestinal symptoms, including constipation and chewing and swallowing difficulties are observed in the majority of patients with RTT (Motil et al. 2012). Sudden changes in mood, screaming and inconsolable crying are common behaviors in children and adolescents with RTT (Mount et al. 2001, 2002; Robertson et al. 2006; Cianfaglione et al. 2015). These impairments can further exacerbate

other symptoms and disrupt activities of daily living including educational, recreational and treatment opportunities (Epstein et al. 2016; Perry et al. 1991; Thompson and Iwata 2001; Iemmi et al. 2015). Autonomic manifestations, which include abnormalities in cardiac and respiratory function, as well as in peripheral circulation, are considered to be predominantly of CNS origin. RTT is also characterized by impaired growth affecting the brain and other organ systems.

Loss of purposeful hand use is a defining aspect of RTT during the onset of regression and at least 30% of individuals remain without any type of purposeful hand use (Downs et al. 2010). Individuals with some purposeful hand movements vary in their level of ability to reach for and grasp objects. The ability to self-feed is observed in 25-43% (Cass et al. 2003; Larsson et al. 2005; Downs et al. 2011). Poorer levels of hand function are associated with both age and severity of mobility impairment (Downs et al. 2011).

In adulthood a late motor deterioration stage occurs, characterized by worsening dystonia, rigidity, and in some cases deterioration in the ability to walk and parkinsonian symptoms. Affected individuals have a yearly death rate between 1 and 2%, with 25% of all deaths characterized as sudden and unexpected (Kerr et al. 1997; Samaco and Neul 2011). Women with RTT can survive into their fifties and occasionally longer (Samaco and Neul 2011).

There are no medicines approved for the treatment of Rett syndrome. Treatment focuses on the management of each patient's symptoms and, even in that regard, is often unsatisfactory and has only a limited effect on functional improvement. Accordingly, the burden on families and other caregivers is very great (Palacios-Ceña et al. 2018).

1.2 Investigational Product

Trofinetide is a synthetic analog of glycine-proline-glutamate (also known as glypromate or GPE), a peptide that occurs naturally in the brain. GPE is the n-terminal tripeptide of the insulin-like growth factor 1 (IGF-1) protein. Trofinetide crosses the blood-brain barrier following oral administration. In the brain, it is believed to normalize decreased bioavailability of IGF-1 and GPE, as well as having an anti-inflammatory effect on pathologically activated glial cells. Both conditions contribute to deficits in synaptic development and functional maturation of synaptic plasticity that are fundamental to the wide-ranging effects of RTT. Tropea et al. (2009) observed that treatment with GPE reversed Rett-like symptoms in *MeCP2* mutant mice. Therefore, the aim of treatment with trofinetide is to exert an effect on brain structure and function such as dendritic length and branching and long-term potentiation, which would be expected to lead to improvements across a wide range of symptoms of RTT.

1.3 Previous Clinical Experience

Trofinetide showed linear pharmacokinetics across the dose range tested in pediatric RTT patients. These pharmacokinetic results are in agreement with the data obtained previously in healthy subjects and in adult RTT patients. From a drug metabolism perspective, there was no accumulation, metabolic inhibition, or induction observed during treatment. For subjects treated with 50 mg/kg BID, median C_{max} was 17.7 µg/mL and median $AUC_{(0-12ss)}$ was 139.4 µg/mL·h. For subjects treated with 100 mg/kg BID, median C_{max} was 52.6 µg/mL and median $AUC_{(0-12ss)}$ was 338.6 µg/mL·h. For subjects treated with 200 mg/kg BID, median C_{max} was 82.2 µg/mL and median $AUC_{(0-12ss)}$ was 505.1 µg/mL·h. The geometric mean of the apparent terminal elimination half-life ($T_{1/2}$) varied from 5.3 hr to 6.1 hr across the three dosing groups.

Trofinetide has exhibited a favorable safety and tolerability profile in two studies in subjects with Rett syndrome. Study Neu-2566-RETT-001 (NCT01703533) was a Phase 2 study of trofinetide in 56 adolescent and adult females with RTT (Glaze et al. 2017). Subjects were randomized 2:1 to 35 mg/kg trofinetide BID or placebo for 14 or 28 days (Cohorts 0 and 1, respectively) or 70 mg/kg trofinetide BID or placebo for 28 days (Cohort 2). Both dose levels were well-tolerated in this study, and no time- or dose-dependent adverse events (AEs) were apparent. The most common AE across all treatment groups was diarrhea (39% in the combined 35 mg/kg BID group, 11% in the 70 mg/kg BID group and 15% in the placebo group). The most common AE in the 70 mg/kg BID group was somnolence (17%; versus 5% in the placebo group and 0% in the 35 mg/kg BID group). Four serious adverse events (SAEs) involving three subjects were reported during the study. In each of these instances, the adverse events were deemed unrelated to the study medication. One subject withdrew from the study due to these events while two subjects had already finished the study medication when their SAEs occurred. Each of these SAEs was deemed to be resolved by study conclusion. The results of this study also provided preliminary evidence of a treatment effect in the 70 mg/kg BID treatment group.

A second Phase 2 study of oral trofinetide in pediatric and adolescent females with RTT, Study Neu-2566-RETT-002, assessed three dose levels of trofinetide (50 mg/kg BID, 100 mg/kg BID, and 200 mg/kg BID versus placebo) (Glaze et al. 2019). The study population consisted of female subjects between 5 and 15 years of age with a diagnosis of RTT and a proven mutation of the *MECP2* gene. The primary analysis of efficacy was change from Treatment Baseline (Visit 3, Day 14, end of placebo run-in) to End-of-Assessment (Visit 7, Day 54) between placebo and the 200 mg/kg BID treatment groups.

At the time the study was initiated, there was no consensus upon the most appropriate and sensitive endpoint(s) for detecting beneficial drug effect in RTT, so the study examined

five unranked core efficacy outcome measures. Each of these five endpoints represented a different dimension or manifestation of the pathophysiology and clinical impairment directly associated with RTT. As shown below in Table 1–1, three of the five core endpoints showed a statistically significant difference from placebo at $p<0.05$ for the 200 mg/kg BID dose group: two clinician completed assessments (RTT Domain Specific Visual Analog Scale [RTT-DSC] and Clinical Global Impression–Improvement [CGI-I]) and one of the caregiver completed assessments (RSBQ). The remaining two primary endpoints were directionally positive.

Table 1–1 Change from Treatment Baseline (Day 14) to End of Treatment (Day 54) in Core Efficacy Outcomes (mITT) (Neu-2566-RETT-002)

Outcome Measure	Prespecified covariates p≤0.1	Placebo (n=24)	50 mg/kg (n=15)	100 mg/kg (n=16)	200 mg/kg (n=27)
**RSBQ Total**<br>D14 Treatment Baseline<br>Change D14-D54 (LS mean)<br>p-value vs. placebo[a]	PR	39.5<br>-2.3	44.7<br>-3.0<br>0.768	40.3<br>-1.5<br>0.749	42.2<br>-6.7<br>**0.042**
**RTT-DSC Total**<br>***Exact Median Test***<br>D14 Treatment Baseline<br>Change D14-D54<br>p-value vs. placebo[a]	UnAdj	473.3<br>-25.85	450.0<br>-32.50<br>0.999	445.3<br>-12.10<br>0.748	516.6<br>-76.00<br>**0.025**
**CGI-I**<br>D14 Treatment Baseline<br>Change D14-D54 (LS mean)<br>p-value vs. placebo[b]	UnAdj	(4)<br>3.5	(4)<br>3.3<br>0.391	(4)<br>3.4<br>0.703	(4)<br>3.0<br>**0.029**
**Top 3 Caregiver Concerns**<br>D14 Treatment Baseline<br>Change D14-D54 (LS mean)<br>p-value vs. placebo[a]	UnAdj	223.8<br>-12.52	237.6<br>-16.56<br>0.776	211.5<br>-2.09<br>0.455	245.9<br>-18.54<br>0.619
**MBA Total**<br>D14 Treatment Baseline<br>Change D14-D54 (LS mean)<br>p-value vs placebo[a]	TBL, PR	48.8<br>-2.6	46.6<br>-2.8<br>0.872	48.6<br>-2.4<br>0.925	46.6<br>-2.9<br>0.840

Abbreviations: CGI-I=Clinical Global Impression–Improvement; LS =least squares; MBA=Motor Behavior Assessment; mITT=modified Intent-to-treat; PR=placebo response; RSBQ=Rett Syndrome Behaviour Questionaire; RTT-DSC=RTT Domain Specific Visual Analog Scale; TBL=treatment baseline; UnAdj=unadjusted

a Prespecified model covariates if p≤0.1: TBL and/or PR.

b CGI-I has no pretreatment baseline value. The CGI-I values at Day 14 are the ratings of change from Day 0 (the pretreatment baseline) to Day 14 (end of placebo run-in).

Among the three outcome measures that exhibited improvement compared to placebo for the 200 mg/kg BID group, the median magnitude of effect (change) was 15% for RTT-DSC (vs. 5% for placebo) and the mean magnitude of effect (change) was 16% for RSBQ (vs. 6%

for placebo). For the CGI-I, the mean outcome was 3.0 for the 200 mg/kg BID group and was 3.5 for placebo. For the CGI-I, more than 20% of subjects in the 200 mg/kg BID group were judged by clinicians to be "much improved", compared with less than 5% of those on placebo.

At the end of the dosing period, data indicate that clinical benefit (change from treatment baseline) was continuing to accrue and that there was a divergent trend between the placebo and 200 mg/kg BID groups as highlighted below in Figure 1–1 for the RSBQ. Further, there is evidence of a significant diminution of effect following cessation of dosing, suggesting that the separation during the dosing period was due to beneficial treatment effect and not from other differences between the two arms that occurred simply by chance.

Figure 1–1 Comparison of Change from Treatment Baseline on the RSBQ at Follow-Up Assessments Between 200 mg/kg BID and Placebo BID Treatment Groups

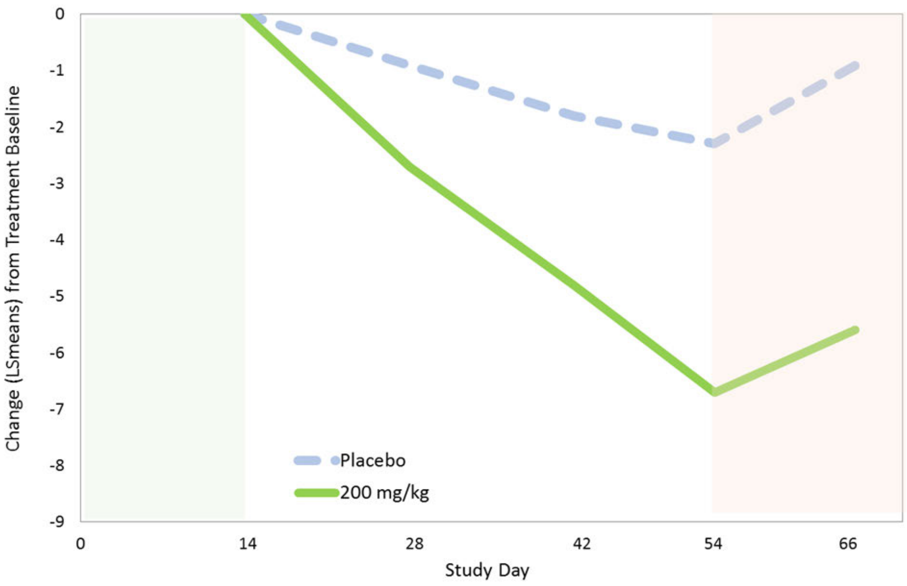

Abbreviations: BID=twice daily; LS=least squares; RSBQ=Rett Syndrome Behaviour Questionaire

Note: End of treatment assessment is at Day 54. Treatment baseline is at Day 14, at the end of the placebo run-in. Day 66 is post-treatment.

Trofinetide was generally safe and well tolerated. Most AEs reported during the double-blind treatment period were mild in intensity and the majority of events were deemed not related to study medication. The most common AEs reported from the start of the double-blind period across all treatment groups were diarrhea (27%), vomiting (15%), upper respiratory tract pyrexia (13%), and upper respiratory tract infection (12%). The most common AE from the start of the double-blind treatment period in the trofinetide treatment groups was diarrhea (27% in the 50 mg/kg BID group, 13% in the 100 mg/kg BID group, and 56% in the

200 mg/kg BID group compared with 4% in the placebo group). The diarrhea associated with trofinetide may have been due to an improvement in gastrointestinal motility added to ongoing treatment with laxatives. Only three subjects experienced SAEs: one subject in the placebo BID group had an SAE of pneumonia, one subject in the 100 mg/kg BID group had an SAE of worsening of tonic cluster seizures, and one subject in the 200 mg/kg BID group had SAEs of strep infection and pneumonia. In each of these instances, the SAEs were deemed not related to study medication and resolved by the end of the study.

No changes in QTc interval attributable to treatment were seen in adolescent and adult subjects with RTT in study Neu-2566-RETT-001, nor in pediatric subjects in Neu-2566-RETT-002.

Phase 2 studies of the oral formulation of trofinetide in adolescent and adult subjects with Fragile X syndrome and of the intravenous formulation in adult subjects with moderate to severe traumatic brain injury (TBI) have also been completed and are described in the Investigator's brochure.

Always refer to the latest version of the trofinetide Investigator's Brochure for the overall benefit/risk assessment and the most accurate and current information regarding drug metabolism, pharmacokinetics, efficacy, and safety.

1.4 Study Rationale

As described above, Rett syndrome is a devastating disorder for which there is as yet no treatment beyond symptomatic care resulting in a great unmet medical need. In the two studies of trofinetide in girls and women with RTT, trofinetide has been well tolerated. The highest dose assessed, 200 mg/kg BID, was observed to provide benefit compared to placebo even in a relatively small study, as described above. The rationale for the present study is to assess whether treatment with trofinetide does show a statistically significant benefit compared to placebo in a well-powered study.

Accordingly, the present study is designed to assess whether the effects of administration of trofinetide seen in the two Phase 2 studies, are confirmed in a larger population of children, adolescents and young adults.

This study will investigate effects in females only, and only in subjects who have typical RTT and a confirmed mutation of the *MECP2* gene. The enrollees will be in a stable phase of their RTT; that is, they will not be undergoing active neurological regression. The exclusion of subjects with atypical RTT, subjects without a documented disease-causing *MECP2* mutation, and male subjects is based on considerations of study design that will allow for a more homogeneous study population and, it is hoped, reduce variability that will allow

observation of a difference between treatment with trofinetide and treatment with placebo in this relatively small sample of the population.

The upper limit of the allowed age range has been increased to 20 years of age as compared to 15 years of age in the most recent Phase 2 study. The age range is not over 20 years in large part because in the United States school districts generally provide services until 20 or 21 years of age and the availability of services after that age is not consistent across the states.

The study will compare the efficacy and safety of a single trofinetide treatment group to treatment with placebo. Based on the results of the previous study treatment with trofinetide, 200 mg/kg BID was targeted. However, it was observed that subjects with lower body weights tended to have lower than expected exposure than did subjects who weighed more. Identification of body weight as a covariate has often been observed with drugs showing similar distributional properties, especially when considering adolescents or other groups with different demographic characteristics (Jusko et al. 1982; Kersting et al. 2012; Piana et al. 2014). A best practice approach to addressing this covariate is the development of a dosing model that accounts for the effect of weight on exposure. A model of simulated dose levels was developed based on the Neu-2566-RETT-002 data to characterize the dose levels required to reach a minimal target exposure level of orally administered trofinetide at different weights in a pediatric population. The dose simulation modeling showed that a four-level model of weight dosing bands with fixed doses of 6 g BID, 8 g BID, 10 g BID or 12 g BID (Table 5-1) would result in an optimal percentage of subjects with exposures within the target range at weights 12 kg-100 kg.

2 STUDY OBJECTIVES AND ENDPOINTS

2.1 Primary Objective

The primary objective of this study is to investigate the efficacy of treatment with oral trofinetide versus placebo in girls and women with Rett syndrome.

2.1.1 Co-Primary Endpoints

There are 2 primary endpoints. The co-primary endpoints are:

- Rett Syndrome Behaviour Questionnaire (RSBQ) total score – change from Baseline to Week 12
- Clinical Global Impression–Improvement (CGI-I) score at Week 12

2.2 Key Secondary Objective

The key secondary objective is to investigate the efficacy of treatment with oral trofinetide versus placebo on ability to communicate in girls and women with Rett syndrome.

2.2.1 Key Secondary Endpoint

The key secondary endpoint is change from Baseline to Week 12 in Communication and Symbolic Behavior Scales Developmental Profile™ Infant-Toddler Checklist – Social Composite Score (CSBS-DP-IT Social).

2.3 Other Secondary Objectives

Additional secondary objectives for this study are:

- To investigate the benefit of treatment with oral trofinetide versus placebo on overall quality of life for girls and women with Rett syndrome
- To investigate the efficacy of treatment with oral trofinetide versus placebo in girls and women with Rett syndrome on:
    - Hand function
    - Ambulation and other gross motor skills
    - Ability to communicate choices and preferences
    - Ability to communicate verbally
- To investigate the efficacy of treatment with oral trofinetide versus placebo on a global assessment of the severity of illness in girls and women with Rett syndrome
- To investigate the benefit of treatment with oral trofinetide versus placebo on the burden on caregivers of girls and women with Rett syndrome
- To investigate the benefit of treatment with oral trofinetide versus placebo on the impact of the disability on the child's and family's everyday life

2.3.1 Other Secondary Endpoints

The secondary endpoints for this study are change from Baseline to Week 12 in:

- Overall Quality of Life Rating of the Impact of Childhood Neurologic Disability (ICND) Scale
- Rett Syndrome Clinician Rating of Hand Function (RTT-HF)
- Rett Syndrome Clinician Rating of Ambulation and Gross Motor Skills (RTT-AMB)
- Rett Syndrome Clinician Rating of Ability to Communicate Choices (RTT-COMC)

- Rett Syndrome Clinician Rating of Verbal Communication (RTT-VCOM)
- Clinical Global Impression–Severity (CGI-S)
- Rett Syndrome Caregiver Burden Inventory (RTT-CBI) Total Score (items 1-24)
- Impact of Childhood Neurologic Disability (ICND) Scale Total Score

2.4 Safety Objective

The safety objective of this study is to investigate the safety and tolerability of treatment with oral trofinetide versus placebo in girls and women with Rett syndrome.

2.4.1 Safety Endpoints

The safety endpoints of this study are:

- Treatment-emergent adverse events (TEAEs)
- Serious adverse events (SAEs)
- Withdrawals due to AEs
- Potentially clinically important changes in other safety assessments

2.5 Pharmacokinetic Objectives

The pharmacokinetic objectives of this study are:

- To characterize the pharmacokinetics (PK) of trofinetide in girls and women with Rett syndrome
- To assess the pharmacokinetic/pharmacodynamic (PK/PD) relationship using safety and efficacy endpoints in girls and women with Rett syndrome

2.5.1 Pharmacokinetic Endpoints

The pharmacokinetic endpoints for this study are:

- Whole blood concentration of trofinetide and possible metabolites
- Trofinetide PK parameters using the population PK approach
- PK/PD using appropriate PK/PD analysis methods

3 STUDY DESCRIPTION

3.1 Overview of Study Design

This study will be conducted as a multicenter, randomized, double-blind, placebo-controlled, parallel-group study in girls and women with Rett syndrome. The study will compare one active treatment group receiving weight-banded doses of trofinetide (Table 5-1) with a

placebo group. Subjects will be stratified according to age stratum (5-10 years old, 11-15 years old, and 16-20 years old) and Baseline RSBQ severity (<35 total score and ≥35 total score). A minimum of 12 subjects are required to be randomized for each age stratum. The Sponsor, subjects, caregivers, and Investigators will be blinded to treatment assignment.

The duration of participation for individual study subjects will be approximately 19 weeks. Approximately 28 sites will participate in this study.

The study will have 3 periods (Figure S–1):

- Screening period: up to 3 weeks
- Double-blind Treatment period: 12 weeks
- Safety follow-up period: 30 days

The schedule of events and assessments is provided in Table S–2. As shown in the schedule of events, study visits may be done off-site rather than in the clinic with the prior approval of the Sponsor or Medical Monitor. Screening, Baseline, and EOT visits should be done in the clinic whenever possible. Approval for off-site visits at Screening, Baseline, and EOT will be given only under special circumstances.

The study completion date is defined as the date the final subject, across all sites, completes their final protocol-defined assessment. An individual subject is considered to have completed the study on the date of her final protocol-defined assessment. Please note that the 'final protocol-defined assessment' includes the follow-up visit or contact, whichever is later. Procedures for when a subject is lost to follow-up are provided in Section 4.5.

3.1.1 Screening Period (Up to 3 Weeks)

During the Screening period, subjects will be assessed for study eligibility. Only those subjects who meet all inclusion and no exclusion criteria will be eligible for the study.

Investigators must not withdraw a subject's prohibited medication for the purpose of enrolling them into the study. Medications must be discontinued only if it is deemed clinically appropriate to do so and in consultation with the treating physician.

Subjects will be evaluated for the diagnosis of Rett syndrome. In addition, there must be verified documentation of a *MECP2* mutation. Genotyping may be done as part of the study if documentation is not adequate.

Caregivers will begin to keep a semi-structured caregiver diary during the screening period.

3.1.2 Double-Blind Treatment Period (12 Weeks)

The Baseline visit (Visit 2) may occur after screening procedures are completed and have not ruled the subject out of eligibility for the study. At Visit 2, and upon confirmation of eligibility, subjects will be randomized in a 1:1 ratio to trofinetide oral solution or matching placebo. All efficacy assessments (except the CGI-I) will be completed at the Baseline visit prior to administration of the first dose of study drug (Table S–2).

Dose will be based on the subject's weight at Baseline as outlined in Table 5-1. The dose may be decreased during the study as discussed in Section 5.1.3. Doses may be administered by gastrostomy (G) tube (doses administered via gastrojejunal [GJ] tubes must be administered through the G-port).

The first dose of study drug will be administered after all Baseline assessments are completed, or, if the Investigator judges that it is too late in the day, on the following day. The day the first dose is taken will be considered Day 1 of dosing. A triplicate ECG must be performed 2-3 hours after first dose and a PK sample will be taken upon completion of the ECG.

Study drug must be discontinued at any time during the study in the event that a post-randomization QTcF duration of ≥500 ms or an increase of ≥60 ms compared to the average QTcF interval at Baseline (before dosing) is observed. For visits at which more than one ECG is completed, the average QTcF interval of all legible ECGs will be used to determine the QTcF interval for that visit.

Dosing is twice a day, once in the morning and once in the evening.

Investigational product will be shipped directly to the subject. Confirmation of any delivery to the subject will be made by a visiting nurse. Study drug shipment, return, and accountability will be performed in accordance with the drug distribution plan. In addition, study drug will be dispensed at the site during the Baseline visit when the visit is conducted in the clinic.

Study visits may be done off-site rather than in the clinic with the prior approval of the Sponsor or Medical Monitor. Screening, Baseline, and EOT visits should be done in the clinic whenever possible.

For the 2 days before scheduled visits and the morning of the scheduled visit, the dates and times of study drug dosing, concomitant medication dosing, and meals will be recorded in the caregiver diary. Daily study drug dosing, including any dose modifications, missed or partial doses, will also be recorded in the caregiver diary.

3.1.3 Safety Follow-Up Period (30 Days)

A 30-day safety follow-up telephone or telemedicine contact is to be completed for subjects who complete the treatment period of the study and decide not to continue into the open-label study or are not eligible for the open-label study, as well as those who discontinue prematurely from the study. The telephone contact includes assessment of concomitant medications and treatments and assessment of AEs.

4 SUBJECT ELIGIBILITY AND WITHDRAWAL CRITERIA

To be eligible for this study, subjects must meet all of the inclusion criteria and none of the exclusion criteria.

4.1 Inclusion Criteria

A subject must meet all of the following inclusion criteria to be eligible for participation in the study:

1. Informed consent prior to the conduct of any study procedures is required as follows:
    a. For subjects who are minors: written informed consent will be obtained from the legally acceptable representative (LAR). The subject should provide written or oral assent if deemed able by the Investigator. The process of obtaining informed consent will be conducted in accordance with institutional review board (IRB) or ethics committee (EC) policy and applicable local law.
    b. For subjects who are not minors: written informed consent will be obtained from the LAR or the subject if deemed able by the Investigator. If the subject is deemed not able to provide consent, the subject should provide written or oral assent if deemed able by the Investigator. The process of obtaining informed consent will be conducted in accordance with IRB or EC policy and applicable local law.
    c. The subject's caregiver must also provide written informed consent regarding their participation in the study prior to participating in any study procedures.
2. Female subjects 5 to 20 years of age, inclusive, at Screening
3. Body weight ≥12 kg at Screening
4. Can swallow the study medication provided as a liquid solution or can take it by gastrostomy tube
5. The subject's caregiver is English-speaking and has sufficient language skills to complete the caregiver assessments

Diagnosis

6. Has classic/typical Rett syndrome (RTT) (Appendix A)
7. Has a documented disease-causing mutation in the *MECP2* gene
8. Is post-regression at Screening, defined as:
    a. No loss or degradation of ambulation (including gait, coordination, independence of walking/standing) within 6 months of Screening
    b. No loss or degradation of hand function within 6 months of Screening
    c. No loss or degradation of speech (including babbling, words or previously developed communicative vocalizations) within 6 months of Screening
    d. No loss or degradation of nonverbal communicative or social skills (including eye gaze, using body to indicate communicative intent, social attentiveness) within 6 months of Screening
9. Has a severity rating of 10-36, inclusive, on the Rett Syndrome Clinical Severity Scale at Screening
10. Has a CGI-S score of ≥4 at Screening and Baseline

Concomitant Treatment

11. If the subject is taking or was taking an anticonvulsant or any other psychoactive medication (including cannabinoids):
    a. the treatment regimen has been stable for at least 4 weeks before Baseline and there is no current plan to change the dose, OR
    b. if the medication was discontinued, the discontinuation has occurred no fewer than 2 weeks or 5 half-lives (whichever is greater) before Baseline
12. If the subject is taking or was taking any other medication daily for chronic illness (not including antibiotics, pain relievers, and laxatives):
    a. the treatment regimen of the medication has been stable for at least 4 weeks before Baseline and there is no current plan to change the dose, OR
    b. if the medication was discontinued, the discontinuation has occurred no fewer than 2 weeks or 5 half-lives (whichever is greater) before Baseline
13. If the subject is receiving or was receiving a non-pharmacologic somatic treatment (e.g., a ketogenic diet or vagal nerve stimulation):
    a. the treatment regimen has been stable for at least 4 weeks before Baseline and there is no current plan to change the treatment, OR
    b. if the treatment was discontinued, the discontinuation has occurred no fewer than 2 weeks before Baseline

14. If the subject is receiving or was receiving non-pharmacologic treatments such as an educational, behavioral, physical, occupational, or speech therapy:
    a. the treatment regimen has been stable for at least 4 weeks before Baseline and there is no current plan to change the treatment (Note: changes to a treatment regimen that are due to school schedules or are otherwise seasonally related are not exclusionary), OR
    b. if the treatment was discontinued, the discontinuation has occurred no fewer than 2 weeks before Baseline

Seizures

15. Has a stable pattern of seizures, or has had no seizures, within 8 weeks of Screening

Childbearing Potential

16. Subjects of childbearing potential must abstain from sexual activity for the duration of the study and for at least 30 days thereafter. If a subject is sexually active or becomes sexually active during the study, she must use 2 clinically acceptable methods of contraception (e.g., oral, intrauterine device [IUD], diaphragm plus spermicide, injectable, transdermal or implantable contraception) for the duration of the study and for at least 30 days thereafter. Subject must not be pregnant or breastfeeding.

Place of Residence

17. Subject and caregiver(s) must reside at a location to which study drug can be delivered and have been at their present residence for at least 3 months prior to Screening

4.2 Exclusion Criteria

A subject must meet none of the following exclusion criteria to be eligible for the study:

Concomitant Treatment

1. Has been treated with growth hormone within 12 weeks of Baseline
2. Has been treated with IGF-1 within 12 weeks of Baseline
3. Has been treated with insulin within 12 weeks of Baseline

Medical Conditions Other Than Rett Syndrome

4. Has current clinically significant cardiovascular, endocrine (such as hypo- or hyperthyroidism, Type 1 diabetes mellitus, or uncontrolled Type 2 diabetes mellitus), renal, hepatic, respiratory or gastrointestinal disease (such as celiac disease or inflammatory bowel disease) or has major surgery planned during the study
5. Has a history of, or current, cerebrovascular disease or brain trauma
6. Has significant, uncorrected visual or uncorrected hearing impairment

7. Has a history of, or current, malignancy

Laboratory Studies, Vital Signs, and Electrocardiogram

8. Has a clinically significant abnormal laboratory value at Screening. Laboratory testing may be repeated during the Screening period with agreement of the Medical Monitor.
9. Has serum potassium below the normal range for the subject (according to the central laboratory) at Screening. Serum potassium may be repeated during the Screening period with the agreement of the Medical Monitor.
10. Has a hemoglobin A1C (HbA1c) >7% at Screening
11. Has a thyroid stimulating hormone (TSH) value outside the normal range for the subject (according to the central laboratory) at Screening
12. Has clinically significant abnormality in vital signs at Screening or Baseline
13. Has any of the following:
    a. QTcF interval of >450 ms at Screening or Baseline (before dosing)
    b. History of a risk factor for torsades de pointes (e.g., heart failure or family history of long QT syndrome)
    c. History of clinically significant QT prolongation that is deemed to put the subject at increased risk of clinically significant QT prolongation
14. Has any other clinically significant finding on ECG at Screening or Baseline (before dosing)
15. Has a positive pregnancy test at Screening

Other Criteria

16. Has a significant sensitivity or allergic reaction to trofinetide or its excipients
17. Has participated in another interventional clinical study within 30 days prior to Screening
18. Is judged by the Investigator or the Medical Monitor to be inappropriate for the study for any reason

4.3 Subject Withdrawal of Consent

In accordance with the Declaration of Helsinki and other applicable regulations, a subject and legally acceptable representatives consenting on behalf of subjects have the right to withdraw from the study at any time, and for any reason, without prejudice to his or her future medical care.

Should a subject (or LAR) request or decide to withdraw consent, every reasonable effort will be made to complete and report observations as thoroughly as possible up to the date of withdrawal, including the evaluations specified at the ET or safety follow-up (whichever is applicable), as outlined in Table S–2.

4.4 Subject or Study Discontinuation

Subjects may be discontinued from the study for a number of reasons, including, but not limited to, those listed below:

- Adverse event
- Death
- Increase in post-Baseline QTcF interval (defined below and in Section 6.3.4)
- Lack of efficacy
- Lost to follow-up (Section 4.5)
- Non-compliance with study drug
- Physician decision
- Pregnancy
- Protocol deviation
- Study terminated by sponsor
- Use of prohibited medication
- Other

The Sponsor reserves the right to discontinue the study at any time for any reason. Such reasons may be any of, but not limited to, the following:

- Occurrence of AEs unknown to date in respect of their nature, severity, and duration or the unexpected incidence of known AEs
- Medical, ethical or business reasons affecting the continued performance of the study

Regulatory authorities also have the right to terminate the conduct of the study in their region for any reason.

4.4.1 Post-Baseline QTcF Interval Stopping Criteria

Study drug must be discontinued in the event that a post-randomization QTcF duration of ≥500 ms or an increase of ≥60 ms compared to the average QTcF interval at Baseline (before

dosing) is observed. For visits at which more than one ECG is completed, the average QTcF interval of all legible ECGs will be used to determine the QTcF interval for that visit.

4.4.2 Handling of Subject Discontinuation During the Treatment Period

Unless the subject has withdrawn consent (or the LAR has withdrawn consent on behalf of the subject) to be contacted for this study, every reasonable effort should be made to complete Visit 5/early termination (ET) and the safety follow-up (as outlined in Table S–2) if a subject discontinues prematurely for any reason. All information will be reported on the applicable pages of the electronic case report form (eCRF).

If a subject is discontinued from the study because of an AE, every reasonable attempt should be made to follow the subject until the AE resolves or until the Investigator deems the AE to be chronic or stable. For subjects who continue to be followed for safety, SAEs should continue to be reported as described in Section 7.3.2. All SAEs will continue to be followed until such events have resolved or the Investigator deems them to be chronic or stable.

Pharmacokinetic samples will also be collected, if possible, at any ET visit or the visit immediately following any SAE or following any AE leading to discontinuation, even if it is an unscheduled visit.

4.5 Subject Lost to Follow-Up

A subject will be considered lost to follow-up if they fail to attend a scheduled visit (excluding the safety follow-up telephone call) and the study site is unable to contact the subject or caregiver.

Every reasonable effort should be made to contact the caregiver and will include a minimum of 3 documented telephone calls and, if necessary, a certified letter to the caregiver's last known mailing address or local equivalent methods. All contact attempts are to be documented in the source documents.

4.6 Prior and Concomitant Therapy

All medications used up to 12 weeks prior to Baseline through completion of the safety follow-up visit or ET are to be recorded.

In order to ensure that appropriate concomitant therapy is administered, it is essential that caregivers be instructed not to administer any medication to the subject without prior consultation with the Investigator (unless the subject is receiving treatment for a medical emergency).

The Investigator may prescribe appropriate medication to treat AEs. The Sponsor and Investigator or designee will confer to determine whether it is appropriate to continue such a subject in the trial if a prohibited medication is prescribed.

Every effort should be made to maintain stable regimens of concomitant medications and allowed non-medicine based therapies throughout the course of the study, with the understanding that there will be some changes to a treatment regimen that are due to school schedules or are otherwise seasonally related. Special cases are medications taken for the treatment of constipation or that have diarrhea as an acute side effect. These may be adjusted as needed if diarrhea occurs.

4.6.1 Permitted and Prohibited Medications

Prohibited medications are IGF-1, growth hormone, and insulin. Prohibitions for concomitant medications will be followed between Visit 2 and Visit 5. Medications that can prolong QT interval are not prohibited but must be used with caution. Any use of medications that could interfere with study conduct must be discussed with the Medical Monitor.

Use of medications that could interfere with study conduct or any questions regarding concomitant medications must be reviewed and/or discussed with the Medical Monitor.

Psychoactive concomitant medications should remain at a stable dose throughout the study if possible. Any non-pharmacologic somatic treatment regimen (e.g., a ketogenic diet or vagal nerve stimulation) that has CNS effects should remain stable throughout the study if possible. Treatment of constipation may be changed as needed.

Subjects who require current treatment with a prohibited medication will be withdrawn from the study.

Subjects who have previously taken a prohibited medication during the study will be withdrawn from the study unless:

- the prohibited medication has been discontinued AND
- withdrawal from the study presents an unacceptable medical risk to the subject

The justification to allow the subject to continue in the trial will be made by the Sponsor/Medical Monitor, with medical input from the Investigator, and will be documented. If a subject is allowed to remain in the trial, this will be reported as a major protocol deviation and not a waiver.

5 INVESTIGATIONAL PRODUCT

5.1 Investigational Product Description

The investigational product will be trofinetide oral solution or matching placebo for trofinetide oral solution. Dose will be based on weight as outlined in Table 5-1. Trofinetide will be provided in a ready-to-use aqueous solution for oral administration. Doses will be administered orally or by G-tube (doses administered by GJ tubes must be administered through the G-port).

5.1.1 Formulation, Appearance, and Packaging

The Sponsor will supply trofinetide oral solution and matching placebo for trofinetide oral solution as an aqueous, ready-to-use, strawberry-flavored liquid in 500 mL (16 oz) round, high-density polyethylene (HDPE) plastic bottles with a child-resistant closure.

Trofinetide oral solution is a clear, pink to red-colored liquid containing 1 gram of trofinetide in each 5 mL. The trofinetide oral solution also contains purified water, maltitol, strawberry flavor, sucralose, methylparaben sodium, propylparaben sodium, and FD&C Red #40 as inactive ingredients.

The placebo aqueous solution does not contain trofinetide active pharmaceutical ingredient (API), but contains purified water, acetic acid, caramel color, citric acid, lemon flavor, maltitol, methylparaben sodium, natural quinine flavor, propylparaben sodium, FD&C Red #40, strawberry flavor, sucralose, xanthan gum, and D&C Yellow #10.

Trofinetide and matched placebo are manufactured under current Good Manufacturing Practices.

During the treatment period, study drug will be distributed in a quantity sufficient to ensure the subject has an adequate supply of study drug between study visits.

5.1.2 Product Storage and Stability

Investigational product will be shipped refrigerated at a temperature between 2°C and 8°C (36°F and 46°F) and will be stored at this temperature. Do not freeze.

5.1.3 Dosing and Administration

5.1.3.1 Dosing

Dosing of study drug is based on weight. In the Phase 2 study, Study RETT-002, the dose for each subject was assigned based on weight using a single value in mg/kg for each subject in a particular dose arm. As a result, each subject received a unique dose of study drug to be administered twice a day. Instead of assigning a unique dose of study drug for the dose based on each individual's weight, in the present study the subject is assigned one of four standard

doses, also based on the weight of the individual. The assigned dose is based on the range of weight in which the subject falls at Baseline (see Table 5-1).

This approach is intended to balance: 1) simplification of assigning the dose and of packaging and dispensation of the study drug with 2) giving each subject a dose that is expected to be effective for subjects at all weights (see Section 1.4).

The dose assigned to each subject is based on the subject's weight at Baseline. The dose will not be increased or decreased if the subject's weight at a postbaseline visit puts them in a new weight category. The dose may be decreased during the study for poor tolerability as discussed in Section 5.1.3.2.

Study drug is administered twice a day, once in the morning and once in the afternoon or evening. There should be at least 8 hours between doses.

Table 5-1 Dosing Schedule Based on Weight at Baseline

Weight	Dose	Total Daily Dose
12-20 kg	30 mL (6 g) BID	60 mL (12 g)
>20-35 kg	40 mL (8 g) BID	80 mL (16 g)
>35-50 kg	50 mL (10 g) BID	100 mL (20 g)
>50 kg	60 mL (12 g) BID	120 mL (24 g)

Abbreviations: BID=twice daily

The first dose of study drug will be administered after all Baseline assessments are completed. If the Investigator judges that it is too late in the day to administer study drug, it may be administered on the following day. The day the first dose is taken will be considered Day 1 of dosing. An ECG must be performed 2-3 hours after the first dose and a PK sample will be taken upon completion of the ECG. Off-site dosing at Baseline may only be done if it is possible to record an ECG 2-3 hours after the first dose and draw blood for a PK sample upon completion of the ECG. The investigator must review the ECG readings with special attention to the QTcF interval before the subject can receive another dose of study drug. Off-site dosing at Baseline must first be approved by the Sponsor or Medical Monitor.

Investigational product will be shipped directly to the subject. Confirmation of any delivery to the subject will be made by a visiting nurse. Study drug shipment, return, and accountability will be performed in accordance with the drug distribution plan. In addition, study drug will be dispensed at the site during the Baseline visit when the visit is conducted in the clinic.

5.1.3.2 Administration of Study Drug

Study drug is supplied in 500 mL (16 oz) bottles with a child-resistant closure. A press-in bottle adapter and syringe for accurately measuring the dose are supplied separately.

The study drug must not be mixed with any food or liquid, including water.

Doses may be taken orally or via gastrostomy tube.

- When study drug is given orally, it may be administered directly from a syringe or transferred into another container to facilitate administration.
- For gastrojejunal tubes, medication should be given via the gastric port. The tube must be flushed with water after study drug administration to clear the tube of study drug. No more than 250 mL of water should be used. Doses should be taken over a 10-minute period.

If the subject cannot tolerate administration of the full assigned dose (for example, if the subject experiences diarrhea) any time before the Week 6 visit, the Investigator may instruct the caregiver to reduce study drug to a dose as low as half the assigned dose. In addition, up to four doses (in total, consecutive or non-consecutive) may be withheld within the first 6 weeks. The Investigator must attempt to increase the dose as soon as it is possible based on the clinical situation. The aim is to return to the originally assigned dose.

If the originally assigned dose cannot be reached, or the subject is again unable to tolerate that dose, the Investigator will continue treatment on the highest dose the subject can tolerate. The final dose, i.e., the highest tolerated dose, must be given BID, once in the morning and once in the evening, unless the subject is eligible and is continuing in the open-label extension study. Subjects who continue into the extension study will take their final dose for the current study the morning of Visit 5.

The dose cannot be changed after the Week 6 visit.

5.1.4 Method of Assigning Subjects to Treatment Groups

At Visit 2, eligible subjects who meet inclusion and do not meet exclusion criteria will be randomized in a 1:1 ratio to receive either trofinetide or placebo.

5.1.5 Blinding

Treatment assignments will be blinded to all study subjects, caregivers, Investigators, raters, site personnel, and Sponsor personnel. In the event of a potential suspected unexpected serious adverse reaction (SUSAR), in accordance with current health authority guidance, treatment assignments for the affected subject may be unblinded to a controlled group of the Sponsor’s Safety and/or Regulatory personnel for reporting purposes.

Details regarding medical emergency unblinding procedures are provided in Section 9.9.

5.1.6 Study Drug Compliance

If a subject misses one dose of study drug, she must not take an extra dose the next day.

5.1.7 Overdose

An overdose is a deliberate or inadvertent administration of a treatment at a dose higher than the maximum recommended dose per protocol. It must be reported, irrespective of outcome, even if toxic effects were not observed (Section 7.3.4). All events of overdose are to be captured as protocol deviations.

5.2 Investigational Product Accountability Procedures

The Investigator or designee will keep current and accurate records of the study drug product dispensed, used, and returned for each subject to assure the health authority and the Sponsor that the study drug is being handled appropriately. Caregivers must be instructed to return all packaging to the Investigator or designee at regularly scheduled clinic or home nursing visits as appropriate.

At appropriate intervals during the study, study drug reconciliation will be performed by the Sponsor (or designee) who may return appropriate unused study drug and used and unused packaging to the Sponsor’s designee for destruction.

At the conclusion of the study, final study drug reconciliation will be conducted at the site. Final study drug accountability documentation will be maintained at both the site and by the Sponsor or designee. Any remaining unused study drug and all used and unused packaging will be sent back to the Sponsor’s designee for destruction or destroyed at the site, as allowed by country-specific regulations. Documentation of study drug destruction will be recorded and maintained by both the Sponsor and the Sponsor’s designee.

The Investigator or designee is responsible for taking an inventory of each shipment of study drug received and comparing it with the accompanying material shipping form. The Investigator or designee will verify the accuracy of the information on the form, sign and date it, and provide a copy of it to the Sponsor or designee. Any study drug supplied is for use in this study only and must not be used for any other purpose.

6 STUDY PROCEDURES

Study-specific procedures are detailed below. All assessments will be completed according to the schedule described in Table S–2. Every effort should be made to complete the required procedures and evaluations at the designated visits and times.

6.1 Screening Assessments

6.1.1 Confirm Diagnosis of Rett Syndrome and *MECP2* Mutation

The site will confirm that the subject meets criteria for typical/classic Rett syndrome (Appendix A) and that there is documentation of disease-causing mutation of the *MECP2* gene. The genotyping must have been performed at a laboratory certified by the College of American Pathologists (CAP), or under the Clinical Laboratory Improvement Act/Amendment (CLIA), or by an equivalent organization. If documentation of the mutation is not adequate, genomic testing for a mutation in the *MECP2* gene may be conducted as part of Screening.

The documented mutation must be associated with Rett syndrome, such as according to the *MECP2* mutation database RettBASE (http://mecp2.chw.edu.au/cgi-bin/mecp2/search/search.cgi?form=combined) (Krishnaraj et al. 2017). If there is any question regarding the association of the mutation with Rett syndrome, the Medical Monitor must be consulted. The disease-causing mutation will be documented in the source documentation and eCRF.

6.1.2 Medical History, Including Rett Syndrome History, and Demographics

A complete medical history, including history of symptoms associated with Rett syndrome, will be performed at Screening to document all current medical conditions, and previous major medical events and conditions. For subjects who were already receiving care for Rett syndrome at the study site, summary documents from the medical record (such as clinician's summaries) should be available as source documentation of major medical conditions or events (e.g., surgeries). For subjects who were not a part of the clinical site's practice, the study team should make every effort to obtain summary medical records from their other providers in preparation of the screening visit.

6.1.3 The Rett Syndrome Clinical Severity Scale (RTT-CSS)

The RTT-CSS has been evaluated in over 1200 RTT children, adolescents and adults enrolled in the NIH-sponsored Natural History of Rare Diseases Project. This scale has been used as a measure of severity as reported in studies of genotype/phenotype correlations and epilepsy in RTT (Glaze et al. 2010) and was evaluated as an outcome measure in the Neu-2566-RETT-001 study of trofinetide in adolescent and adults with RTT. The scale was derived from that reported in Amir et al. (2000) and Monrós et al. (2001).

The RTT-CSS is a clinician-completed rating scale that measures the severity of core symptoms of RTT. The CSS consists of 13 items, 3 of which measure historical or static characteristics (age of onset of regression, age of onset of stereotypes, head growth) and

10 of which measure current function (somatic growth, independent sitting, ambulation, hand use, scoliosis, language, nonverbal communication, respiratory dysfunction, autonomic symptoms, and epilepsy/seizures) at the time of assessment, i.e., during the study visit.

All items are scored during a clinical interview and examination by the Investigator or qualified designee using either a 4- or 5-point Likert scale. Individual subscale scores and a total score are calculated.

The RTT-CSS will be administered at Screening only.

6.2 Efficacy Assessments

All assessments will be administered in a standardized manner. Clinician completed measures will be completed by trained practitioners. Caregiver completed assessments will be reviewed by study personnel and caregivers will receive standardized training and guidance on how to complete the measures. To the extent possible, all efforts should be made to maintain the same caregiver (i.e., caregiver rater) and clinician rater (as applicable) across visits for a single subject.

All efficacy assessments designated to be completed at Baseline are to be performed prior to administration of the first dose of study drug.

6.2.1 Rett Syndrome Behaviour Questionnaire (RSBQ)

The Rett Syndrome Behaviour Questionnaire (RSBQ) is a 45 item caregiver-completed rating scale assessing a wide range of neurobehavioral symptoms known to be impaired in RTT (Mount et al. 2002). The RSBQ is a well-validated instrument that has been used in the Phase 2 study, Neu-2566-RETT-002, as well as in other observational and interventional studies in RTT (Glaze et al. 2019; Khwaja et al. 2014; O'Leary et al. 2018). The RSBQ has been correlated with functioning and quality of life and has been characterized and validated across a range of ages and genetic variations in RTT (Cianfaglione et al. 2015, 2016; Robertson et al. 2006; Barnes et al. 2015). The scale includes 45 items, including 8 subscales, whose ratings reflect the severity and frequency of symptoms. The caregiver rates items as "0" (Not True), "1" (somewhat or sometimes true) or "2" (very true). The eight subscales include:

1. General Mood
2. Breathing problems
3. Hand Behavior
4. Face movements
5. Body rocking/expressionless face
6. Night-time behaviors

7. Fear/anxiety
8. Walking/standing

The RSBQ will be administered at Screening, Baseline (Visit 2), and Visits 3, 4, and 5.
As much as possible, caregiver raters will remain the same throughout the study. At the start of the study all caregiver raters will be required to complete a standardized training on how to complete the scale.

6.2.2 Clinical Global Impression–Improvement (CGI-I) and Clinical Global Impression–Severity (CGI-S)

The CGI-I scale will be administered at Visits 3, 4, and 5. Completion of this scale requires the clinician to rate how much the subject's illness has improved or worsened relative to a baseline state. A 7-point scale is used from 1=very much improved, 2=much improved, 3=minimally improved, 4=no change, 5=minimally worse, 6=much worse, 7=very much worse.

The CGI-S assessment will be made at Screening, Baseline, and Visits 3, 4, and 5. The CGI-S is a 7-point scale that requires the clinician to rate the severity of the subject's illness at the time of assessment, relative to the clinician's experience with subjects who have the same diagnosis. Considering total clinical experience, a subject is assessed on severity of illness at the time of rating: 1, normal, not at all ill; 2, borderline ill; 3, mildly ill; 4, moderately ill; 5, markedly ill; 6, severely ill; or 7, extremely ill.

In this study, the illness being assessed is Rett syndrome as a whole.

Following best practice, the CGI-S and CGI-I ratings for the study will be assessed using RTT-specific anchors across major symptom areas in the same manner as in the Phase 2 studies (Neul et al. 2015; Busner and Targum 2007; Glaze et al. 2017; Glaze et al. 2019).

6.2.3 Communication and Symbolic Behavior Scales Developmental Profile Infant-Toddler (CSBS-DP-IT) Checklist

The Communication and Symbolic Behavior Scales-Developmental Profile™ (CSBS-DP) is standardized screening scale for assessing communication and pre-linguistic skills in young children 12-24 months (Wetherby et al. 2002) and can be used with older children with developmental delay (Anagnostou et al. 2015; Urbanowicz et al. 2016). The CSBS-DP includes a suite of three separate measures: The Infant-Toddler Checklist, a follow-up Caregiver Questionnaire and a Behavior Sample. In this study only the Infant-Toddler (CSBS-DP-IT) Checklist will be used.

Given the limited communication abilities of individuals with Rett Syndrome, the CSBS-DP-IT Checklist was assessed and a subset of items were found to be appropriate for assessing communication skills of individuals with Rett syndrome 8 to 19 years of age (Urbanowicz et al. 2016). The CSBS-DP-IT was assessed in a Phase 2 trial of mecasermin (recombinant human IGF-1) a compound related to trofinetide, in children with Rett syndrome 2 to 10 years of age (O'Leary et al. 2018). In that study, the first 16 items were completed, which allowed for calculation of the Social composite score. The CSBS-DP-IT demonstrated evidence of benefit in subjects in the active treatment group compared to those in the placebo treatment group (O'Leary et al. 2018). The CSBS-DP Social composite score has also shown evidence of sensitivity to change in behavioral intervention studies in other developmental disorders (e.g., Wetherby et al. 2014; Anagnostou et al. 2015).

The CSBS-DP-IT Checklist is a 24-item rating scale completed by the caregiver. Each item is scored using a three-level rating of frequency: "not yet", "sometimes" and "often". Three composite scores assessing 7 skill areas can be calculated: 1) Social Composite (including Emotion and Eye Gaze, Communication Rate and Function, and Gestures); 2) Speech Composite (including Sounds and Words); 3) Symbolic Composite (including Understanding and Object Use).

All 24 items on the Infant-Toddler Checklist are to be completed by the caregiver. The question after item 24 ("Do you have any concerns about your child's development?") is not to be completed. At each administration, the study staff will review the instructions and scoring rubric with the caregiver. The Social Composite raw score, comprised of items 1 to 13, is used for the key secondary endpoint.

The CSBS-DP-IT Checklist will be administered at Baseline, Visit 3, Visit 4, and Visit 5.

6.2.4 Impact of Childhood Neurologic Disability (ICND) Scale

The Impact of Childhood Neurologic Disability (ICND) scale was developed to evaluate the impact that a child's condition has on the child's and the family's everyday life at the present time and during the previous 3 months (Camfield et al. 2003). The parent or other caregiver evaluates the effect of four conditions or health problems on 11 aspects of the child's or the family's life as "A lot", "Some", "A little", "Not at all", or "Does not apply". The four conditions or health problems are 1) inattentiveness, impulsivity, or mood, 2) ability to think and remember, 3) neurologic or physical limitations, and 4) epilepsy.

The caregiver then rates overall quality of life of the subject by responding to the following: "Please rate your child's overall 'Quality of Life' on the scale below. Choose the number which you feel is best and circle it". The choices range from 1 ("Poor") to 6 ("Excellent").

The assessment will be administered at Baseline and Visit 5.

6.2.5 Rett Syndrome Clinician Rating of Hand Function (RTT-HF)

The Rett Syndrome Clinician Rating of Hand Function is a clinician completed clinical assessment of the subject's ability to use her hands for functional purposes (such as reaching for and grasping objects, self-feeding or drawing). The assessment is made on an 8-point Likert scale (0-7) with 0 denoting normal functioning and 7 the most severe impairment. This rating is a further development of the RTT-DSC Hand Use Rating used in Study Neu-2566-RETT-002.

The assessment will be administered at Baseline, Visit 3, Visit 4, and Visit 5.

6.2.6 Rett Syndrome Clinician Rating of Ambulation and Gross Motor Skills (RTT-AMB)

The Rett Syndrome Clinician Rating of Ambulation and Gross Motor Skills is a clinician completed clinical assessment of the subject's ability to sit, stand, and ambulate (e.g., walking, running, climbing stairs). The assessment is made on an 8-point Likert scale (0-7) with 0 denoting normal functioning and 7 the most severe impairment. This rating is a further development of the RTT-DSC Ambulation Rating used in Study Neu-2566-RETT-002.

The assessment will be administered at Baseline, Visit 3, Visit 4, and Visit 5.

6.2.7 Rett Syndrome Clinician Rating of Ability to Communicate Choices (RTT-COMC)

The Rett Syndrome Clinician Rating of the Ability to Communicate Choices is a clinician completed clinical assessment of the subject's ability to communicate her choices or preferences, which can include the use of nonverbal means such as eye contact or gestures. The assessment is made on an 8-point Likert scale (0-7) with 0 denoting normal functioning and 7 the most severe impairment. This rating is a further development of the RTT-DSC Language/Communication Rating used in Study Neu-2566-RETT-002.

The assessment will be administered at Baseline, Visit 3, Visit 4, and Visit 5 when the visit takes place in the clinic. When a study visit takes place off-site, the RTT-COMC should be completed if possible, but it is not required.

6.2.8 Rett Syndrome Clinician Rating of Verbal Communication (RTT-VCOM)

The Rett Syndrome Clinician Rating of Verbal Communication is a clinician completed clinical assessment of the subject's ability to communicate verbally (e.g., words and phrases). The assessment is made on an 8-point Likert scale (0-7) with 0 denoting normal functioning and 7 the most severe impairment. This rating is a further development of the RTT-DSC Language/Communication Rating used in Study Neu-2566-RETT-002.

The assessment will be administered at Baseline, Visit 3, Visit 4, and Visit 5.

6.2.9 Rett Syndrome Caregiver Burden Inventory (RTT-CBI)

The RTT-CBI is a syndrome-specific, caregiver-completed questionnaire that is based on the Caregiver Burden Inventory designed for Alzheimer's disease (Lane et al. 2017; Novak and Guest 1989). The scale is intended to directly address caregiver burden and indirectly assess the significance of treatment effects on function in the context of activities of daily living. Caregivers rate how often a given statement describes their feeling or experience. Frequency ratings are on a 5-point Likert scale including: 0-never; 1-rarely; 2-sometimes; 3-frequently and 4-nearly always. As in the original Caregiver Burden Inventory, the RTT-CBI has 24 negatively worded items (items 1 through 24) yielding a total score up to 96. The RTT-CBI also includes 2 positively worded items (items 25 and 26) that comprise the Optimism Index (Lane et al. 2017). In this study, as in the 2 previous studies of trofinetide in Rett syndrome, the total score is defined as the total Burden score (items 1-24).

The RTT-CBI will be completed at Baseline and Visit 5.

6.3 Safety Assessments

6.3.1 Physical Examination

A general physical examination will be conducted at Screening, Baseline, Visit 3, Visit 4, and Visit 5. When a study visit takes place off-site, the physical examination will not be required. The physical exam procedures will include the following organ systems:

- Neurological
- Head, ears, eyes, nose, and throat
- Skin
- Cardiovascular
- Respiratory
- Abdomen
- Genitourinary (optional)
- Musculoskeletal

6.3.2 Vital Signs

Vital signs will include body temperature, resting respiration rate, sitting systolic and diastolic blood pressure, and pulse rate. The sitting blood pressure will be measured after the subject has been sitting for ≥3 minutes.

Vital signs to be measured at Screening, Baseline, Visit 3, Visit 4, and Visit 5.

6.3.3 Height, Weight, and Body Mass Index

Height will be measured at Screening and at Visit 5.

Weight will be measured at Screening, Baseline, Visit 3, Visit 4, and Visit 5 when the visit takes place in the clinic. When a study visit takes place off-site, weight should be measured whenever possible.

Body mass index will be calculated using the following formula:
Weight (kg) / [height (m)]^2.

6.3.4 Electrocardiograms

All 12-lead ECGs will be complete, standardized recordings, whenever possible. ECGs will be completed in triplicate at Visit 1 (Screening), at Visit 2 (Baseline) both before dosing and 2-3 hours after dosing, and at Visit 5 (Week 12/EOT/ET). A single ECG will be completed at Visit 3 (Week 2) and Visit 4 (Week 6). For visits at which more than one ECG is completed, the average QTcF interval of all legible ECGs will be used to determine the QTcF interval for that visit.

The subject should rest for ≥5 minutes in a supine position before the ECG is obtained. If it is impractical to rest in supine position due to the subject's medical condition, the subject may remain in a partial supine or other position (e.g., upright in wheelchair) for ≥5 minutes before the ECG is obtained and when the ECG is obtained. ECG tracings (paper or electronic) will be reviewed and interpreted by a qualified clinician for prolongation of the QTcF interval and for other cardiac irregularities. ECG tracings and results (ventricular rate, PR, QRS, QT, QTcF and QTcB intervals) will be included in the subject's study records.

ECGs will also be read by a qualified central reader. The central reading will be the reading that is entered in the database. The results from the reports from the central reader will also be reviewed by the Investigator. At Screening and Baseline (before dosing) the average QTcF interval of all legible ECGs will be used to determine eligibility.

If the average QTcF value from the set of ECGs done at Screening is prolonged due to an identifiable cause, and it is medically appropriate to address that cause, a repeat set of triplicate ECGs may be performed during the Screening period and before the Baseline visit with the agreement of the Medical Monitor. In this case, the repeat ECGs will be used in determination of subject eligibility.

6.3.4.1 Post-Baseline QTcF Interval Stopping Criteria

In the event that a post-randomization QTcF duration of ≥500 ms or an increase of ≥60 ms compared to the average QTcF interval at Baseline (before dosing) is observed, study drug administration is to be discontinued. For visits at which more than one ECG is completed, the average QTcF interval of all legible ECGs will be used to determine the QTcF interval for that visit.

6.3.5 Laboratory Evaluations

Laboratory evaluations will be completed according to the schedule presented in Table S–2 and procedures detailed in the laboratory manual. Additional safety testing may be performed at the discretion of the Investigator or designee. Laboratory testing may be repeated during the Screening period with agreement of the Medical Monitor if the Investigator suspects that a laboratory abnormality is a temporary or reversible finding.

Clinical laboratory sample collection is not required to be completed under fasting conditions. The laboratory evaluations will include the following:

- Clinical chemistry serum tests
  - Sodium (Na), potassium (K), chloride (Cl), phosphorus (P), calcium (Ca), magnesium (Mg), carbon dioxide (CO_2), blood urea nitrogen (BUN), creatinine (CR), uric acid
    - Mg will only be performed at Visit 1 (Screening)
  - Alanine aminotransferase (ALT), aspartate aminotransferase (AST), gamma-glutamyl transpeptidase (GGT), alkaline phosphatase (ALP), total bilirubin (TBIL), lactate dehydrogenase (LDH)
  - HbA1c
    - HbA1c will only be performed at Visit 1 (Screening)
  - Glucose
  - Albumin (ALB), total protein
  - Thyroid stimulating hormone (TSH), free T3, and free T4
    - Thyroid function tests will be performed at Visit 1 (Screening), Visit 2 (Baseline), and Visit 5
- Pregnancy test
  - A serum pregnancy test will be performed at the designated visits (Table S–2) for subjects of childbearing potential

- Hematology tests
  - Complete blood count (CBC) including:
    - White blood cell (WBC) count
    - Complete differential (relative and absolute)
    - Hematocrit (Hct), hemoglobin, red blood cells (RBC), platelets
    - Reticulocyte count
- Urinalysis
  - Blood, RBCs, WBCs, protein, glucose, ketones, specific gravity, pH
  - Reasonable efforts will be made to collect a urine sample from all subjects. When collection of a urine sample proves impractical or impossible (e.g., because the subject is incontinent or unable to cooperate), failure to collect a urine sample will be recorded in the subject's eCRF, and will not be considered a protocol deviation.

6.4 Caregiver Diary

A semi-structured caregiver diary will be completed during screening and treatment in which caregivers will record seizures and seizure-like spells if they are present.

At the Screening visit, the clinician will review the subject's seizure profile (if applicable) and will help the family to characterize the subject's typical seizure types. These are recorded in the caregiver diary.

For the 2 days before scheduled visits as well as the morning of the scheduled visit, the dates and times of study drug dosing, concomitant medication dosing, and meals will be recorded in the caregiver diary. Daily study drug dosing, including any dose modifications, missed or partial doses, will also be recorded in the caregiver diary.

The caregiver diary will be completed and collected on an ongoing basis throughout the study from Screening to Visit 5. The clinician will verbally ask about AEs (see Section 7.3.1), review the events recorded in the diary with the caregiver and will make a clinical evaluation, including an evaluation of whether an adverse event will be reported.

6.5 Pharmacokinetic Assessments

Pharmacokinetic blood samples will be collected for measurement of whole blood concentrations of trofinetide and possible metabolites identified.

PK blood samples will be collected at 5 timepoints for trofinetide concentration measurements at the Baseline visit (both before dosing and approximately 2-3 hours after

dosing) and at Visit 3, Visit 4, and Visit 5 in accordance with the sampling schedule outlined below (Table 6–1).

The second PK sample to be taken at the Baseline visit (Visit 2), approximately 2-3 hours after dosing, will take place as soon as possible after the postdose ECG is performed.

PK samples at Visits 3, 4, and 5 should be collected at one of the following time intervals:

- 2-3 hours after dosing
- 4-6 hours after dosing
- 7-11 hours after dosing

Every effort should be made to collect PK samples at discrete time intervals during Visits 3, 4, and 5. However, if the interval is the same across these visits, then the collection time should vary within that interval. The following scenario is only for illustrative purposes, a number of other scenarios are possible.

- For example, if the time interval of 4-6 hours after dosing is used for Visits 3, 4, and 5, then every effort should be made to collect PK samples at 4, 5, and 6 hours after dosing, respectively.

Pharmacokinetic samples will also be collected, if possible, at any ET visit or the visit immediately following any SAE or following any AE leading to discontinuation, even if it is an unscheduled visit.

For all scheduled PK samples (and for unscheduled samples if possible), the dates and times of administration of the study drug, the dates, times and content of the meals, and the dates and times of the administration of concomitant medication over the 2 days prior to and on the morning of the PK sample draw, as well as the date and time of the sample draw, will be recorded. For samples collected from subjects who experience any SAE or experience an AE leading to discontinuation, the date and time of the last dose of study drug prior to the SAE or AE leading to discontinuation will also be recorded.

Table 6–1 PK Sampling Times

Visit	Timing of Sample<br>(relative to start of dosing on the visit day)	Week of Treatment
2 (Baseline)	Any time before dosing<br>and<br>2 – 3 hours after dosing (after ECG)	Week 0 (Baseline)
3, 4, and 5	2 – 3 hours after dosing<br>or<br>4 – 6 hours after dosing<br>or<br>7 – 11 hours after dosing	Weeks 2, 6, and 12/EOT

Abbreviations: ECG=electrocardiogram; EOT=end of treatment; PK=pharmacokinetic

6.5.1 Specimen Preparation, Handling, Storage, and Shipment

PK blood samples may be collected from a cannula port or via venipuncture. Pre-prepared PK sampling tubes will be provided to each site within the lab visit kits for collection and storage of PK samples. Blood samples will be processed for determination of trofinetide whole blood concentrations (and of concentrations of possible metabolites identified). At each time point, blood will be collected, processed as appropriate, and samples will be shipped to the central laboratory for storage and to the bioanalytical laboratory for analysis. A laboratory manual will be provided for sample processing, storage, and shipping procedures.

When possible, an additional PK sample will be collected from subjects who experience any SAE or experience an AE leading to discontinuation as soon as possible after the occurrence of that event.

6.6 Identification of Biomarkers of Response to Trofinetide in Rett Syndrome

Subjects for whom separate informed consent for the identification of biomarkers of response to trofinetide is provided (where local regulations permit) will have blood drawn and stored for future investigations. Blood samples will be taken at Baseline (before dosing) and at Visit 5, or upon early termination. Participation in the effort to identify biomarkers is an optional component of the study requiring a separate informed consent, which may be obtained at any time during the study. If consent is obtained after Baseline, only the sample at Visit 5, or upon early termination will be taken.

Blood samples will be used to investigate differences between responders and non-responders in both trofinetide-treated and placebo-treated subjects in RNA transcripts (transcriptomics), proteins (proteomics), and metabolites (metabolomics). Unbiased analyses and targeted analyses will test candidate molecular pathways based on the available knowledge of RTT

and trofinetide at the time of the investigation (Erhart et al. 2016; Shovlin and Tropea 2018; West et al 2014; Buchovecky et al. 2013). The analysis of biomarkers does not include any DNA, genomic, or genetic testing or analysis.

Stored samples and relevant clinical data will be made non-identifiable after the clinical study report has been issued. Any personal identifiers will be removed, and each study subject identifier will be replaced with a new number to limit the possibility of linking genetic data to a subject's identity.

7 ADVERSE EVENTS

7.1 Specification of Safety Parameters

7.1.1 Definition of Adverse Event

An AE is defined as "any untoward medical occurrence in a patient or clinical study participant, temporally associated with the use of study drug, whether or not considered related to study drug".

An AE can therefore be any unfavorable and unintended sign (e.g., an abnormal laboratory finding), symptom, or disease temporally associated with the use of a drug, without any judgment about causality or seriousness. An AE can arise from any use of the drug (e.g., off-label use, use in combination with another drug) and from any route of administration, formulation, or dose, including an overdose.

A suspected adverse reaction is any AE for which there is a reasonable possibility that the drug caused the AE.

AEs do not include the following:

- Stable or intermittent chronic conditions (such as myopia requiring eyeglasses) that are present prior to Baseline and do not worsen during the study
- Medical or surgical procedures (e.g., surgery, endoscopy, tooth extraction, transfusion). The condition that leads to the procedure is an AE if not present at Baseline.
- Overdose of concomitant medication without any signs or symptoms unless the subject is hospitalized for observation
- Hospitalization for elective surgery planned prior to study (situation where an untoward medical occurrence has not occurred)
- Pregnancy will not be considered an AE, but if it occurs, it will be reported on a pregnancy form

For subjects who enroll into the open-label extension study, AEs will be recorded from the time informed consent is obtained in the present study until the first dose of study drug in the open-label study.

For subjects who discontinue from the study or do not enroll into the open-label extension, AEs will be recorded from the time informed consent is obtained until 30 days after the last dose of study drug.

7.1.2 Definition of Serious Adverse Event

In addition to the severity rating, each AE will be classified by the Investigator as "serious" or "not serious." The seriousness of an event will be defined according to the applicable regulations and generally refers to the outcome of an event. An SAE is one that meets one or more of the following:

- Is fatal
- Is immediately life threatening
- Results in disability or permanent damage
- Requires hospitalization
- Prolongs existing hospitalization
- Is a congenital anomaly or birth defect (in an offspring)
- Is medically significant

Definition of Life Threatening

A life-threatening event places the subject at immediate risk of death from the event as it occurred. This does not include an AE, which, had it occurred in a more severe form, might have caused death.

Definition of Hospitalization

Hospitalization is defined by the Sponsor as a full admission to the hospital for diagnosis and treatment. This includes prolongation of an existing inpatient hospitalization.

Examples of visits to a hospital facility that do **not** meet the serious criteria for hospitalization include:

- Emergency room visits (that do not result in a full hospital admission)
- Outpatient surgery
- Preplanned or elective procedures

- Protocol procedures
- Social hospitalization, defined as admission to the hospital as a result of inadequate family support or care at the subject's primary residence

Definition of Disability or Permanent Damage

Disability is defined as a persistent or significant incapacity or substantial disruption of the ability to conduct normal life functions.

Definition of Medically Significant

Important medical events (medically significant events) that may not result in death, be life threatening, or require hospitalization may be considered to be an SAE when, based upon appropriate medical judgment, they may jeopardize the subject or may require medical or surgical intervention to prevent one of the outcomes listed in this definition. Examples of such events are intensive treatment in an emergency room or at home for allergic bronchospasm, blood dyscrasias, or convulsions that do not result in hospitalization or development of drug dependency or drug abuse.

An SAE may also include any other event that the Investigator or Medical Monitor judges to be serious or that suggests a significant hazard, contraindication, side effect, or precaution.

7.2 Classification of an Adverse Event

7.2.1 Severity of Event

The severity of each AE will be graded on a 3-point scale and reported in detail as indicated on the eCRF:

- **Mild**: awareness of sign or symptom but easily tolerated, causing minimal discomfort, and not interfering with normal everyday activities
- **Moderate**: sufficiently discomforting to interfere with normal everyday activities
- **Severe**: incapacitating and/or preventing normal everyday activities

7.2.2 Relationship to Study Drug

The causality of each AE should be assessed and classified by the Investigator as "related" or "not related." An event is considered related if there is a reasonable possibility that the event may have been caused by the product under investigation (i.e., there are facts, evidence, or arguments to suggest possible causation).

Consider the following when assessing causality:

- Temporal associations between the agent and the event
- Response to cessation (de-challenge) or re-challenge
- Compatibility with known class effect
- Known effects of concomitant medications
- Pre-existing risk factors
- A plausible mechanism
- Concurrent illnesses

7.2.2.1 Duration

The start and stop dates for AEs will be recorded using the following criteria:

- **Start:** Date of the first episode of the AE or date of significant sustained worsening in severity
- **Stop:** Date when AE either ceased permanently or changed in severity

For AEs of diarrhea, the start date and stop date of a decrease in severity will also be recorded.

7.2.2.2 Frequency

The frequency of the AE should be indicated according to the following definitions:

- **Single:** Experienced once, without recurrence
- **Recurrent:** More than one discrete episode with the same severity

7.2.2.3 Action Taken with Study Drug

- **Dose not changed:** No change in study drug
- **Drug interrupted:** Study drug temporarily stopped
- **Drug withdrawn:** Study drug discontinued permanently
- **Dose decreased**: Dose of study drug reduced

7.2.2.4 Therapy

- **None:** No new treatment instituted
- **Medication:** New treatment initiated as a direct result of AE
- **Other:** Other action required

7.2.2.5 Outcome

- **Recovered/resolved:** Recovered or resolved
- **Recovered/resolved with sequelae:** Recovered or resolved with sequelae
- **Not recovered/not resolved:** Not recovered or not resolved
- **Fatal:** Death due to an AE
- **Unknown:** Unknown

7.2.2.6 Seriousness

- **Not serious**
- **Serious**

7.2.3 Definition of Unexpectedness

An AE, the nature or severity of which is not consistent with the information provided in the Reference Safety Information section of the current trofinetide Investigator's brochure.

7.3 Time Period and Frequency for Event Assessment and Follow-Up

Adverse events will be recorded from the time informed consent is obtained through the safety follow-up period. All AEs must be either resolved or stable at the end of the safety follow-up period. If ongoing at the end of the safety follow-up period, the subject should be referred for appropriate treatment.

In the event that a subject discontinues and has an ongoing AE at the time of discontinuation (Section 4.4.2) or is withdrawn from the study because of an AE, the subject should be followed and treated by the Investigator until the AE has resolved, stabilized, or a new chronic baseline has been established.

7.3.1 Adverse Event Reporting

The Investigator must record all observed AEs and all reported AEs. At each visit, the Investigator should ask the subject a nonspecific question (e.g., "Have you noticed anything different since your last visit?") to assess whether any AEs have been experienced since the last report or visit.

Note that any use of medication (and specifically any newly prescribed medication) during the course of a study may indicate the occurrence of an AE that may need to be recorded on both the AE and the concomitant medication page.

All AEs, serious and not serious, will be recorded on the AE eCRF page using appropriate medical terminology. Severity and relationship to study drug will be assessed by the Investigator.

When possible, clinical AEs should be described by diagnosis and not by symptoms (e.g., “cold” or “seasonal allergies” instead of “runny nose”).

All AEs, *whether or not related to the study drug*, must be fully and completely documented on the AE eCRF and in the subject’s notes.

7.3.2 Serious Adverse Event Reporting

The reporting of SAEs by the Sponsor or designee to the regulatory authorities is a regulatory requirement. Each regulatory authority has established a timetable for reporting SAEs based upon established criteria.

Serious AEs must be reported within 24 hours of discovery to the Sponsor or its designee; use the appropriate form for initial and/or follow-up reporting.

At a minimum, events identified by the Sponsor to require expedited reporting as serious, unexpected, and related to study drug must be brought to the attention of the responsible Institutional Review Board/Ethics Committee (IRB/EC), as per applicable regulations. These will be provided by the Sponsor after their assessment. For European Union member states, the Sponsor or its designee will provide reports of suspected unexpected serious adverse reactions (SUSARs) directly to the ECs, as required by local legislation. In all other countries, it is the Investigator’s responsibility to provide these expedited reports to the responsible IRB/EC. It is also the Investigator’s responsibility to notify the responsible IRB/EC regarding any new and significant safety information.

When an SAE occurs, Investigators will review all documentation related to the event and will complete the paper SAE form (for initial and/or follow-up information) and fax or email (within 24 hours of discovery) to the contact information provided on the SAE form.

Subjects will be followed through the safety follow-up period for 30 days after last dose of study drug for any SAEs and/or other reportable information until such events have resolved or the Investigator, in conjunction with the Sponsor, deems them to be chronic or stable.

In the event of any SAE (other than death), the study subject will be instructed to contact the Investigator (or designee) using the telephone number provided in the ICF. All subjects experiencing an SAE will be seen by the Investigator or designee as soon as is feasible following the report of the SAE.

Serious AEs occurring after the study follow-up period (i.e., 30 days after last dose of study drug) should be reported if in the judgment of the Investigator there is “a reasonable possibility” that the event may have been caused by the product.

SAEs should also be reported to the IRB/EC according to local regulations.

7.3.3 Reporting of Pregnancy

Any subject who becomes pregnant during the study (with or without AEs) must be withdrawn from the study and the pregnancy must be reported on the Pregnancy form within 24 hours of discovery to the Sponsor or its designee. Any subject who becomes pregnant during the study will be followed through the pregnancy outcome.

Any AEs that are the consequence of pregnancy and which meet the criteria for serious should also be reported via the SAE form.

7.3.3.1 Reporting Paternal Drug Exposure

Paternal drug exposure is defined as a father’s exposure to a medicinal product before or during his partner’s pregnancy. Any paternal drug exposure cases must be reported to the Sponsor within 24 hours of discovery via the Pregnancy form. Any AEs that are the consequence of paternal drug exposure and which meet the criteria for serious must also be reported to the Sponsor within 24 hours of discovery via the SAE form. Since no males are enrolling in this study, paternal drug exposure would occur only if a male who was not a study subject ingested study drug.

7.3.4 Reporting of Overdose

An overdose is a deliberate or inadvertent administration of a treatment at a dose higher than the maximum recommended dose per protocol. It must be reported to the Sponsor or designee on the Overdose form within 24 hours of discovery. In addition, all events of overdose are to be captured as protocol deviations.

8 CLINICAL MONITORING

Routine monitoring of study sites is described in Section 11.

Clinical site monitoring is conducted to ensure that the rights and well-being of human subjects are protected, that the reported study data are accurate, complete, and verifiable, and that the conduct of the study is in compliance with the currently approved protocol and amendment(s) as applicable, with GCP, and with applicable regulatory requirements. Details of the study site monitoring process are described in a separate clinical monitoring plan document.

9 STATISTICAL METHODS AND DATA ANALYSIS

9.1 Statistical and Analytical Plans

Statistical methods will be documented in detail in a statistical analysis plan (SAP) to be approved by the Sponsor prior to database lock.

9.2 Statistical Hypotheses

The co-primary endpoints are change from Baseline to Week 12 in RSBQ total score and CGI-I score at Week 12.

Let Δ_{RSBQ} and $\Delta_{CGI\text{-}I}$ be the difference between the trofinetide and placebo groups in the mean change from Baseline to Week 12 in RSBQ total score and in the mean CGI-I score at Week 12 respectively.

RSBQ: The null hypothesis is: $\Delta_{RSBQ} = 0$ and the alternative hypothesis is: $\Delta_{RSBQ} \neq 0$.

CGI-I: The null hypothesis is: $\Delta_{CGI\text{-}I} = 0$ and the alternative hypothesis is: $\Delta_{CGI\text{-}I} \neq 0$.

9.3 Sample Size Determination

The sample size calculation was performed for the co-primary endpoints as a family of two hypothesis tests at an overall two-sided significance level of 0.05. A total sample size of 174 subjects in a 1:1 ratio to trofinetide or placebo was estimated to provide at least 90% power for the hypothesis testing family assuming the following treatment differences (SD) estimated from Phase 2 study data: -4.4 (8) for the mean change from Baseline to Week 12 in the RSBQ total score and -0.5 (0.7) for the CGI-I mean score at Week 12.

The sample size of 174 subjects will provide at least 95% power at a two-sided significance level of 0.05 for each individual hypothesis test within the family. Trofinetide will be claimed to be superior to placebo if both hypothesis tests within the family are shown to be statistically significant at 0.05. Therefore, the overall power to detect a treatment difference on both of the co-primary endpoints will be at least 90% (0.95^2).

Adjusting for an anticipated discontinuation rate of up to 5%, approximately 184 subjects will be randomized in a 1:1 ratio to trofinetide or placebo.

9.4 Subject Populations for Analysis

The following populations will be defined and used in the analysis:

The Safety Analysis Set will consist of all randomized subjects who received at least one dose of study medication. The Safety Analysis Set will be analyzed according to the actual treatment received. The Safety Analysis Set will be used for the safety analysis.

The Full Analysis Set (FAS) will consist of all randomized subjects who received at least one dose of study medication and who have both a Baseline value and at least one post-Baseline value for the RSBQ total score or who have at least one post-Baseline value for the CGI-I score. The FAS will be analyzed according to the treatment they were assigned regardless of the actual treatment received. The Full Analysis Set will be used for the efficacy analysis.

The Per-protocol (PP) Analysis Set will consist of the subjects in the Full Analysis Set who did not have a major protocol violation that would affect interpretation of the efficacy data. The Per-protocol Analysis Set will be defined prior to study unblinding.

The Pharmacokinetic Analysis Set will consist of subjects in the Safety Analysis Set with at least one measurable trofinetide whole blood concentration.

9.5 Statistical Analyses

9.5.1 General Approach

Unless stated otherwise, all statistical tests will be 2-sided using a 5% significance level, leading to 95% (2-sided) confidence intervals. Trofinetide will be claimed to be superior to placebo if both of the hypothesis tests with respect to the co-primary endpoints are shown to be statistically significant in favor of trofinetide.

Continuous measurement results will be reported using the number of subjects with data values, mean, standard error of the mean, median, standard deviation, minimum, and maximum. For each categorical outcome, the number and percentage of subjects in each category will be reported.

A hierarchical approach will be used to control for the multiple endpoints (co-primary and secondary). Details will be provided in the SAP.

9.5.2 Primary Analyses

The co-primary efficacy endpoints will be analyzed using a mixed model for repeated measures (MMRM). An unstructured covariance matrix will be used and the Kenward-Roger approximation will be used to adjust the denominator degrees of freedom. The treatment comparisons will be based on the difference in least squares means at Week 12.

For the change from Baseline in the RSBQ total score, the MMRM model will include effects for treatment group, age group (5-10 years old, 11-15 years old, and 16-20 years old), Baseline RSBQ severity (<35 total score and ≥35 total score), visit, Baseline RSBQ total score, and interactions for treatment group by visit and Baseline RSBQ total score by visit.

For the CGI-I score, the MMRM model will include effects for treatment group, age group (5-10 years old, 11-15 years old, and 16-20 years old), visit, Baseline RSBQ severity

(<35 total score and ≥35 total score), Baseline CGI-S score, and interactions for treatment group by visit and Baseline CGI-S score by visit.

Sensitivity analyses will be performed to assess the impact of missing data, including analyses based on a missing not at random assumption.

Complete details of the efficacy analyses will be specified in the statistical analysis plan (SAP).

9.5.3 Secondary Analyses

The key secondary endpoint, change from baseline to Week 12 in the CSBS-DP-IT Social Composite Score, will be analyzed using a MMRM method with effects for treatment group, age group (5-10 years old, 11-15 years old, and 16-20 years old), visit, Baseline RSBQ severity (<35 total score and ≥35 total score), Baseline CSBS-DP-IT Social score, and interactions for treatment group by visit and Baseline CSBS-DP-IT Social score by visit. An unstructured covariance matrix will be used and the Kenward-Roger approximation will be used to adjust the denominator degrees of freedom. The treatment comparisons will be based on the difference in least squares means at Week 12.

For the other secondary endpoints that are assessed at multiple post-Baseline visits, the change from Baseline will be analyzed using a MMRM analysis similar to those described above for the co-primary endpoints. The MMRM model will include effects for treatment group, age group (5-10 years old, 11-15 years old, and 16-20 years old), visit, Baseline RSBQ severity (<35 total score and ≥ 35 total score), Baseline score, and interactions for treatment group by visit and Baseline score by visit.

For the other secondary endpoints that are assessed at a single post-Baseline visit (i.e. Week 12 only), the change from Baseline will be analyzed using an analysis of covariance (ANCOVA) model with effects for treatment group, age group (5-10 years old, 11-15 years old, and 16-20 years old), Baseline RSBQ severity (<35 total score and ≥35 total score), and Baseline score.

9.5.4 Safety Analyses

Safety results will be summarized by treatment group using descriptive statistics. No formal statistical testing will be performed for any of the safety endpoints. Adverse events will be classified into standard terminology using the Medical Dictionary for Regulatory Activities (MedDRA). Treatment-emergent adverse events (TEAEs), TEAEs leading to discontinuation, TEAEs related to study drug, TEAEs by maximum severity, fatal TEAEs, SAEs, and SAEs related to study drug will all be summarized.

Descriptive statistics for ECG, vital signs and weight, and clinical laboratory parameters, including changes from Baseline, will be tabulated by timepoint. Additionally, categorical analyses will be conducted on the incidence of subjects with prolonged QTc intervals and changes in QTc intervals in accordance with International Council on Harmonisation (ICH) guidelines.

Additional safety analysis details will be specified in the SAP.

9.5.5 Pharmacokinetic Analyses

Pharmacokinetic (PK) and efficacy (i.e., PD) measures will be collected from all subjects at the Baseline (Week 0) visit before dosing, at the Baseline (Week 0) visit after dosing, and after dosing at Weeks 2, 6, and 12/EOT.

Whole blood concentration and possible metabolites data for trofinetide will be listed and summarized using descriptive statistics. If data allow, population PK and PK/PD analyses will be performed to further characterize the PK profile and exposure response relationship of trofinetide using measures of safety and efficacy parameters. Trofinetide whole blood concentration data will remain blinded until the unblinding of the clinical database at the end of the study.

The details of the PK and PK/PD analysis will be presented in a separate population PK report and PK/PD report in accordance with a separate data analysis plan (DAP).

9.5.6 Subgroup Analyses

Selected analyses may be performed in subgroups. Details will be provided in the SAP.

9.6 Interim Analyses

No interim analysis is planned in this study.

9.7 Data and Safety Monitoring Board

An independent Data and Safety Monitoring Board (DSMB) will review safety information on a regular basis throughout the study. The DSMB will be independent of the Sponsor and will be empowered to recommend stopping the study due to safety concerns. The DSMB may review blinded, unblinded, or partially unblinded data, but the Sponsor and the Investigators will remain blinded to the data provided to the DSMB until the official unblinding of the database at the completion of the study. The membership, activities, responsibilities, and frequency of meetings will be described separately in the DSMB charter.

9.8 Measures to Minimize Bias

Eligible subjects will be randomized into one of two treatment groups (trofinetide or placebo) in a 1:1 ratio using an interactive response technology (IRT) system. The randomization will

be stratified by age group (5-10 years old, 11-15 years old, and 16-20 years old) and Baseline RSBQ severity (<35 total score and ≥35 total score). The assignments will be based on a pre-generated permuted-block randomization schedule. Blinding will be assured by restricting access of Investigators and Sponsor personnel and/or designee to the treatment codes and providing identical packaging for the trofinetide and placebo treatments.

9.9 Breaking the Study Blind/Subject Code

For the final analysis, the treatment codes for all subjects will be released to the Sponsor after all subjects have completed the study and the clinical database is locked.

For DSMB safety reviews, the treatment codes will be released to an independent statistician/programmer to produce unblinded statistical outputs. The Sponsor and the Investigators will remain blinded.

Unblinding of individual treatment assignment during the study is discouraged. The Investigator may break the blind in the event of a medical emergency if it is considered necessary for the care of the subject. The Investigator should attempt whenever possible to contact the Medical Monitor before unblinding a subject's treatment to discuss the event. Lack of Medical Monitor contact does not preclude the Investigator from unblinding the subject. In an emergency situation, the subject's treatment assignment may be obtained by the Investigator from the IRT system. Details of the process to be followed are provided in a separate IRT manual. In the event that the IRT system is used to perform a code break, the Sponsor or designee will be notified immediately via an automated notification from the IRT system that an unblinding has occurred. The notification only alerts the Sponsor or designee that the unblinding occurred and does not include any information about the unblinded subject's treatment assignment.

10 STUDY MANAGEMENT AND DATA COLLECTION

10.1 Data Collection and Management Responsibilities

All documents required for the conduct of the study as specified in the ICH GCP guidelines will be maintained by the Investigator in an orderly manner and made available for monitoring and/or auditing by the Sponsor and regulatory authorities.

The Investigator and institution must permit authorized representatives of the Sponsor or designees (including monitors and auditors), regulatory authorities (including inspectors), and the IRB/EC direct access to source documents (such as original medical records). Direct access includes permission to examine, analyze, verify, and reproduce any records and reports that are needed for the evaluation of the study. The Investigator must ensure the

reliability and availability of source documents from which the information on the eCRF was derived.

10.2 Source Documents

All study specific information obtained at each study visit must be recorded in the subject's record (source documentation), and then entered into a validated electronic data capture (EDC) database by trained site personnel. The source documentation may consist of source notes captured by site personnel and the caregiver diaries, as well as laboratory reports, ECG reports, and electronic source data.

10.3 Case Report Forms

Subject data required by this protocol are to be recorded in an EDC system on eCRFs. The Investigator and his or her site personnel will be responsible for completing the eCRFs. The Investigator is responsible for the accuracy and reliability of all the information recorded on the eCRFs. All information requested on the eCRFs needs to be supplied, including subject identification data, visit date(s), assessment values, etc., and any omission or discrepancy will require explanation. All information on eCRFs must be traceable to source documentation at the site.

10.4 Confidentiality

The Investigator must ensure that each subject's anonymity is maintained as described below. On the eCRFs or other documents submitted to the Sponsor or designees, subjects must be identified by a subject identification number only. Subject identifiers uniquely identify subjects within the study and do not identify any person specifically. Documents that are not for submission to the Sponsor or designees (e.g., signed ICFs) should be kept in strict confidence by the Investigator in compliance with Federal regulations or other applicable laws or ICH guidance on GCP. Data collection and handling should comply with the European Union General Data Protection Regulation (EU GDPR), where applicable. ACADIA has assigned a Data Protection Officer (DPO) as per the EU GDPR.

10.5 Study Records Retention

Investigators are required to maintain all essential study documentation as per ICH GCP guidelines. This includes, but is not limited to, copies of signed, dated and completed eCRFs, documentation of eCRF corrections, signed ICFs, subject-related source documentation, and adequate records for the receipt and disposition of all study drug. Investigators should maintain all essential study documentation, for a period of at least 2 years following the last approval of marketing application in an ICH region (US, Europe, and Japan), or until at least 2 years after the drug investigational program is discontinued, unless a longer period is

required by applicable law or regulation. Only the Sponsor can notify an Investigator or vendor when any records may be discarded. Investigators should contact the Sponsor before destroying any files.

10.6 Protocol Exceptions and Deviations

No prospective entry criteria protocol deviations are allowed; all subjects must meet all eligibility criteria in order to participate in the study.

Protocol waivers for eligibility will not be granted by the Sponsor under any circumstances. If, during the course of a subject's post-enrollment participation in the trial it is discovered that the subject did not meet all eligibility criteria, he or she will be discontinued, unless the discontinuation presents an unacceptable medical risk. The justification to allow the subject to continue in the trial will be made by the Sponsor, with medical input from the Investigator, and will be documented. If allowed to remain in the trial, this will be reported as a major protocol deviation and not a waiver. All follow-up safety assessments must be completed and documented as outlined in the protocol (Section 3.1.3). The Investigator must report any protocol deviation to the Sponsor and, if required, to the IRB/EC in accordance with local regulations, within reasonable time.

10.7 Protocol Amendments

Changes to the protocol may be made only by the Sponsor (with or without consultation with the Investigator). All protocol modifications must be submitted to the site IRB/EC in accordance with local requirements and, if required, to regulatory authorities, as either an amendment or a notification. Approval for amendments must be awaited before any changes can be implemented, except for changes necessary to eliminate an immediate hazard to trial subjects, or when the changes involve only logistical or administrative aspects of the trial. No approval is required for notifications.

11 STUDY MONITORING, AUDITING, AND INSPECTING

11.1 Quality Control and Quality Assurance

The Sponsor or designees and regulatory authority inspectors are responsible for contacting and visiting the Investigator for the purpose of inspecting the facilities and, upon request, inspecting the various records of the trial (e.g., eCRFs and other pertinent data) provided that subject confidentiality is respected.

The Sponsor's or designee's monitor is responsible for inspecting the eCRFs at regular intervals throughout the study to verify adherence to the protocol; completeness, accuracy, and consistency of the data; and adherence to local regulations on the conduct of clinical

research. The monitor should have access to subject medical records and other study-related records needed to verify the entries on the eCRFs.

The Investigator agrees to cooperate with the monitor to ensure that any problems detected in the course of these monitoring visits are resolved.

In accordance with ICH guidance on GCP and the Sponsor's audit plans, a certain percentage of sites participating in this study will be audited. These audits may include a review of site facilities (e.g., pharmacy, drug storage areas, and laboratories) and review of study-related records may occur in order to evaluate the trial conduct and compliance with the protocol, ICH guidance on GCP, and applicable regulatory requirements.

The Sponsor's or designee's representatives, regulatory authority inspectors and IRB/EC representatives who obtain direct access to source documents should also respect subject confidentiality, taking all reasonable precautions in accordance with applicable regulatory requirements to maintain the confidentiality of subjects' identities.

11.2 Risk Management

The Sponsor utilizes the ICH E6 (GCP) Revision 2 risk management approach that includes methods to assure and control the quality of the trial proportionate to the risks inherent in the trial and the importance of the information collected. The intent is that all aspects of this trial are operationally feasible and that any unnecessary complexity, procedures, and data collection are avoided. The Sponsor's risk management approach includes the following activities with a focus on critical processes and critical study data:

- Risk Identification: risks to critical trial processes, governing systems, investigational product, trial design, data collection, and recording are identified.
- Risk Evaluation: identified risks are evaluated by considering the following factors: (a) likelihood of occurrence, (b) impact on human subject protection and data integrity, and (c) detectability of errors.
- Risk Control: risks that can be reduced (e.g., mitigating) or can be accepted are differentiated. Risk mitigation activities are incorporated in protocol design and implementation, study plans, training, processes, and other documents governing the oversight and execution of study activities. Where possible, predefined quality tolerance limits are to be defined to identify systematic issues that can impact subject safety or data integrity and deviations from the predefined quality tolerance limits will trigger an evaluation and possibly an action. Contingency plans are developed for issues with a high risk factor that cannot be avoided.

- Periodic risk review, communication, and escalation of risk management activities are ongoing during trial execution and risk outcomes are reported in the clinical study report (CSR).

12 ETHICAL CONSIDERATIONS

12.1 Ethical Standard

The study will be conducted in compliance with the protocol, the Declaration of Helsinki, ICH GCP, and other applicable regulatory requirements (e.g., Serious Breach reporting, urgent safety measures, and EU GDPR).

The study will be performed in accordance with the US Health Insurance Portability and Accountability Act (HIPAA) regulations, US FDA GCP Regulations (US CFR 21 parts 50, 54, 56, and 312), and ICH guidance on GCP (E6) and clinical safety data management (E2A).

In accordance with Directive 75/318/EEC, as amended by Directive 91/507/EEC, the final clinical study report will be signed by an Investigator and/or Coordinating Investigator who will be designated prior to the writing of the clinical study report.

12.2 Institutional Review Board/Ethics Committee

The Investigator or designee will provide the IRB/EC with all requisite material, including a copy of the protocol, informed consent, and any subject information or advertising materials. The study will not be initiated until the IRB/EC provides written approval of the protocol and the informed consent and until approved documents have been obtained by the Investigator and copies received by the Sponsor. All amendments will be sent to the IRB/EC for information (minor amendment) or for submission (major amendment) before implementation. The Investigator will supply the IRB/EC and the Sponsor with appropriate reports on the progress of this study, including any necessary safety updates, in accordance with the applicable government regulations and in agreement with policy established by the Sponsor.

12.3 Informed Consent Process

In accord with the provisions of the US CFR 21 part 50, and since this study involves greater than minimal risk but presents the prospect of direct benefit to all subjects enrolled, consent shall be obtained from an LAR, typically a guardian, or at least one parent, in accordance with local IRB requirements. Minors will be given the opportunity to assent to participation if and when they are considered capable of doing so by the PI and per local IRB requirements.

Properly executed, informed consent/assent must be obtained from each LAR/subject prior to any screening procedures. The LAR is defined as ‘An individual or judicial or other body

authorized under applicable law to consent on behalf of a prospective subject to the subject's participation in the procedures involved in the research' (US CFR 21 part 50).

For subjects who are minors, written informed consent will be obtained from the LAR. For subjects who are not minors, written informed consent will be obtained from the LAR or the subject if deemed able by the Investigator. When consent is being provided by an LAR, subject assent for participation should be documented, when possible. Assent is the affirmative agreement to participate in the research of a minor or of an adult who does not have the capacity to consent. If written assent is not possible, verbal assent is allowed and must be documented. If subject assent is not possible, then the site must document rationale for not being able to provide written or documented subject assent.

If a subject's 18th birthday takes place during the study and the subject is deemed able to consent by the Investigator, the subject should sign the informed consent. Reconsenting should take place if required by and in accordance with IRB or EC policy and applicable local law.

The subject's caregiver must also provide informed consent regarding their participation in the study prior to participating in any study procedures.

The informed consent must, at a minimum, include the elements of consent described in the ICH guidance on GCP and the US CFR 21 part 50.25. A copy of the ICF planned for use will be reviewed by the Sponsor or designee for acceptability and must be submitted by the Investigator or designee together with the protocol, to the appropriate IRB/EC for review and approval prior to the start of the study at that investigational site. Consent forms must be in a language fully comprehensible to the LAR of the prospective subject. The Investigator must provide the Sponsor or designee with a copy of the IRB/EC letter approving the protocol and the ICF before the study drug supplies will be shipped and the study can be initiated.

The consent form must be revised if new information becomes available during the study that may be relevant to the subject. Any revision must be submitted to the appropriate IRB/EC for review and approval in advance of use.

12.3.1 Consent and Other Informational Documents Provided to Subjects

The subject/LAR must be given a copy of the signed informed consent and the original maintained in the designated location at the site.

12.3.2 Consent Procedures and Documentation

It is the Investigator or designee's responsibility to obtain written informed consent from the subject/LAR after adequate explanation of the aims, methods, anticipated benefits, and potential hazards of the study. The LAR must be given ample time to decide about study

participation and opportunity to inquire about details of the study. The IRB/EC-approved consent form must be personally signed and dated by the LAR with subject assent, if possible, and by the person who conducted the informed consent discussion. The Investigator or appropriate site personnel must document the details of obtaining informed consent in the subject's study documents.

The subject's caregiver must also indicate their understanding of the study and their role as a caregiver to the subject during the study. The subject's caregiver must provide written consent prior to any screening visit procedures being performed indicating their agreement to participate in the study in the caregiver role.

Participation in the testing for identification of biomarkers is optional. Informed consent must be obtained, as appropriate, prior to blood draws for this procedure.

Records related to a study subject's participation will be maintained and processed according to local laws, and where applicable, the European Union General Data Protection Regulation (EU GDPR). The consent and study information documentation will include statements describing local and regional requirements concerning data privacy, and who to contact for questions.

13 PUBLICATION PLAN

All publication rights are delineated in the Clinical Study Agreement and/or other separate agreements with the Investigator and/or Institution, as applicable.

14 CONFLICT OF INTEREST POLICY

14.1 Finance, Insurance, and Indemnity

Arrangements for finance, insurance, and indemnity are delineated in the Clinical Study Agreement and/or other separate agreements with the Investigator and/or Institution, as applicable.

15 LITERATURE REFERENCES

Amir RE, Van den Veyver IB, Schultz R, et al. Influence of mutation type and X chromosome inactivation on Rett syndrome phenotypes. *Ann Neurol*. 2000;47:670-679.

Anagnostou E, Jones N, Huerta M, et al. Measuring Social Communication Behaviors as a Treatment Endpoint in Individuals with Autism Spectrum Disorder. *Autism*. 2015;19(5):622-36.

Barnes KV, Coughlin FR, O'Leary HM, et al. Anxiety-like behaviour in Rett syndrome: characteristics and assessment by anxiety scales. *J Neurodev Disord*. 2015:7(1):30.

Bienvenu T, Philippe C, De Roux N, et al. The incidence of Rett syndrome in France. *Pediatr Neurol*. 2006;34(5):372-375.

Buchovecky CM, Turley SD, Brown HM, et al. A suppressor screen in Mecp2 mutant mice implicates cholesterol metabolism in Rett syndrome. *Nat Genet*. 2013;45(9):1013-1020.

Busner J, Targum SD. The clinical global impressions scale: applying a research tool in clinical practice. *Psychiatry.* 2007;Jul;4(7):28-37.

Byiers BJ, Symons FJ. Issues in estimating developmental level and cognitive function in Rett syndrome. In: Hodapp RM ed. International Review of Research in Intellectual and Developmental Disabilities. Waltham, MA: Elsevier Inc.; 2012;43:147-185.

Camfield C, Breau L, Camfield P. Assessing the impact of pediatric epilepsy and concomitant behavioral, cognitive, and physical/neurologic disability: Impact of Childhood Neurologic Disability Scale. *Dev Med Child Neurol*. 2003;45:152-159.

Cass H, Reilly S, Owen L, et al. Findings from a multidisciplinary clinical case series of females with Rett syndrome. *Dev Med Child Neurol*. 2003;45:325-337.

Cianfaglione R, Clarke A, Kerr M, et al. A national survey of Rett syndrome: behavioural characteristics. *J Neurodev Disord*. 2015;7(1):11.

Cianfaglione R, Clarke A, Kerr M, Hastings RP, Oliver C, Felce D. Ageing in Rett syndrome. *J Intellect Disabil Res*. 2016;60(2):182-190.

Clarkson T, LeBlanc J, DeGregorio G, et al. Adapting the Mullen Scales of Early Learning for a standardized measure of cognition in children with Rett syndrome. *Intellect Dev Disabil*. 2017;55(6):419-431.

Djukic A, McDermott MV. Social preferences in Rett syndrome. *Pediatr Neurol*. 2012;46(4):240-242.

Downs J, Bebbington A, Jacoby P, et al. Level of purposeful hand function as a marker of clinical severity in Rett syndrome. *Dev Med Child Neurol*. 2010;52(9):817-823.

Downs J, Bebbington A, Kaufmann WE, Leonard H. Longitudinal hand function in Rett syndrome. *J Child Neurol.* 2011;26(3):334-340.

Ehrhart F, Coort SL, Cirillo E, Smeets E, Evelo CT, Curfs L. New insights in Rett syndrome using pathway analysis for transcriptomics data. *Wien Med Wochenschr*. 2016;166:346-352.

Epstein A, Leonard H, Davis E, et al. Conceptualizing a quality of life framework for girls with Rett syndrome using qualitative methods. *Am J Med Genet A.* 2016;170(3):645-653.

Glaze DG, Neul JL, Kaufmann WE, et al. Double-blind, randomized, placebo-controlled study of trofinetide in pediatric Rett syndrome. *Neurology*. 2019;92(16):e1912-e1925.

Glaze DG, Neul JL, Percy A, et al. A double-blind, randomized, placebo-controlled clinical study of trofinetide in the treatment of Rett syndrome. *Pediatr Neurol*. 2017;76:37-46.

Glaze DG, Percy AK, Skinner S, et al. Epilepsy and the natural history of Rett syndrome. *Neurology*. 2010;74:909-912.

Hite KC, Adams VH, Hansen JC. Recent advances in MeCP2 structure and function. *Biochem Cell Biol*. 2009;87(1):219-227.

ICH Harmonized Tripartite Guideline: Clinical Safety Data Management: Definitions and Standards for Expedited Reporting E2A. International Conference on Harmonisation of Technical Requirements for Registration of Pharmaceuticals for Human Use. October 1994.

Iemmi V, Knapp M, Brown FJ. Positive behavioural support for children and adolescents with intellectual disabilities whose behaviour challenges: an exploration of the economic case. *J Intellect Disabil*. 2015;20(3):281-295.

Ip JPK, Mellios N, Sur M. Rett syndrome: insights into genetic, molecular and circuit mechanisms. *Nat Rev Neurosci*. 2018;19(6):368-382.

Jusko WJ, Chiang ST. Distribution volume related to body weight and protein binding. *J Pharm Sci*. 1982;71(4):469-70.

Kaufmann WE, Tierney E, Rohde CA, et al. Social impairments in Rett syndrome: characteristics and relationship to clinical severity. *J Intellect Disabil Res.* 2012;56(3):233-247.

Kerr AM, Armstrong DD, Prescott RJ, Doyle D, Kearney DL. Rett syndrome: analysis of deaths in the British survey. *Eur Child Adolesc Psychiatry.* 1997;6 Suppl 1:71-74.

Kersting G, Willmann S, Würthwein G, et al. Physiologically based pharmacokinetic modelling of high- and low-dose etoposide: from adults to children. *Cancer Chemother Pharmacol*. 2012 Feb;69(2):397-405.

Khwaja O, Ho E, Barnes KV, et al. Safety, pharmacokinetics, and preliminary assessment of efficacy of mecasermin (recombinant human IGF-1) for the treatment of Rett syndrome. *Proc Natl Acad Sci U.S.A*. 2014;111(12):4596-4601.

Kriaucionis S, Bird A. DNA methylation and Rett syndrome. *Hum Mol Genet*. 2003;12 Spec No 2:R221-227.

Krishnaraj R, Ho G, Christodoulou J. RettBASE: Rett syndrome database update. *Hum Mutat.* 2017;38(8):922-931.

Lane J, Salter A, Jones NE, et al. Assessment of Caregiver Inventory for Rett syndrome. *J Autism Dev Disord*. 2017;47(4). 1102-1112.

Larsson G, Lindström B, Witt Engerström I. Rett syndrome from a family perspective: the Swedish Rett Center Survey. *Brain Dev.* 2005;27 Suppl 1:S14-19.

Lee JY, Leonard H, Piek JP, Downs J. Early development and regression in Rett syndrome. *Clin Genet.* 2013;84(6):572–576.

Monrós E, Armstrong J, Aibar E, Poo P, Canos I, Pineda M. Rett syndrome in Spain: mutation analysis and clinical correlations. *Brain & Development*. 2001;23:S251-S253.

Motil KJ, Caeg E, Barrish JO, et al. Gastrointestinal and nutritional problems occur frequently throughout life in girls and women with Rett syndrome. *J Pediatr Gastroenterol Nutr*. 2012;55(3):292-298.

Mount RH, Charman T, Hastings RP, Reilly S, Cass H. Features of autism in Rett syndrome and severe mental retardation. *J Autism Dev Disord.* 2003;33(4):435-442.

Mount RH, Charman T, Hastings RP, Reilly S, Cass H. The Rett Syndrome Behaviour Questionnaire (RSBQ): Refining the behavioural phenotype of Rett syndrome. *J Child Psychol Psychiatry*. 2002;43(8):1099–1110.

Mount RH, Hastings RP, Reilly S, Cass H, Charman T. Behavioural and emotional features in Rett syndrome. *Disabil Rehabil.* 2001;23(3-4):129-138.

Neul JL, Benke TA, Marsh ED, et al. The array of clinical phenotypes of males with mutations in Methyl-CpG binding protein 2. *Am J Med Genet B Neuropsychiatr Genet*. 2019;180(1):55-67.

Neul JL, Glaze D, Percy A, et al. Improving Treatment Trial Outcomes for Rett Syndrome: the development of Rett-specific anchors for the Clinical Global Impression Scale. *J Child Neurol*. 2015;30(13):1743-1748.

Neul JL, Lane JB, Lee HS, et al. Developmental delay in Rett syndrome: data from the natural history study. *J Neurodevelop Dis*. 2014;(30):2-9.

Neul JL, Kaufmann WE, Glaze DG, et al. Rett syndrome: revised diagnostic criteria and nomenclature. *Ann Neurol.* 2010;68(6):944-950.

Neul JL, Fang P, Barrish J, et al. Specific mutations in methyl-CpG-binding protein 2 confer different severity in Rett syndrome. *Neurology.* 2008;70(16):1313-1321.

Novak M, Guest C. Application of a multidimensional Caregiver Burden Inventory. *Gerontologist*. 1989;29(6):798-803.

O'Leary HM, Kaufmann WE, Barnes KV, et al. Placebo controlled crossover efficacy assessment of mecasermin for the treatment of Rett syndrome. *Ann Clin Transl Neurol* 2018;5(3):323-332.

Oosterholt SP, Horrigan J, Jones N, Glass L, Della Pasqua O. Population pharmacokinetics of NNZ-2566 in healthy subjects. *Eur J Pharm Sci*. 2017;15;109S:S98-107.

Palacios-Ceña D, Famoso-Pérez P, Salom-Moreno J, et al. "Living an Obstacle Course": A Qualitative Study Examining the Experiences of Caregivers of Children with Rett Syndrome. *Int. J Environ Res Public Health*. 2018;16(1):41.

Percy AK, Lane J, Annese F, Warren H, Skinner SA, Neul JL. When Rett syndrome is due to genes other than MECP2. *Transl Sci Rare Dis*. 2018;3(1):49-53.

Percy AK, Lee HS, Neul JL, et al. Profiling scoliosis in Rett syndrome. *Pediatr Res.* 2010;67(4):435-439.

Perry A, Sarlo-McGarvey, Haddad, C. Brief report: cognitive and adaptive functioning in 28 girls with Rett syndrome. *J Autism Dev Disord*. 1991;21(4):551-556.

Piana C, Zhao W, Adkison K, et al. Covariate effects and population pharmacokinetics of lamivudine in HIV-infected children. *Br J Clin Pharmacol*. 2014;77(5):861-72.

Robertson L, Hall SE, Jacoby P, Ellaway C, de Klerk N, Leonard H. The association between behaviour and genotype in Rett syndrome using the Australian Rett syndrome database. *Am J Med Genet*. 2006;141B(2):177-183.

Rose SA, Djukic A, Jankowski JJ, Feldman JF, Fishman I, Valicenti-McDemott M. Rett syndrome: an eye-tracking study of attention and recognition memory. *Dev Med Child Neurol*. 2013;55(4):364-371.

Samaco RC, Neul JL. Complexities of Rett syndrome and MeCP2. *J Neurosci*. 2011;31(22):7951-7959.

Shovlin S, Tropea D. Transcriptome level analysis in Rett syndrome using human samples from different tissues. *Orphanet J Rare Dis*. 2018;13(1):113.

Thompson RH, Iwata BA. A descriptive analysis of social consequences following problem behavior. *J Appl Behav. Anal*. 2001;34(2):169-178.

Tropea D, Giacomettim E, Wilson NR, et al. Partial reversal of Rett Syndrome-like symptoms in MeCP2 mutant mice. *PNAS*. 2009;106:2029-2034.

Urbanowicz A, Downs J, Girdler SJ, Ciccone NA, Leonard H. An exploration of the use of eye gaze and gestures in females with Rett syndrome. *J Speech Lang Hear Res.* 2016;59(6):1373-1383.

Urbanowicz A, Downs J, Girdler SJ, Ciccone NA, Leonard H. Aspects of speech language abilities are influenced by *MECP2* mutation type in girls with Rett syndrome. *Am J Med Genet.* 2015;167A(2):354-362.

West PR, Amaral DG, Bais P, et al. Metabolomics as a tool for discovery of biomarkers of autism spectrum disorder in the blood plasma of children. *PLoS One*. 2014;9(11):e112445.

Wetherby AM, Guthrie W, Woods J, et al. Parent-implemented social intervention for toddlers with autism: an RCT. *Pediatrics*. 2014;134(6):1084-1093.

Wetherby AM, Allen L, Cleary J, Kublin K, Goldstein H. Validity and reliability of the communication and symbolic behavior scales developmental profile with very young children. *J Speech Lang Hear Res*. 2002;45(6):1202-1218.

Woodyatt GC, Ozanne AE. A longitudinal study of cognitive skills and communication behaviours in children with Rett syndrome. *J Intellect Disabil Res*. 1993;37(Pt 4):419-435.

Yasui DH, Xu H, Dunaway KW, Lasalle JM, Jin LW, Maezawa I. MeCP2 modulates gene expression pathways in astrocytes. *Mol Autism.* 2013;4(1):3.

Young DJ, Bebbington A, Anderson A, et al. The diagnosis of autism in a female: could it be Rett syndrome? *Eur J Pediatr.* 2008;167(6):661-669.

16 APPENDICES

Appendix A Rett Syndrome Diagnostic Criteria

**RTT Diagnostic Criteria 2010**<br>1. Consider diagnosis when postnatal deceleration of head growth observed.
Required for typical or classic RTT:<br>1. A period of regression followed by recovery or stabilization[a]<br>2. All main criteria and all exclusion criteria<br>3. Supportive criteria are not required, although often present in typical RTT
Required for atypical or variant RTT:<br>1. A period of regression followed by recovery or stabilization[a]<br>2. At least 2 out of the 4 main criteria<br>3. 5 out of 11 supportive criteria
**Main Criteria:**<br>1. Partial or complete loss of acquired purposeful hand skills.<br>2. Partial or complete loss of acquired spoken language[b]<br>3. Gait abnormalities: Impaired (dyspraxic) or absence of ability.<br>4. Stereotypic hand movements such as hand wringing/squeezing, clapping/tapping, mouthing and washing/rubbing automatisms
**Exclusion Criteria for typical RTT:**<br>1. Brain injury secondary to trauma (peri- or postnatally), neurometabolic disease, or severe infection that causes neurological problems[c]<br>2. Grossly abnormal psychomotor development in first 6 months of life[d]
**Supportive Criteria for atypical RTT**:[e]<br>1. Breathing disturbances when awake<br>2. Bruxism when awake<br>3. Impaired sleep pattern<br>4. Abnormal muscle tone<br>5. Peripheral vasomotor disturbances<br>6. Scoliosis/kyphosis<br>7. Growth retardation<br>8. Small cold hands and feet<br>9. Inappropriate laughing/screaming spells<br>10. Diminished response to pain<br>11. Intense eye communication - “eye pointing”

Table footnotes provided on next page

Source: Neul et al. 2010

[a] Because *MECP2* mutations are now identified in some individuals prior to any clear evidence of regression, the diagnosis of "possible" RTT should be given to those individuals under 3 years old who have not lost any skills but otherwise have clinical features suggestive of RTT. These individuals should be reassessed every 6–12 months for evidence of regression. If regression manifests, the diagnosis should then be changed to definite RTT. However, if the child does not show any evidence of regression by 5 years, the diagnosis of RTT should be questioned.

[b] Loss of acquired language is based on best acquired spoken language skill, not strictly on the acquisition of distinct words or higher language skills. Thus, an individual who had learned to babble but then loses this ability is considered to have a loss of acquired language.

[c] There should be clear evidence (neurological or ophthalmological examination and MRI/CT) that the presumed insult directly resulted in neurological dysfunction.

[d] Grossly abnormal to the point that normal milestones (acquiring head control, swallowing, developing social smile) are not met. Mild generalized hypotonia or other previously reported subtle developmental alterations during the first six months of life is common in RTT and do not constitute an exclusionary criterion.

[e] If an individual has or ever had a clinical feature listed it is counted as a supportive criterion. Many of these features have an age dependency, manifesting and becoming more predominant at certain ages. Therefore, the diagnosis of atypical RTT may be easier for older individuals than for younger. In the case of a younger individual (under 5 years old) who has a period of regression and ≥2 main criteria but does not fulfil the requirement of 5/11 supportive criteria, the diagnosis of "probably atypical RTT" may be given. Individuals who fall into this category should be reassessed as they age and the diagnosis revised accordingly.
